# Advanced Flame Spray Pyrolysis (FSP) Technologies for Engineering Multifunctional Nanostructures and Nanodevices

**DOI:** 10.3390/nano13233006

**Published:** 2023-11-23

**Authors:** Christos Dimitriou, Pavlos Psathas, Maria Solakidou, Yiannis Deligiannakis

**Affiliations:** Laboratory of Physical Chemistry of Materials & Environment, Department of Physics, University of Ioannina, 45110 Ioannina, Greece

**Keywords:** flame spray pyrolysis, TRL, complex assemblies, multifunctional nanomaterials/nanodevices, oxygen-deficiency process, double nozzle, perovskites, non-oxides, quantum dots, plasmonics, nanofilms

## Abstract

Flame spray pyrolysis (FSP) is an industrially scalable technology that enables the engineering of a wide range of metal-based nanomaterials with tailored properties nanoparticles. In the present review, we discuss the recent state-of-the-art advances in FSP technology with regard to nanostructure engineering as well as the FSP reactor setup designs. The challenges of in situ incorporation of nanoparticles into complex functional arrays are reviewed, underscoring FSP’s transformative potential in next-generation nanodevice fabrication. Key areas of focus include the integration of FSP into the technology readiness level (TRL) for nanomaterials production, the FSP process design, and recent advancements in nanodevice development. With a comprehensive overview of engineering methodologies such as the oxygen-deficient process, double-nozzle configuration, and in situ coatings deposition, this review charts the trajectory of FSP from its foundational roots to its contemporary applications in intricate nanostructure and nanodevice synthesis.

## 1. Introduction

Flame spray pyrolysis (FSP) is an industrially scalable technique for the synthesis of nanomaterials, which allows to produce many types of metal, or metal-oxide nanoparticles with tailored physicochemical properties [1]. At the heart of the FSP technology lies an intricate process design, involving precursor atomization, combustion, and nanoparticle formation [2]. This enables swift, single-step synthesis, eliminating the need for post-production treatments commonly required in other methodologies. Recent advancements have further elevated the prominence of FSP in the development of nanodevices, i.e., where nanoparticles can be in situ incorporated in complex functional arrays [3]. Thus, FSP not only revolutionizes nanomaterials’ production but, with recent innovations, also paves the way for the next generation of nanodevices [4]. In the present review article, we provide an updated overview of the current state-of-the-art in FSP technology regarding novel reactor and process designs, novel material production, and nanodevice engineering.

In the domain of nanotechnology, nanostructure synthesis represents a critical research area, encompassing a diverse range of methodologies alongside FSP. These alternative techniques, including, but not limited to, chemical vapor deposition, sol-gel processing, and electrospinning, offer unique properties in terms of particle size control, morphology, and chemical composition. The selection of an appropriate synthesis method is contingent upon a set of criteria closely related to the intended application of the nanostructures. Factors such as material versatility, environmental impact, synthesis time, and temperature range play a pivotal role in determining the suitability of a technique for specific applications, which vary from drug delivery systems to photovoltaic devices. In Table 1, we enumerate various methodologies employed in the synthesis of nanomaterials, including FSP, and delineate the specific criteria applicable to their utilization. This careful consideration ensures that the synthesis process aligns with the functional requirements of the end application, thereby maximizing the efficacy and utility of the nanostructures produced. Atomic layer deposition (ALD) [5,6] is an alternative to FSP, particularly for engineering thin films. ALD provides atomic-level precision and high-level conformality, producing highly uniform and defect-free films. The downsides include a slower deposition rate, the need for expensive and controlled-environment equipment, and potentially high costs for precursors [5,6].

### 1.1. Integration of Flame Spray Pyrolysis into the Technology Readiness Level (TRL) Scale for Nanomaterial Production

**Figure 1:**
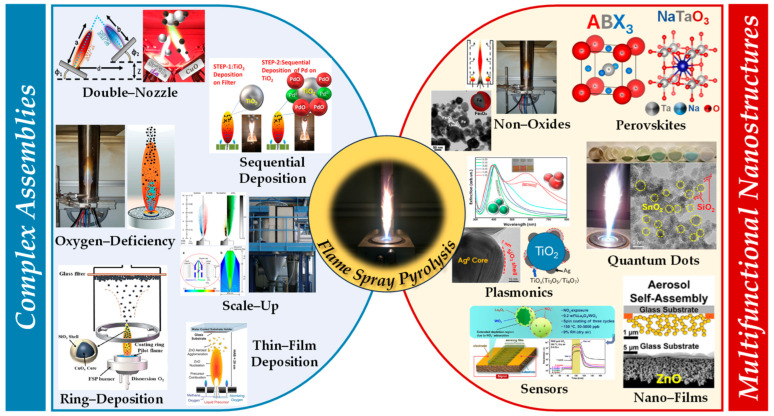
Figure illustrating the complex assemblies in flame spray pyrolysis (FSP) discussed in this review. These include double-nozzle, sequential deposition, oxygen-deficiency process, ring deposition, sequential/thin-film deposition, and scale-up methods. The resultant advanced nanomaterials/nanodevices encompass perovskites, non-oxides, quantum dots, plasmonics, nanofilms, and sensors.

FSP stands out as an innovative and advanced methodology for the synthesis of nanomaterials, which highlights its vital role in producing a wide array of metal oxide nanoparticles with tailored morphologies and compositions [24]. Inherent in FSP, synthesis at elevated temperatures enhances both the crystallinity and physicochemical attributes of the nanoparticles. By adeptly adjusting operational parameters, such as precursor solution concentration, solvent type, flame temperature, oxygen-to-fuel ratio, and particle residence time in the flame zone, researchers can effectively control the nanoparticle size, distribution, and phase composition. Regarding the technology readiness level (TRL) spectrum, FSP for device applications aligns with TRL 6–8 [25]. This placement signifies FSP’s evolution beyond foundational laboratory research [1]. Given the adoption of FSP by certain industries, this positions FSP in the late stages of development and early stages of commercialization, placing it in the TRL 7–8 range (see Figure 1) [25].

### 1.2. Process Design

The process design of FSP [26] begins with the preparation of a metal or metal-organic precursor solution, typically dissolved in an appropriate solvent. This solution undergoes atomization, often facilitated by a high-pressure nozzle, to form a fine spray of droplets [2]. Subsequent ignition of these droplets, often with the aid of an auxiliary flame, leads to the combustion of the solvent and the eventual decomposition of the metal precursors. Within this flame environment, characterized by elevated temperatures, the precursor decomposes, and metal or metal-oxide nanoparticles nucleate and grow [27]. The characteristics of the resultant nanoparticles—size, morphology, crystallinity, and phase composition—can be controlled by diligent choice of the metal precursor, its concentration in the solution, the solvent’s nature, the atomization method, flame temperature, oxygen-to-fuel ratio, and the residence time of particles within the flame. Both inorganic and organic metal salts—including nitrates, acetates, and 2-ethylhexanoates—as well as metalorganic compounds such as acetylacetonates or alkoxides, serve as prevalent precursors [28]. These compounds are soluble in organic solvents, notably xylene (with a standard enthalpy change in combustion [29], $\Delta_{c}H_{298}^{Ø}$ = −4550 kJ mol^−1^), toluene (−3910 kJ mol^−1^), ethanol (−1376 kJ mol^−1^), acetonitrile (−1256 kJ mol^−1^), etc. Another pivotal aspect of the FSP design is its continuous mode of operation that enhances its scalability potential, making it an attractive proposition for industrial applications.

**Figure 2:**
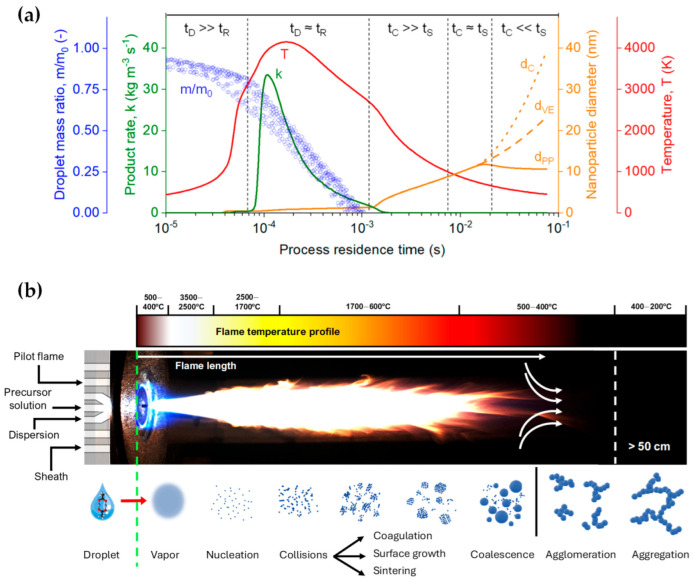
(**a**) Temporal scales in the fabrication of ZrO_2_ nanoparticles via FSP. A time-evolving analysis encompasses the dynamics of the droplet mass ratio, the rate of product formation, nanoparticle diameter, and gas temperature, serving to demarcate distinct phases within the manufacturing process. Reprinted (adapted) with permission from [1]. Copyright 2021 American Chemical Society. (**b**) Visualization of actual FSP flame, depicting the synthesis parameters (pilot flame, precursor solution, dispersion, sheath gas). Concurrently, a graphical representation of the flame’s temperature distribution, congruent with that depicted in (**a**), is presented. Below the flame, a comprehensive elucidation of the droplet-to-particle transformation process in the production of nanoparticles is provided.

Furthermore, the high-temperature synthesis environment ensures rapid crystallization of particles, obviating the need for post-process annealing. Meierhofer et al. [1] delineated the relationship between temperature and process residence time during each phase of the droplet-to-particle formation, as represented by the red line in Figure 2a and the flame temperature profile in Figure 2b. At the nozzle’s apex, temperature fluctuations range from 500 to 400 °C within the initial 10 μs. Adjacent to the capillary tip, the flame’s core registers the peak temperatures, oscillating between 3500 and 2500 °C (Figure 2). At this juncture, the precursor solution vaporizes, initiating the nucleation of the primary particles. Following initial particle formation, particles fuse cohesively within the temperature range of 1700–600 °C during coagulation and sintering processes. Driven by Brownian motion, these particles collide and coalesce, forming larger entities. As the sintered particles move further through the flame and into cooler regions (<600 °C), they can stick together into agglomerates, forming loose clusters. This clustering is due to physical forces, such as van der Waals interactions. Subsequently, these agglomerates transform into aggregates, binding more firmly through chemical (covalent) bonds in the temperature range of 400–200 °C. The FSP setup typically comprises components like liquid atomizers, combustion chambers, flame torches, and substrate holders for potential direct deposition of nanoparticles.

### 1.3. Recent Advancements in Product/Nanodevice Development

**Figure 3:**
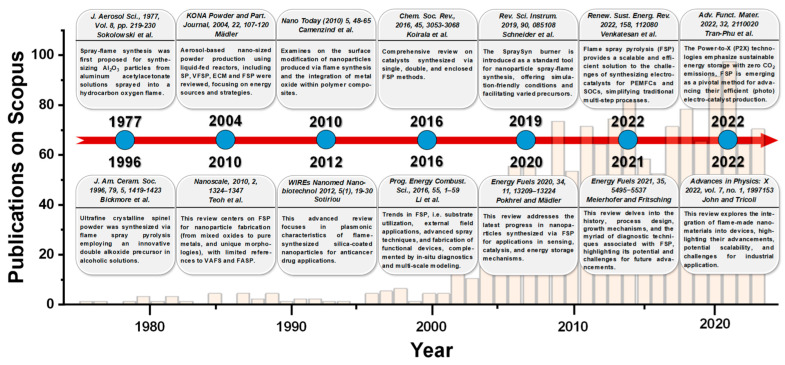
Timeline of the flame spray pyrolysis (FSP) technology, and some pertinent review articles. The bar graph depicts the annual publication frequency (1365 documents in total) from 1977 to 2023, sourced from Scopus using the keyword ‘Flame Spray Pyrolysis’ [1,2,30,31,34,35,36,37,38,39,40,41,42,43].

Figure 3 provides a chronological tracing of the literature articles related to FSP, highlighted by pertinent reviews. Introduced in the 1970s by Sokolowski et al. [30], FSP was utilized for synthesizing Al_2_O_3_ nanoparticles from an aluminum acetylacetonate precursor in a benzene-ethanol solution via an ultrasound nozzle. Despite the initial decline in interest, the technique was refined in the 1990s by Laine and colleagues at the University of Michigan [7,31]. In a pilot-scale FSP reactor, a double-alkoxide (Mg-Al) precursor in an alcoholic solution was employed to yield spinel MgAl_2_O_4_ nanoparticles at rates between 50 and 100 g/h. Concurrently, scientists at Tampere University of Technology employed the FSP method for various metal oxide syntheses and conducted detailed optical diagnostics on the produced aerosols [32]. By the commencement of the 21st century, Pratsinis’s team at the Swiss Federal Institute of Technology in Zürich further adapted FSP, highlighting its potential in catalytic material development [33].

Mädler’s review in 2004 [34] emphasized the increasing use of liquid-fed reactors for the aerosol-based synthesis of nano-sized powders. The rising interest in aerosol processes and the growing demand for various functional metal oxides have accelerated the R&D of these reactors. He examined four primary spray techniques: spray pyrolysis in a tubular reactor (SP), vapor flame reactor spray pyrolysis (VFSP), emulsion combustion method (ECM), and flame spray pyrolysis (FSP), comparing their energy sources and reaction mechanisms. He also outlined methods to produce consistent products and their specific applications [34]. In 2010, Teoh and colleagues [2] presented an exhaustive review focusing on FSP as a method for nanoparticle synthesis, spanning from mixed oxides to pure metals and encompassing specialized morphologies, such as core-shell structures, with minimal references to VAFS and FASP. Conversely, Camenzind and associates [35] delve into the surface functionalization of nanoparticles generated through flame synthesis and the incorporation of metal oxide within polymer composites. Moreover, in 2013, Sotiriou [36] provided an in-depth review emphasizing the plasmonic properties of flame-synthesized silica-coated nanoparticles and their potential applications in anticancer drug delivery.

Koirala et al. in 2016 [37] conducted a thorough examination of catalysts produced through single, double, and enclosed FSP techniques. In the same year, Li and his colleagues [38] detailed advancements in FSP, encompassing substrate usage, applications of external fields, innovative spray methodologies, and the construction of functional apparatus, supplemented by in situ diagnostics and multi-scale simulations. In 2019, Schneider et al. [39] presented the SpraySyn burner as a benchmark instrument for the spray-flame synthesis of nanoparticles. This apparatus offers conditions amenable to simulation and accommodates a variety of precursors. One year later, Pokhrel and Mädler’s review [40] outlined recent advancements in nanoparticles produced through FSP for sensing, catalysis, and energy storage applications, while Meierhofer and Fritsching in 2021 [1] provided a detailed analysis of FSP’s historical context, design, growth mechanisms, and diagnostic methodologies, emphasizing its prospective opportunities and challenges.

Meanwhile, Venkatesan et al. [41] highlighted that FSP offers a scalable and proficient approach to address the complexities of electrocatalyst synthesis for polymer electrolyte membrane fuel cells (PEMFCs) and solid oxide cells (SOCs), streamlining conventional multistage procedures. In 2022, Tran-Phu et al. [42] presented a review on Power-to-X (P2X) technologies, underscoring the significance of sustainable energy storage with zero CO_2_ emissions. Within this context, FSP is identified as a crucial technique for enhancing the production of efficient (photo)electrocatalysts. Ultimately, within that year, John and Tricoli’s review [43] probed the particle formation mechanism, drawing insights from micro-explosions in single droplet experiments across diverse precursor–solvent pairs. The discussion emphasizes the importance of layer fabrication for industrial applications, including gas sensors, catalysis, and energy storage.

Herein in this review, we focus on recent advancements in product and nanodevice development by FSP. In Section 2, we review key aspects of engineering of complex assemblies via FSP, as well as mechanisms and process designs such as the oxygen-deficient process, double-nozzle configuration, in situ coatings, FSP deposition, sequential/nano-film deposition, and scale-up industrial production that enable the creation of intricate nanostructures. In Section 3, we discuss examples of complex functional nanostructures and nanodevices synthesized via FSP, such as perovskites, non-oxides, quantum dots, plasmonics, nanofilms, and sensors unraveling their potential applications and the scientific underpinnings that govern their multifaceted functionality.

## 2. Engineering of Complex Nanoassemblies by Flame Spray Pyrolysis

Complex configurations via FSP encompass a wide range of materials—see details in Section 3. In this section, we review the FSP process principles that can be used to achieve engineering of the advanced nanostructures. In brief, for the sake of the presentation, we can categorize the FSP methodologies as

- Oxygen-deficient FSP process,
- Double-nozzle FSP configuration,
- In situ coatings FSP deposition,
- Sequential/nanofilm FSP deposition.

Finally, we discuss some aspects of scale-up FSP. By this, we refer to the pilot FSP reactors that have been reported so far by various research laboratories and their application in novel nanomaterials engineering.

### 2.1. Oxygen-Deficient FSP Process

The concept of oxygen-deficient synthesis can pertain to anoxic or reduced metal oxides. In the literature, these are referenced as M*_K_*O*_L_*_−x_ where *K* and *L* are the stoichiometry coefficients that determine the stable crystal phase M*_K_*O*_L_*. In this terminology, x signifies the O-deficiency coefficient.

Here, for the sake of the discussion, we classify these materials in three cases:

[i] *O-vacancies generation with no change in the crystal phase*: lack of O atoms from the lattice, compared to the formal stoichiometry of the nominal crystal phase, with no modification of the crystal phase.
[ii] *Generation or reduced metal atoms with no change in the crystal phase*: lack of O atoms from the lattice can stabilize lower-oxidation states of the metal atoms.

Often, cases [i] and [ii] are interlinked since the reduction in individual metal atoms in the lattice can be triggered thermodynamically from the generation of one or more O-vacancies in its immediate vicinity.

[iii] *Stabilization of a reduced crystal phase* via *lack of O atoms*: certain O-deficient metal oxides can stabilize reduced phases. This occurs when a significant fraction part of the metal atoms is reduced. For example, magnetite Fe_3_O_4_, which contains one Fe^2+^ and two Fe^3+^, can be formed from Fe_2_O_3_ (two Fe^3+^) when 1/3 of the Fe^3+^-atoms is reduced to Fe^2+^. Further reduction in all Fe atoms to Fe^2+^ forms the FeO phase, while further reduction to Fe^0^-atoms forms the metallic, zero-valent-iron material. Similarly, Cu_2_O (SnO) is formed when all Cu^2+^ (Sn^4+^) atoms in CuO (SnO_2_) are reduced to the Cu^1+^ (Sn^2+^) state.

**Figure 4:**
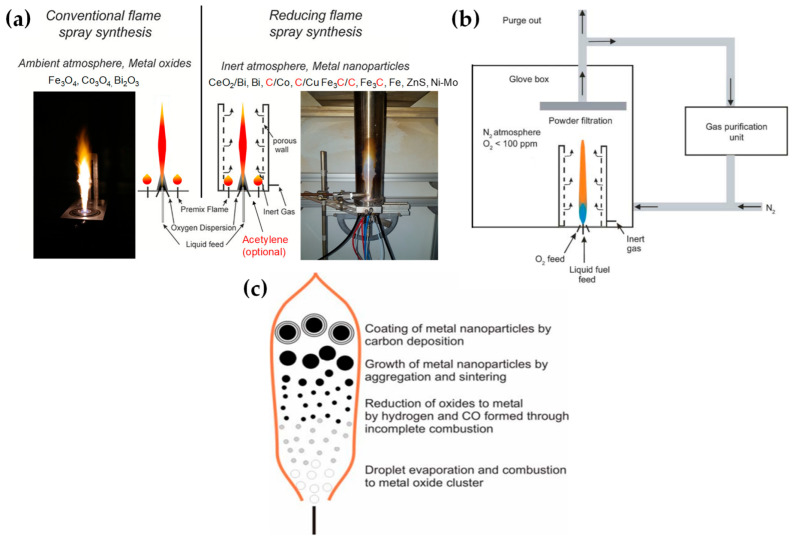
(**a**) Conventional FSP (left) and our reducing FSP (right), where the anoxic flame is produced by in situ introduction of reducing dispersion gas, e.g., CH_4_. (**b**) An anoxic FSP reactor, used by Stark, with the whole reactor enclosed in a glove box filled with an inert atmosphere. By adjusting the gas flow rates, it is possible to achieve highly reduced conditions (O_2_ < 100 ppm). Used with permission of Royal Society of Chemistry from [45]; permission conveyed through Copyright Clearance Center, Inc. (**c**) Schematic depiction of the step-by-step transformation from precursor to oxide, metal, and carbon-coated metal nanoparticles during the reducing flame synthesis process: Initially, the precursor undergoes evaporation and combustion, resulting in oxide nanoparticles. These particles can then be further reduced to their metallic form by H_2_ and CO. Throughout this procedure, the nanoparticles increase in size due to aggregation and sintering. By introducing acetylene, these metal nanoparticles can acquire a carbon coating layer. Reproduced with permission from ref. [47,48]. Copyright 2007 Wiley-VCH.

Table 2 presents a list of literature data on the use of anoxic FSP to engineer nanostructures. The concept of using an oxygen-lean FSP was pioneered by Grass et al. to produce oxygen-deficient metal-oxide particles [44] by placing the FSP nozzle inside a glove box filled with inert nitrogen and regulating the intake of oxidizing gas as illustrated in Figure 4b,c. The dispersion gas mixture in the flame can shift from a CO_2_/H_2_O composition (representing traditional, oxidizing flames, see Figure 4a) to a CO/H_2_/H_2_O mixture (under reducing conditions) [44]. Noble metal nanoparticles, including Pt, Au, Ag, and their alloys, can typically be produced even in oxygen-rich FSP, i.e., due to the thermodynamic preference of the metal state vs. the oxide state by the noble metal atoms. However, creating non-noble metals necessitates a reductive environment. When cobalt or bismuth organic precursors [45], such as cobalt(II)- and bismuth(III)-2-ethylhexanoate (more information in Table 2), are burned in a controlled atmosphere (with O_2_ levels less than 100 ppm) and with a high fuel-to-oxygen ratio (see Figure 4b), it enables the swift production of pure Co and Bi metal nanoparticles, enhancing the conventional flame process. With this experimental setup, Stark et al. have explored the creation of metallic bismuth nanoparticles ensuring no soot formation [46]. While the reducing environment might be beneficial for producing metallic particles on a large scale [45,47], it comes with the risks of incomplete combustion [1]. In the case where the oxygen supply is further constrained, a fine carbonaceous layer tends to form on these metal nanoparticles [47,48]. Using this experimental setup, NiMo nanoalloys [49] and ZnS nanocompounds [50] have been reported.

**Figure 5:**
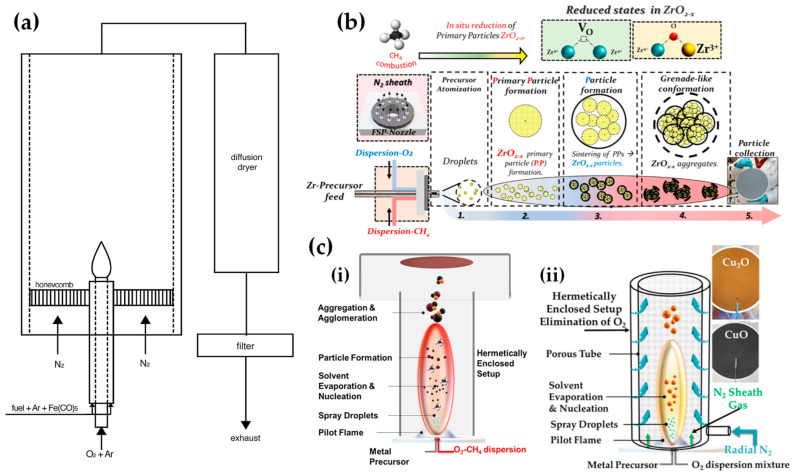
(**a**) Experimental setup of laminar, inverse diffusion flame stabilized on a burner for the synthesis of magnetic iron oxide nanoparticles with reduced oxidation state. Reprinted from [52], with permission from Elsevier. (**b**) The concept of the novel anoxic FSP, as developed by our lab, for ZrO_2−x_ production. Reprinted from [54]. (**c**) (i) Schematic representation of anoxic FSP reactor used for the synthesis of C@Cu_2_O/Cu^0^ nanoparticles. Reprinted from [55]. (ii) Anoxic FSP reactor configuration utilized for creating CuO and Cu_2_O nanomaterials. Reprinted from [55,56].

Strobel and Pratsinis used an oxygen-deficiency FSP process [51] in order to synthesize Fe_2_O_3_, Fe_3_O_4_, and FeO nanoparticles. Their setup featured an FSP nozzle with a metal tube (4 cm in diameter and 40 cm in length) positioned directly above it (as shown in Figure 4b). Situated 20 cm above the FSP nozzle and angled at 45°, an internal mix spray nozzle was directed downward. This nozzle delivered deionized water at a rate of 10 mL/min, dispersed using 5 L/min of N_2_. A different oxygen-deficiency FSP setup for the production of Fe_3_O_4_ nanoparticles may be the utilization of a laminar, inverse diffusion flame [52]. This method takes advantage of the properties of the inverse flame, created when an oxidizer is injected into a flow of surrounding fuel [53]. Contrary to conventional flame approaches, this setup ensures that the iron particle formation occurs in a predominantly reducing atmosphere. As illustrated in Figure 5a, the burner features two concentric brass tubes with specific outer diameters, enclosed within an 11.4 cm diameter acrylic chamber. This chamber is crucial for protecting the flame from ambient air, preventing additional particle oxidation and potential secondary diffusion flame formation due to excess fuel reacting with room air. The oxidizer, either pure O_2_ or an O_2_-Ar mixture, is released from the innermost tube and is encircled by a blend of fuel (methane or ethylene), argon, and iron precursor vapor. A N_2_ flow enveloped the resulting inverse flame.

Recently, we have exemplified a novel anoxic FSP process, to engineer ZrO_2–x_ (see Figure 5b) [54] and C@Cu_2_O/Cu^0^ (see Figure 5c) [55] nanoparticles. Our anoxic FSP concept relies on the combustion of CH_4_ in the dispersion gas. This introduces reducing agents that can modify the primary Zr particle by creating oxygen vacancies (V_O_). XPS and EPR confirm that the increased dispersion of the CH_4_ promotes the formation of oxygen vacancies [54]. A more complicated oxygen-deficiency FSP setup, which includes a dispersion feed consisting of {oxygen (O_2_)–methane (CH_4_)} mixture, in tandem with enclosed FSP flame with radial N_2_, is necessary for the synthesis of non-graphitized carbon/Cu_2_O/Cu^0^ heterojunction (see Figure 5c) [55]. The modification in the dispersion gas mixture leads to increased temperatures and generates reducing agents for the controlled phase transformation from CuO to Cu_2_O and Cu^0^ (see Figure 5c).

**Table 2:** The literature summary of FSP characteristics/conditions for the production of oxygen-deficient nanostructures.

Nano- Structure	FSP Configuration	Precursor(s)	Solvent	Molarity (mol L^−1^)	Precursor Flow (mL min^−1^)	Pilot Flame O_2_/CH_4_ (L min^−1^)	N_2_ Flow (L min^−1^)	Ref.
C-Co	The flame is encased in a porous tube enabling the addition of inert cooling gases and acetylene. The flame is operated in a glove box in an N_2_ atmosphere at an O_2_ < 100 ppm.	Cobalt(II) 2-ethylhexanoate	Tetrahydrofuran		6	2.2/1.2	45	[48,57]
Bi	The spray nozzle was placed in a Glove box fed with N_2_. A sinter metal tube (inner diameter 25 mm) surrounding the flame allowed for N_2_ radial flow.	Bismuth(III) 2-ethylhexanoate	Tetrahydrofuran		6	2.2/1.2	25	[46]
CeO_2_/Bi		Bismuth(III) 2-ethylhexanoate, Cerium(III) octoate	Tetrahydrofuran		6	2.2/1.2	45	[45]
C-Cu	The flame is encased in a porous tube enabling the addition of inert cooling gases. The flame is operated in a glove box in an N_2_ atmosphere at an O_2_ < 100 ppm.	Cu(II)-2-ethylhexanoate	Tetrahydrofuran		4.5	2.2/1.2	45	[47]
Ni-Mo		Ni(II)-ethylhexanoate, Mo(II)-ethylhexanoate	Tetrahydrofuran		6	2.2/1.2	45	[49]
Fe_x_O_y_	FSP nozzle with an Inconel metal tube (ID = 4 cm, length = 40 cm). Water was fed into this nozzle and dispersed by N_2_ gas.	Fe(III) nitrate nonahydrate, Fe(II) naphthenate	2-ethylhexanoic acid/THF/ethanol (2/2/1)	0.9	5	2.5/1	40	[51]
α-Fe/Fe_3_O_4_	Laminar, inverse diffusion flame stabilized on a burner.	Iron pentacarbonyl						[52]
ZrO_2–x_	A single-nozzle FSP reactor featuring an enclosed flame utilizing a mixture of dispersion gases, O_2_ and CH_4_, to establish a reductive reaction environment.	Zirconium(IV) Propoxide	Xylene/acetonitrile (2.2/1.0)	0.25	3	4/2	15	[54]
C@Cu_2_O/ CuO/Cu^0^, Cu_2_O/CuO	A single-nozzle FSP reactor featuring enclosed flame utilizing a mixture of dispersion gases, O_2_ and CH_4_, to establish a reductive reaction environment. A perforated tube permits the introduction of radial N_2_ gas.	Copper(II) nitrate trihydrate	Acetonitrile/ ethylenglycol (1/1)	0.25	3	2/1.2	10	[55,56]

### 2.2. Double-Nozzle FSP Configuration

**Figure 6:**
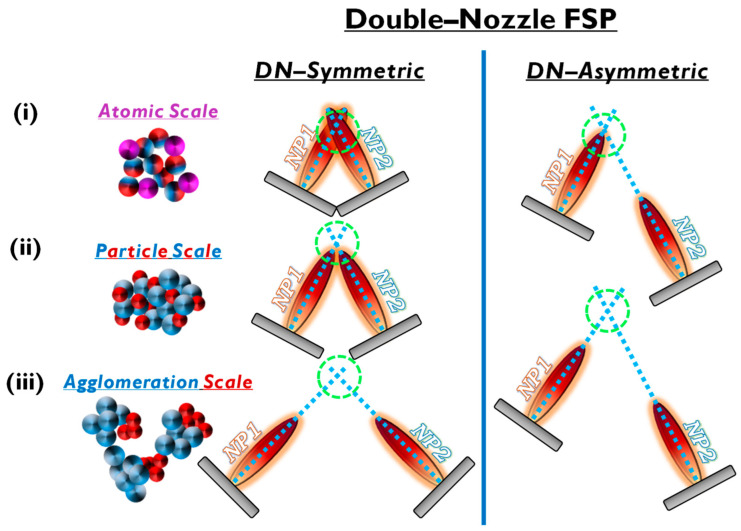
Symmetric and asymmetric DN-FSP configuration for two particle formation regarding the (**i**) atomic, (**ii**) particle, or (**iii**) agglomeration scale.

In the case of mixed structures, e.g., heterojunctions, core-shell compositions, etc., the application of two FSP nozzles that operate in tandem offers advantages. Typical examples include the cases where a nanomaterial (NP1) and a cocatalytic nanomaterial (NP2) are combined. In the conventional single-nozzle FSP, a single precursor contains both the elements of nanomaterial (NP1) and nanomaterial (NP2) and produces the combined material in a single flame (see Figure 6a).

Double-nozzle FSP entails two independent spray flames, with the precursor of NP1 inserted in a different flame than NP2 (see Figure 6). This method unlocks several options for independent size control, mixing, and specific deposition for the two nanomaterials by altering the primary geometrical parameters of distance and intersection of the flames. As shown in Figure 6: (i) At a small flame-intersection distance, where the centers of the flames are in contact, the atoms are in the preliminary stages of crystallization, producing well-mixed particles, tending to be similar to the single-nozzle FSP. In this case, the second flame substantially increases the synthesis overall temperature. (ii) When the intersection occurs after the endpoints of the flames, the materials are well crystallized, resulting in well-mixed primary particles of NP1 and NP2. (iii) At increased intersection distance, the two materials mix at their sintering stage or bigger distances at the agglomeration stage.

**Figure 7:**
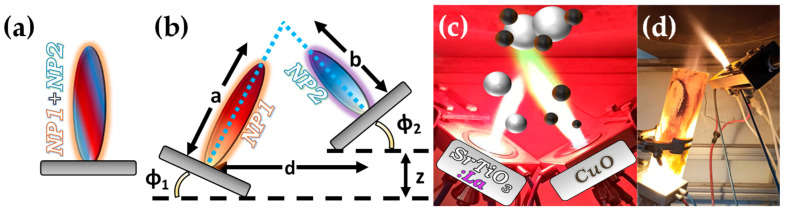
(**a**) Schematic example of SN-FSP where two precursors are mixed before being fed to the flame. (**b**) Geometry parameters of DN-FSP. (**c**) Example of a symmetrical DN-FSP, used for engineering of La-doped SrTiO_3_, with surface deposition of CuO. Reprinted from [58]. (**d**) Example of asymmetrical DN-FSP.

Thus, by changing the geometrical disposition of the two flames via the parameters a, b, d, Φ_1_, Φ_2_, and Z (see Figure 7b), the symmetrical/asymmetrical DN-FSP configuration offers a versatile technology that allows for the control of composite configurations at different synthesis stages, i.e., at the atomic scale, at the particle scale, or the aggregate’s scale (see Figure 6). Table 3 presents a list of the literature data on the use of DN-FSP to engineer nano-heterostructures.

**Figure 8:**
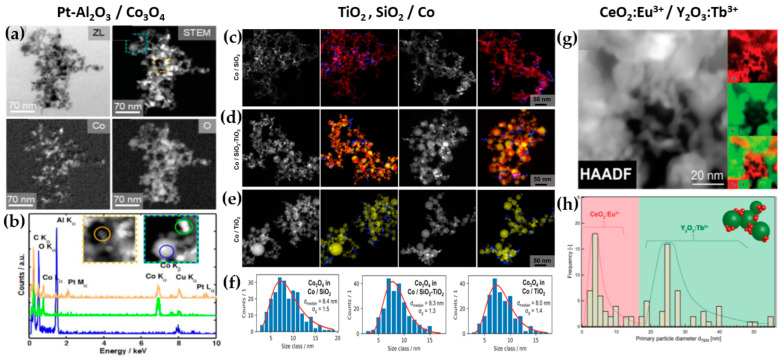
(**a**) TEM images revealing the local distribution of cobalt and oxygen for Pt-Al_2_O_3_/Co_3_O_4_, (**b**) EDX measurements for chemical composition. Reprinted from [64]. DΝ-FSP-prepared (**c**) SiO_2_/Co, (**d**) SiO_2_-TiO_2_/Co, (**e**) and TiO_2_/Co; left images show STEM-HAADF and right images show EDX mappings of the elements Co (blue), Si (red) and Ti (yellow). (**f**) Particle size distributions of Co_3_O_4_ for the materials SiO_2_, SiO_2_-TiO_2_, and TiO_2_. Reproduced with permission from ref. [65]. Copyright 2022 Wiley-VCH. (**g**) STEM-HAADF of the nano-mixed CeO_2_:Eu^3+^/Y_2_O_3_:Tb^3+^ and its elemental mapping for Ce in red and Y in green, (**h**) d_TEM_ distribution of CeO_2_:Eu^3+^ and Y_2_O_3_:Tb^3+^. Reprinted from [66], with permission from Elsevier.

**Al_2_O_3_:** DN-FSP was first implemented by Strobel et al. [59], producing in one nozzle Al_2_O_3_ and in the second nozzle Pt/BaCO_3_, thus forming individual Al_2_O_3_ and monoclinic BaCO_3_ nanoparticles. Increasing the internozzle distance delayed flame product mixing, increasing the crystallinity of BaCO_3_. In contrast, the single-nozzle process yielded Al_2_O_3_ particles with amorphous Ba species. The two-nozzle process enhanced NO_x_ storage behavior, while the single-nozzle approach showed negligible NO_x_ retention [59]. Following this successful novelty method, a series of Al_2_O_3_-based articles were published, herein chronologically presented: Minnermann et al. [60] produced in one nozzle Al_2_O_3_ and in the other pure oxide or mixed CoO_x_. Single flame synthesis is inadequate for producing an effective Al_2_O_3_/Co FT catalyst due to inadequate reducible cobalt oxide support particle size. The DN-FSP geometry significantly influences the resulting catalyst, yielding smaller alumina particles as the intersection distance increases, resulting in good adhesion of the two oxides and good stabilization. Høj et al. [61] produced Al_2_O_3_/CoMo by DN-FSP, and varying flame mixing distances (81–175 mm) minimized the formation of CoAl_2_O_4_, detectable only at short flame distances. Notably, employing DN-FSP synthesis achieved superior promotion of the active molybdenum sulfide phase, potentially attributed to reduced CoAl_2_O_4_ formation, consequently enhancing Co availability for promotion. Schubert et al. [62], through DN-FSP, produced Al_2_O_3_/Co enhanced with Pt (0.03, 0.43 wt%) deposition in the first nozzle and other materials in the second nozzle. Noble metals enhance catalyst reducibility, yielding abundant metallic Co sites. Due to their high cost, optimizing synthetic strategies for low concentrations is essential. Regardless of the preparation approach, adding 0.03 wt% Pt significantly improves catalytic activity in CO_2_ methanation, and 0.43 wt% Pt marginally increases the catalyst reduction. Using DN-FSP, Horlyck et al. [63] produced Al_2_O_3_/Co with Lanthanum doping (0–15 wt%). Increased La content and wider nozzle distance suppressed undesirable CoAl_2_O_4_ spinel phase, promoting easily reducible Co species. La addition enhanced carbon resistance, ensuring maximum methane conversions at 15 wt% La without catalyst deactivation or carbon formation. Stahl et al. [64] used DN-FSP to produce Co/Al_2_O_3_; in the nozzle of Al_2_O_3_, one additional particle—SmO_x_, ZrO_x_, or Pt—was formed contributing different cocatalytic effects, enhancing surface hydrogen or carbon oxide concentrations (see Figure 8a,b). All catalysts had consistent morphology with interconnected 12 nm alumina oxides and ~8 nm cobalt oxides. For CO_2_ methanation, Pt and zirconia proved optimal, aligning with Pt-enhanced H_2_ adsorption and zirconia’s higher CO_2_ adsorption due to oxide sites with medium basicity.

**TiO_2_:** Grossmann et al., through the utilization of DN-FSP, produced TiO_2_ with deposited Pt particles [67]. Geometric configurations in DN-FSP strongly influenced Pt particle size and distribution on TiO_2_. Larger intersection distances and smaller angles result in nonuniform large and broadly distributed Pt clusters on TiO_2_. Conversely, smaller distances and larger angles enhance Pt dispersion and a uniform mixing, akin to single flame; however, DN-FSP allows for individual tuning of compound particle sizes. Solakidou et al. produced {TiO_2_-Noble metal} nanohybrids, with deposition of Pt^0^, Pd^0^, Au^0^, or Ag^0^ [68]. As shown, DN-FSP is superior vs. single-nozzle-FSP for finely dispersing noble metals on TiO_2_ support, achieving a narrower size distribution [50]. DN-FSP promoted intraband states in TiO_2_/noble metal, reducing the band gap. Efficient H_2_ generation presented the following trend: Pt^0^ > Pd^0^ > Au^0^ > Ag^0^, in line with a higher Schottky barrier upon TiO_2_ contact [50]. Gäßler et al. produced SiO_2_, TiO_2_, and SiO_2_-TiO_2_ mixture with DN-FSP deposition of Co_3_O_4_ (see Figure 8c–f) [65]: titania, comprising anatase and rutile phases, the SiO_2_-TiO_2_ mixed support, with separate anatase and silica phases. H_2_O adsorption varies significantly based on the support: SiO_2_ < SiO_2_-TiO_2_ < TiO_2_. CH_4_ formation rate increased with higher TiO_2_ fractions, while CO formation rate peaked in the mixed support. Psathas et al. used DN-FSP to engineer heterojunctions of perovskite SrTiO_3_ with deposited CuO nanoparticles (0.5 to 2 wt%) [58]. Higher CuO deposition led to larger SrTiO_3_ particle sizes due to increased enthalpy from the second flame [40]. Scanning TEM depicted small CuO particles (<2 nm), mainly found on the surface of SrTiO_3_. The dopant concentration significantly controlled the selective production of H_2_ or CH_4_ from H_2_O/CH_3_OH. CuO incorporation drastically shifted production to CH_4_, achieving a rate of 1.5 mmol g^−1^ h^−1^ for the La:SrTiO_3_/CuO catalyst (0.5 wt%) [58].

**Table 3:** The literature summary of characteristics/conditions for nanostructures synthesized by symmetric and asymmetric DN-FSP methods.

Nanomaterial	Geometric Parameters (cm)	Precursor(s) *	Molarity * (mol L^−1^)	Precursor * (mL min^−1^)	Oxygen * (L min^−1^)	Size (nm)	SSA (m^2^ g^−1^)	Ref.
***Symmetric DN–FSP***								
Al_2_O_3_/Pt/Ba	φ = 30°, d = 3–7	Al(III) tri-*sec*-butoxide/ Ba(II) 2-ethylhexanoate, Pt(II) acetylacetonate	0.5/ 15.4 wt% Ba, <1 wt% Pt	5/ 3	5/ 5	10–20	120–160	[59]
Al_2_O_3_/Co	φ = 20°, d = 11, a = 16	Al-sec-butoxide/Co naphthenate	0.5/ 10 wt% Co	5/ 5	5/ 5	15–30	111–122	[63]
TiO_2_/Pt^0^, Pd^0^, Au^0^, Ag^0^	φ = 30°, d = 11	Ti(IV) isopropoxide/ Pt(II), Pd(II), Au(III), Ag(I) acetylacetonate	0.64/ 0–5 wt%	5/ 3–7	5/ 3–7	10–20	72–200	[68]
TiO_2_-SiO_2_/ Co	φ = 20° d = 15, a = 22	Ti(IV) isopropoxide, TEOS/ Co naphthenate	0.9/ 9 wt% Co	5/ 5	5/ 5	7–16	86–284	[65]
SrTiO_3_/CuO	φ = 20°, d = 8, a = 10	Sr acetate, Ti(VI) isopropoxide/ Cu(II) nitrate trihydrate	0.4/ 2–0.5 wt% Cu	5/ 5	5/ 5	45–55	32–57	[58]
ZrO_2_/CuO	φ = 10°	Zr 2-ethylhexanoate/Cu(II) 2-ethylhexanoate	0.5/ 11 wt% Cu	5/ 5	5/ 5	10–20	106–114	[69]
LiMn_2_O_4_/AlPO_4_	φ = 20°, d = 17	Li and Mn(III) acetylacetonate/Al-tri-sec-butoxide, triethyl phosphate	–/ 0–5 wt% AlPO_4_	3–7/ 5	3–7/ 5	7–22	64–195	[70]
CeO_2_:Eu^3+^/ Y_2_O_3_:Tb^3+^	φ = 30°	Ce 2-ethylhexanoate/Y nitrate hexahydrate	0.3/ 0.4	3–12/ 3–12	3–8/ 3–8	5	185	[66]
***Asymmetric DN–FSP***								
SiO_2_/Ce_0.7_ Zr_0.3_O_2_	φ1 = 20°, φ2 = 35°, a = 23, b = 5 d = 11, z = 10	TEOS/Ce 2-ethylhexanoate, Zr(IV) *n*-propoxide	0.5/ 0.19	3–7/ 5	5/ 5	18.5–28.5	217–363	[71]
NaTaO_3_/NiO–Pt^0^	φ1 = 30°, φ2 = 15°, a = 20, b = 24, d = 11, z = 3	Na 2-ethylhexanoate, Ta(V) chloride/Ni(II) 2-ethylhexanoate, Pt(II) acetylacetonate	0.1–0.6/ 0.5 wt% Ni, 0.5 wt% Pt	3–9/ 5	3–9/ 5	12–34	19–84	[72]

* In the setup, because there are two distinct nozzles, parameters related to them are differentiated and denoted using a slash: nozzle 1/nozzle 2.

**Other particles:** Tada et al., using DN-FSP, produced a ZrO_2_/CuO heterostructure [69]. Changing the geometrical parameters of DN-FSP altered the proportion of interfacial sites vs. copper surface sites. As active sites are primarily at the metal–oxide interface, ZrO_2_/CuO with smaller CuO clusters exhibited higher activity in methanol synthesis via CO_2_ hydrogenation. Gockeln et al., by a combination of DN-FSP and a lamination technique [73], synthesized in situ carbon-coated nano-Li_4_Ti_5_O_12_ Li-ion battery electrodes. Li et al. synthesized LiMn_2_O_4_ spinel as a cathode material for Li-ion batteries via screening 16 different precursor–solvent combinations [70]. To overcome the drawback of capacity fading, the deposition of AlPO_4_ (1–5%) via DN-FSP was homogeneously mixed with LiMn_2_O_4_. The optimal 1% AlPO_4_ with LiMn_2_O_4_ demonstrated an energy density of 116.1 mA h g^−1^ at 1 C (one-hour discharge). Henning et al. used DN-FSP to engineer luminescent biosensors CeO_2_:Eu^3+^/Y_2_O_3_:Tb^3+^ [66]. CeO_2_:Eu^3+^ nanoparticles (6 nm, 22 wt%) and Y_2_O_3_:Tb^3+^ nanoparticles (32.5 nm, 78 wt%) were shown to function as robust optical-based ratiometric H_2_O_2_ biosensors (see Figure 8g,h). Based on the collective effect, H_2_O_2_ caused significant luminescence quenching in CeO_2_:Eu^3+^ nanocrystals, but Y_2_O_3_:Tb^3+^ nanoparticles were unaffected [48].

**Asymmetric Double Flame:** Lovell et al. utilized asymmetric-DN-FSP geometry to control the SiO_2_ interaction with Ce_0.7_Zr_0.3_O_2_ nanoparticles [71]. Tuning the intersection distance during DN-FSP (18.5 to 28.5 cm) prevented silica coating. Short intersection distances led to high surface-area silica encapsulating ceria-zirconia, while longer distances suppressed this encapsulation. The material at longer intersection distances, used as Ni support for dry methane reforming, showed enhanced oxygen storage capacity and basicity, yielding a highly selective catalyst. Psathas et al. used asymmetrical-DN-FSP-deposited NiO or Pt^0^ nanomaterials on the surface of Ta_2_O_5_ or the perovskite NaTaO_3_ [72]. Single-step synthesis of the smallest produced NaTaO_3_ (<15 nm), with finely dispersed NiO or Pt^0^ (<3 nm). NaTaO_3_/NiO produced from FSP had half the photocatalytic hydrogen production than those from DN-FSP. Also, DN-FSP had a ten times higher yield than the conventional deposition of wet-impregnated NiO. Similar results were found for the photocatalytic efficiency of NaTaO_3_/Pt^0^, which was 30% more photocatalytically active than the conventional liquid-Pt photo-deposition method [54].

### 2.3. In Situ Coatings by FSP

**Figure 9:**
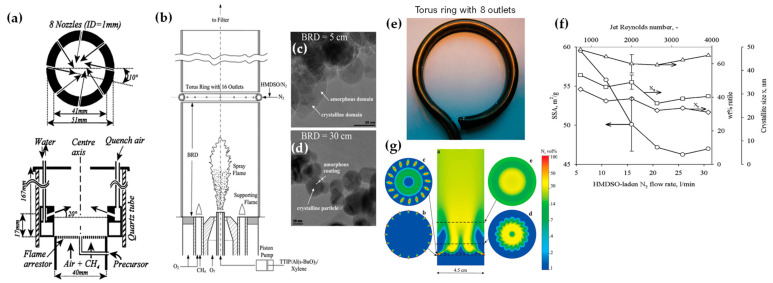
(**a**) Overhead perspective of the stainless steel segment in the quench ring according to the research work of Hansen et al. [74]; every nozzle is angled at 10° compared with a hypothetical line passing through the central axis. Reproduced with permission from ref. [74]. Copyright 2001 Wiley-VCH. (**b**) Experimental configuration for in situ SiO_2_ coating of TiO_2_ nanoparticles produced by Teleki et al. [75], using a toroidal pipe ring with 16 gas exits to inject HMDSO-laden N_2_. At burner ring distances (BRD) of (**c**) 5 cm and (**d**) 30 cm, this leads to distinct SiO_2_/Al_2_O_3_/TiO_2_ or SiO_2_-layered Al/TiO_2_ particles, each containing 4 wt% Al_2_O_3_ and 20 wt% SiO_2_, respectively. Reprinted (adapted) with permission from [75]. Copyright 2008 American Chemical Society. (**e**) Toroidal pipe ring equipped with 8 outlets for injection of the HMDSO-laden N_2_. (**f**) Impact of the ring N_2_ flow rate (along with the associated jet Reynolds number at 300 K) under standard coating conditions on SSA (depicted by circles), rutile weight percentage (represented by triangles), anatase (shown as squares), and rutile (illustrated by diamonds) crystallite sizes of 20Si-coated Al/TiO_2_. (**g**) Graphical representations of the (a) N_2_ volume percentage for 16 jets with a combined volume flow of 15.8 L/min N_2_ (v_0_ = 58 m/s). The related cross-sectional views are displayed at heights of 0 (**b**), 0.2 (**c**), 1 (**d**), and 3 cm (**e**) above the outlet level. The logarithmic color gradient extends from <1 (in blue) to 100 (in red) % *v*/*v* of N_2_. Reprinted (adapted) with permission from [77]. Copyright 2009 American Chemical Society.

Apart from heterojunction engineering, FSP allows for in situ engineering of core-shell structures, i.e., where a hermetic layer can be deposited on the core particle. Hansen et al. were the first to present this concept, using a spraying-ring apparatus, which facilitated the synthesis of ZnO particles (see Figure 9a) [74]. This research revolved around the exploration of how introducing cold air to cool a flame could influence the formation of ZnO particles. Their findings indicated that a swift drop in temperature downstream from the peak greatly benefitted the creation of particles with significant specific surface area [74]. Later on, this ring-spraying setup was predominantly employed to produce core-shell particles. Teleki et al. demonstrated that a hermetic SiO_2_-layer can be formed around TiO_2_ nanoparticle FSP reactor via injection of hexamethyldisiloxane (HMDSO) vapor—a precursor for SiO_2_—on the TiO_2_-forming stream (see Figure 9b–d) [75]. Addressing the problem of distinct Si and Ti domains found in earlier research [76], they incorporated a toroidal ring in an encapsulated FSP reactor. This facilitated the separate introduction of the gaseous Si precursor, HMDSO. The study emphasized that under specific conditions, it is possible to achieve the desired coatings in a single-phase system, avoiding the complexities of multiple phases [76]. In subsequent research, the same team (see Figure 9e–g) [77] utilized experimental and computational fluid dynamics (CFD) techniques to investigate the integrity of resultant coatings. Flame-made nanoparticles have been effectively coated in a single step, achieving notable production rates of 30 g/h. Predominantly, rutile TiO_2_ nanoparticles (enriched with Al) with an approximate diameter of 40 nm were synthesized with an in situ coating of 20 wt% SiO_2_. CFD further clarified the effects of merging the TiO_2_ aerosol with the HMDSO vapor stream jets (see Figure 9g).

**Figure 10:**
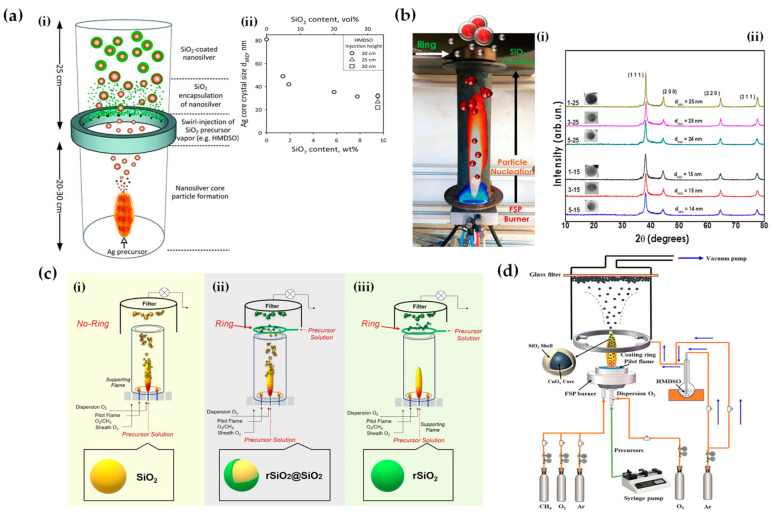
(**a**) (i) Illustration of the enclosed FSP setup for producing Ag@SiO_2_ nanoparticles, originally developed by Sotiriou et al. [78]. The ring situated between the two tubes aids in swirling the SiO_2_ precursor vapor, ensuring the precise control of the SiO_2_ core. (ii) Adjustment of the crystallite size of nanosilver can be achieved by varying the SiO_2_ content in the final nanosilver particles and by altering the injection height of the SiO_2_ precursor vapor to 30 (circles), 25 (triangles), or 20 cm (squares) above the flame spray burner. Enhancing the SiO_2_ content helps to prevent the agglomeration and crystal growth of Ag nanoparticles. Additionally, introducing HMDSO (the SiO_2_ precursor) at reduced heights cools the flame aerosol, further inhibiting Ag crystal growth. Reproduced with permission from ref. [78]. Copyright 2010 Wiley-VCH. (**b**) (i) Schematic depiction of the enclosed FSP reactor where the one-step Ag coating SiO_2_ particles occur in-flight [80]. Contrary to Sotiriou et al.’s findings [78], the metal ring was at the top of the FSP-enclosed flame. (ii) XRD patterns of SiO_2_@Ag^0^ nanoparticles with variations in size and shell thickness. Reprinted (adapted) with permission from [80]. Copyright 2019 American Chemical Society. (**c**) Schematic illustration of FSP reactor configurations: (i) designed for high-temperature nano-SiO_2_ production, (ii) tailored for the hybrid rSiO_2_@SiO_2_ nanosilica, and (iii) set up for low-temperature nano-rSiO_2_ synthesis. Reprinted (adapted) with permission from [82]. Copyright 2022 American Chemical Society. (**d**) FSP apparatus for creating core-shell CuO_x_@SiO_2_ nanoparticles. Reprinted from [85], with permission from Elsevier.

Building upon similar principles, Sotiriou et al. demonstrated the one-step production of Ag/SiO_2_ core-shell nanoparticles [78]. The innovative design of their apparatus, featuring a torus ring with multiple jets, ensured the accurate in-flight coating of particles (shown in Figure 10a). A crucial aspect of their work was to understand how the SiO_2_ content in resultant particles can be manipulated through process adjustments, influencing the size of the nanosilver particles and preventing their agglomeration. The particles were covered in-flight by injecting HMDSO through a torus ring equipped with multiple evenly spaced and uniform jets, each having a diameter of 0.6 mm. This injection occurred at a specific height referred to as the burner ring distance (BRD). Regardless of the BRD values, the length of the tube above the torus ring was maintained at 40 cm. HMDSO vapor-laden N_2_ gas at a flow rate of 0.8 L/min was obtained by bubbling nitrogen gas through liquid HMDSO at varying temperatures, through the jet openings. The amount of HMDSO injected was adjusted to achieve a targeted theoretical coating thickness (CT) of either 3 or 6 nm [79]. The SiO_2_ content in the resulting particles was determined under complete saturation conditions. Our research group extended this knowledge and developed Ag@SiO_2_ particles with a slightly modified experimental setup [80,81] illustrated in Figure 10b. The HMDSO vapor was generated by bubbling N_2_ gas through 300 cm^3^ of HMDSO contained in a glass flask, which was maintained at a temperature of 10 °C. Under saturation conditions, this configuration resulted in a theoretical SiO_2_ production rate of 5.9 g/h, equal to 20 wt% SiO_2_ in the product powder [80]. More recently, we have demonstrated that ring-coating FSP allows engineering safe-by-design core-shell SiO_2_ materials with diminished reactive oxygen species (ROS) generation [82]. As shown in [64], this stems from the flexibility of ring-coating FSP toward modulating the thermal profile during nano-SiO_2_ synthesis. A cooler SiO_2_-formation process allowed for the surface passivation of the nanosilica, consequently decreasing its ROS generation potential [64]. In this process, the N_2_ gas, which served as a carrier of the atom to be dispersed, played a pivotal role in the cooling dynamics [83]. More recent research works include the use of ring-spraying FSP for the production of stable core-shell ZnO@SiO_2_ [84], CuO_x_@SiO_2_ (see Figure 10d) [85], SiO_2_@YAlO_3_:Nd^3+^ [86], and SiO_2_-coated Y_2_O_3_:Tb^3+^ [87] along with their FSP characteristics listed in Table 4.

### 2.4. Sequential FSP Deposition

**Figure 11:**
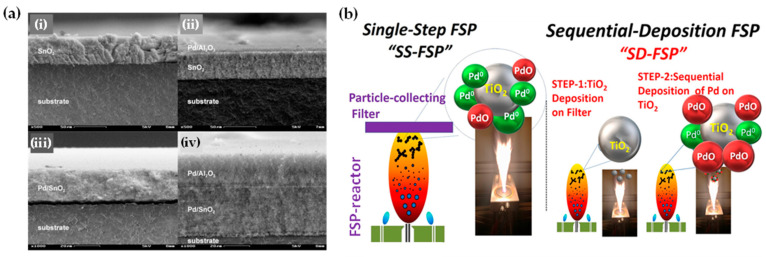
(**a**) The concept of sequential deposition technique, as originally exemplified by Sahm et al. [89], for the engineering of multilayer films. Cross-sectional SEM images of a SnO_2_ layer are shown (i); a Pd/Al_2_O_3_ layer over a SnO_2_ layer (ii); a Pd/SnO_2_ layer (iii); and a Pd/Al_2_O_3_ layer on the top of a Pd/SnO_2_ layer (iv)—all of which were deposited on ceramic bases. Reprinted from [89], with permission from Elsevier. (**b**) A schematic depiction of the FSP process employed for the deposition of Pd onto the TiO_2_ surface in two stages (sequential deposition, SD-FSP). Reprinted (adapted) with permission from [91]. Copyright 2020 American Chemical Society.

The concept of sequential deposition was originally used for the fabrication of multilayer films, sensors [89], and fuel-cell applications [41]. Mädler and his colleagues fabricated multilayer films for gas sensing where two different sensing layers were deposited on ceramic substrates sequentially: pure SnO_2_ onto Pd/SnO_2_ [90] or Pd/Al_2_O_3_ layer on top of a Pd/SnO_2_ layer (refer to Figure 11a) [89]. Recently, we have developed a sequential-deposition flame spray pyrolysis (SD-FSP) technique for the controlled synthesis of PdO/Pd^0^/TiO_2_ nano-heterostructures [91]. SD-FSP is a two-phase process in which a nanometric TiO_2_ particle layer is deposited on a glass-fiber filter in the first FSP step. Then, in a second FSP phase, Pd particles are produced in an FSP flame under conditions that permit control over the Pd NP size and PdO/Pd^0^ ratio (as shown in Figure 11b). In this SD-FSP process, combustion of the Pd precursor under open-flame conditions permits ambient O_2_ entrainment without O_2_ consumption by TiO_2_ formation during the combustion [91].

**Figure 12:**
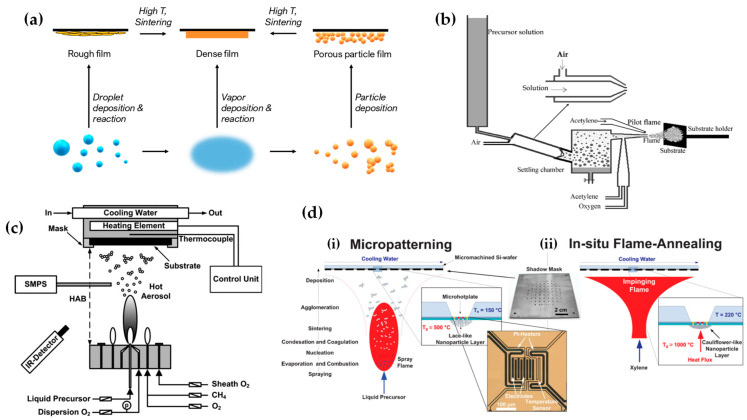
(**a**) Schematic illustration of the creation of porous or solid films through the flame deposition of droplets, vapors, or particles. (**b**) Illustrative diagram of the flame spray pyrolysis deposition setup; the embedded image displays an external mix atomizer for film deposition. Reprinted from [96], with permission from Elsevier. (**c**) FSP burner combined with a temperature-regulated substrate holder designed for nanofilm creation and deposition. Reproduced with permission from ref. [98] Copyright 2012 Wiley-VCH. (**d**) Diagrammatic representation: (i) Flame-made nanoparticle layers, exhibiting a high porosity of 98%, are methodically deposited onto a silicon wafer using a shadow mask. Subsequently, (ii) these layers undergo in situ mechanical stabilization via an impinging xylene flame devoid of particles. Reproduced with permission from ref. [99] Copyright 2008 Wiley-VCH.

**Figure 13:**
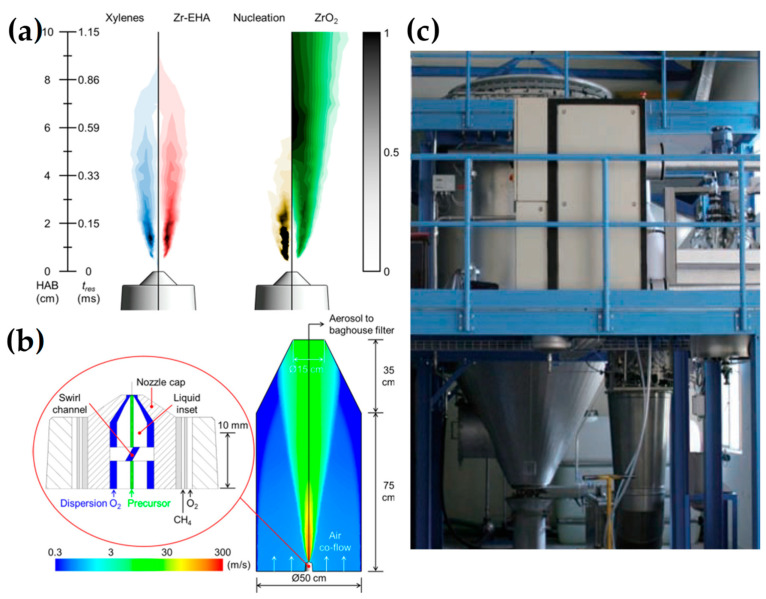
(**a**) Predicted normalized gas-phase mass fractions, the ZrO_2_ formation profile (brown), and volume concentration (green). (**b**) Schematic of FSP two-phase atomizer geometry. Reprinted (adapted) with permission from [117]. Copyright 2014 American Chemical Society. (**c**) A flame spray pyrolysis (FSP) pilot plant is designed to produce multiple kg h^−1^ of nanoparticles, incorporating a baghouse filter for nanoparticle collection with an approximate filtration area of ~50 m^2^. Reprinted from [108].

FSP has been adeptly employed for the synthesis of films characterized by a porous network comprising nanoparticles whose porosity can be modulated. Such films have exhibited superior efficacy in applications like chemical sensors [92], photodetectors [93], and solar cells [94]. Homogeneity in films still remains a challenge, especially since cracks can easily form when drying films, i.e., created through traditional wet-phase coating methods. However, films formed in the gas phase [2] do not require drying, leading to more consistent layers, e.g., as in chemical vapor deposition (CVD) methods [95]. The film’s structure resulting from FSP deposition largely relies on the substrate temperature and the stage of particle formation when reaching the substrate. Characteristically, Figure 12b illustrates a schematic of a nanofilm deposition procedure. The nature of the deposit—whether it be precursor droplets, a mix of precursor and product vapors, or product particles exhibiting various agglomeration levels—depends on the particle’s formation phase when it reaches the substrate. This characteristic can be controlled by adjusting the substrate’s position or by modulating the precursor and gas flow rates. When uncovered precursor comes into contact with the substrate, it produces denser films [28] whereas airborne product particles result in highly porous particle films, as depicted in Figure 13a. The porosity of such films can either remain intact or transform into denser formations through a sintering process, contingent on the substrate’s temperature. The nanofilm deposition procedure can be clarified in the research work of Kavitha et al. where they produced TiO_2_, ZnO [96], and Al_2_O_3_, ZnO, ZnO-20 mol% MgO, and ZrO_2_-Y_2_O_3_ [97] films. Films of the aforementioned oxides were applied onto amorphous silica bases measuring 10 mm × 10 mm. The setup for deposition, as depicted in Figure 13b, comprises a liquid sprayer, a division chamber, a flame, and a holder for the substrate. In [38], a combination of precursor solution and pressurized air (at 16 lpm) was directed into the atomizer, which generated atomized droplets of the solution and injected them into the separation chamber. Adjustments to this temperature can be made by altering either the gas flow rates or the distance between the substrate and nozzle [96]. A straightforward relationship between the process parameters and the rate of film formation were established by Tricoli et al. [98] in the case of SnO_2_ films deposited on substrates at temperatures 323 to 723 K in order to study the particle size distribution and deposition dynamics.

Incorporating flame-based aerosol techniques to industrial-scale semiconductor device engineering poses new challenges. The mechanical integrity of nanostructured layers hinges on the substrate temperature at the time of deposition. While mechanically robust layers can be achieved at elevated temperatures (850 °C), these temperatures do not align with complementary metal oxide semiconductor (CMOS) substrates that host circuit components, as they cannot withstand temperatures exceeding 400 °C. Thus, Tricoli and his colleagues introduced a strategy for stabilizing deposits thermally by using an in situ rapid flame treatment on nanoparticle micropatterns deposited at low temperatures [99]. This technique can be applied while keeping the substrate at low temperatures, and the original crystallite size remains unchanged. The synthesis of metal oxide nanoparticles occurs through the dispersion and ignition of a precursor spray solution of the target material—in [98], it was SnO_2_. Particle growth progresses through condensation, surface growth, coagulation, and sintering, producing nanocrystalline material (as seen in Figure 12d(i)). Factors like high-temperature particle residence time (HTPRT), metal concentration, and droplet dispersion during FSP influence the particle size [2]. Nanostructured particles then adhere to the substrate in situ, usually forming a highly porous layer. However, due to the substrate’s low temperature (150 °C), this layer has limited mechanical resilience. To enhance its stability, a second phase involves subjecting to thermal curing in FSP, e.g., an “impinging” step, for 30 s using a particle-free xylene flame, as depicted in Figure 12d(ii). This treatment profoundly alters the layer’s texture, enhancing its adhesion to the substrate. Patterns of nanoparticles, with sizes as small as 100 µm, were created by positioning a shadow mask, designed with a series of circular openings, against a Si-wafer (coated with a thin layer of silicon nitride), as shown in Figure 12d [99].

### 2.5. Scale-Up FSP

Several of the methods used for nanoparticle synthesis are not scalable, i.e., to transcend laboratory production toward industrial scale for large-volume production. These facts are against the actual implementation of real-life applications, hindering the connection of lab research to market-level production. When more complex nanoparticles require time-consuming synthesis protocols with complex processes, these factors lead to very high prices per kilo of particles [100].

Gas-phase synthesis has already shown much promise for the industrial production of nanoparticles. So far, single-metal nanomaterials are produced through gas-phase processes [27]. Gas-phase synthesis includes many essential commodity products that have been widely produced for many years, with some of the most widespread nanomaterials for the industry, such as carbon black by the company Cabot [101] as a reinforcing agent, P25 (TiO_2_) by Evonik Industries [102] renowned for its photocatalytic properties, pigmentary titania by the companies DuPont, Cristal, and Ishihara, fumed silica (SiO_2_) by Cabot and Evonik, as well as ceramic-based nanoparticles with the application of flame aerosol processes. The production of flame-made nanoparticles generates millions of tons with a valuation reaching $15 billion/year [38].

The successful utilization of aerosol-made nanomaterials in the market indicates that the industrial-scale manufacturing of gas phase will expand further with future applications, using more complex particles and multicomponent particles that cannot be easily produced at industrial scale with other methods [1]. Utilizing the advantages of FSP, several start-up companies have grown to produce various particles with controlled characteristics to fill the demand for niche markets [103]. Today, Hemotune AG (Schlieren, Switzerland) [104] produces polymer-decorated iron nanoparticles with carbon encapsulation for the purpose of blood purification. Examples are Anavo Medical (Zurich, Switzerland), for bioactive hybrid metal oxides, and Avantama AG (Stäfa, Switzerland), for the production of several metal oxides [103]. Such start-up companies include Turbobeads AG (Zurich, Switzerland) [105], which creates amine-functionalized cobalt carbon-coated nanoparticles. HeiQ Inc. (Schlieren, Switzerland) [106], which recently had an IPO with a valuation of 127 M£, produces nanosilver by FSP, the third most market-demanded nanomaterial, following carbon black and fumed oxides [107].

FSP synthesis can be implemented by laboratory FSP with production of 10 g h^−1^ to establish the synthesis optimization protocol and the initial exploration for complex particles and industrial-scale FSP with production rates at kg h^−1^ [108]. With lab-scale FSP, many particles reach 10 g h^−1^. Examples include HfO_2_ (5 nm, 89 SSA) at 15 g h^−1^ [109] and CeO_2_ (8 nm, 101 SSA) at 10 g h^−1^ [110]. Pratsinis et al. synthesized particles of silica/titania with a production rate of 200 g h^−1^ [111,112], showing very different results with the change in the fuel flow rate and the oxygen flow rate parameters. In this context, many simple oxides have been synthesized with FSP, such as WO_3_/TiO_2_ [113], or more complex nanoparticles, such as La_0.6_Sr_0.4_Co_0.2_Fe_0.8_O_3−δ_ with production rates as high as 400 g h^−1^ [114].

***Industrial FSP*:** In Table 5, the industrial-scale FSP publications are presented in chronological order and their synthesis/structural characteristics are listed. The first industrial-scale FSP production was demonstrated for SiO_2_, by Mueller et al., producing uniform 25 nm particles at 1.1 kg h^−1^ [115]. The study investigated the primary particle diameter, morphology, and carbon content by HMDSO in EtOH at 1.26 M and 3.0 M, as well as pure HMDSO at 4.7 M. Notably, the average primary particle size of the product was precisely controlled within the range of 10 to 75 nm, irrespective of the precursor concentration. Additionally, it was observed that utilizing air instead of O_2_ as the dispersion gas resulted in minimal variation in the product particle size. Mueller et al. achieved ZrO_2_ with a production rate of 0.6 kg h^−1^ with an average size of 30 nm [116]. The study evaluated zirconium *n*-propoxide in EtOH at 0.5 M and 1 M concentrations. Primary particle size ranged from 6 to 35 nm, with the crystal structure mainly tetragonal (80–95 wt%). The primary particles showed weak agglomeration, forming loosely agglomerated single crystals. Gröhn et al. produced ZrO_2_ at 0.5 kg h^−1^ [117] with increased technological expertise by a three-dimensional computational fluid dynamics model showing the fundamentals of the high-temperature particle residence time (HTPRT) for the scale-up synthesis of nanomaterials (see Figure 13a,b). HTPRT effectively regulated primary particle and agglomerate size, morphology, and ZrO_2_ crystallinity. Maintaining a constant HTPRT while scaling up the production rate from ∼100 to 500 g h^−1^ showed no significant alteration in product particle properties. In this context, Meierhofer et al. explored CFD-PBM modeling of ZrO_2_ FSP synthesis [118] at high productions to discover the attributes of the resulting nanoparticles. Jossen et al. produced yttria-stabilized zirconia (Y_2_O_3_/ZrO_2_) with the same parameters, with only the molarity dropped by half, decreasing the production rate at 0.35 kg h^−1^ [119]. Homogeneous Y_2_O_3_/ZrO_2_ exhibited an average crystallite and particle diameter ranging from 8 to 31 nm, with yttria content varying between 3 and 10 mol%. Interestingly, the yttria content had no discernible impact on the primary particle and crystal sizes.

**Table 5:** Industrial production of nanoparticles by FSP, parameters, production rate, size, and SSA.

Nanomaterial	Production Rate (kg h^−1^)	Precursors	Solvents	Molarity (mol L^−1^)	Precursor (mL min^−1^)	Oxygen (L min^−1^)	Size (nm)	SSA (m^2^ g^−1^)	Ref.
**SiO_2_**	1.1	HMDSO	Ethanol	4.7	33.3	50	26	108	[115]
**ZrO_2_**	0.6	Zr n-propoxide	Ethanol	1	81	50	30	33	[116]
**ZrO_2_**	0.5	Zr 2-thylhexanoate	Xylene	1	64	80	25	42	[117]
**Y_2_O_3_/ZrO_2_**	0.35	Zr n-propoxide/Y nitrate hydrate	Ethanol	0.5	81	50	31	32	[119]
**FePO_4_**	0.27	Fe nitrate/tributyl phosphate	2-ethylhexanoic acid	–	20	40	129	108	[108]
**ZnO**	3	Zinc nitrate hexahydrate	Ethanol, methanol, 1-propanol, 1-octanol	3.3	200	120	30	26.2	[120]
**Ca_2_SiO_4_** **(Belite)**	0.03	TEOS/calcium propionate	Ethanol, methanol, deionized water	1.1	30	30	54	34	[121]

Wegner et al. produced FePO_4_ nanoparticles with 0.27 kg h^−1^ with a mean size of 129 nm (see Figure 13c) [108]. Additionally, a cost analysis for the scale-up FSP was calculated with the pilot plant production costs for simple oxides projected to be less than 100 EUR per kilogram. Among the cost components, raw materials constitute the most significant cost factor in this estimation.

Hembram et al. manufactured ZnO nanorods with the highest production rate so far, at 3 kg h^−1^. The resulting nanorods had a length of 30 nm and a mean aspect ratio of the rods at 2.3 [120]. Numerous nanorods self-aligned by creating junctions at the basal planes and some were further assembled into tetrapods. The nanorod aspect ratio was controllable through adjustments in the concentration of Zn ions in the initial precursor solution, its delivery rate, and the oxygen flow into the reactor. Betancur-Granados et al., with a production of belite, achieved in scale-up conditions with a production of 0.03 kg h^−1^—the low production is due to the formation of amorphous and other derivatives, such as alite, CaCO_3_, etc. [121]. The hydraulic samples exhibit a full hydration reaction within a 24 h duration upon contact with water. This substantiates the technique’s potential in developing highly reactive materials suitable for prospective sustainable construction practices.

## 3. Complex Functional FSP Nanostructures and Nanodevices

The innovative exploitation of FSP’s potential enables the engineering of unconventional materials, such as non-oxides and complex structures or nanodevices, including perovskites, nanosensors, and fuel-cell-materials.

### 3.1. Engineering of Perovskites by FSP

**Figure 14:**
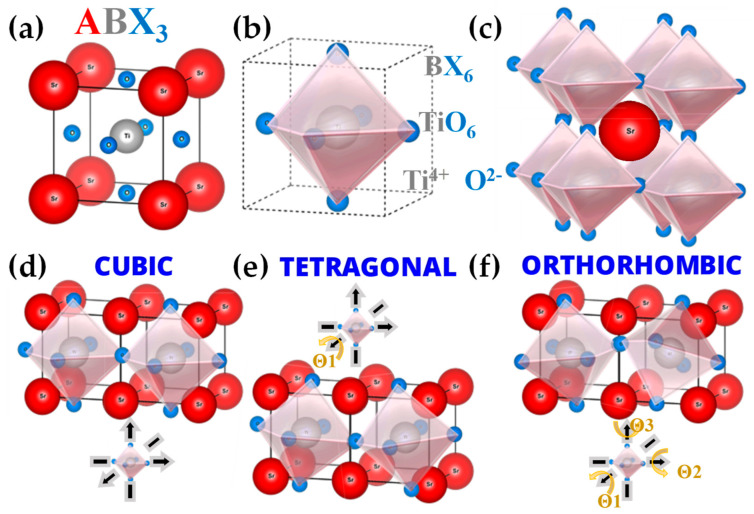
The ideal cubic perovskite structure exemplified by SrTiO_3_. (**a**) The cubic structure with the Ti^4+^ at the cell center. (**b**) The octahedral polyhedron structure TiO_6_. (**c**) Sr^2+^ at the cubic center with the octahedral structure surrounding the strontium. Different perovskite structures: (**d**) the ideal cubic perovskite, axis X, Y, and Z of the octahedral BX_6_ have a 180-degree separation. (**e**) Tetragonal perovskite structure, the octahedral BX_6_ tilted in only one of the three axes at an angle Θ1. (**f**) The orthorhombic structure, the octahedral, is titled in all three of the axes, in accordance with the angles Θ1, Θ2, and Θ3.

**Figure 15:**
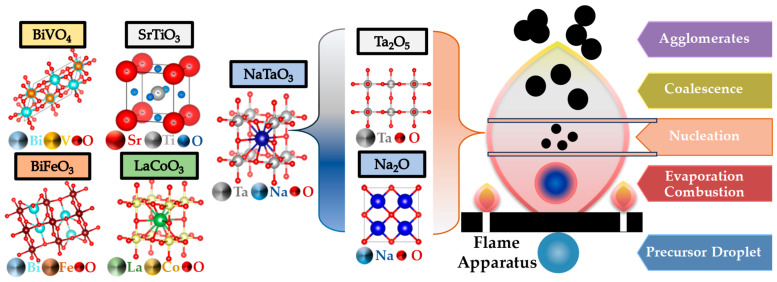
FSP formation of perovskite structure ABO_3_ requires avoidance of the formation of the two separate oxides (A-oxide, B-oxide).

FSP has been established mainly for its ability to engineer a wide range of single-metal oxides [2]. However, establishing FSP process parameters for producing ABO_3_ perovskite nanomaterials (see Figure 14a–f and Figure 15) is typically more challenging, e.g., avoiding the formation of the separate oxides AO_n_ and BO_m_.

**Figure 16:**
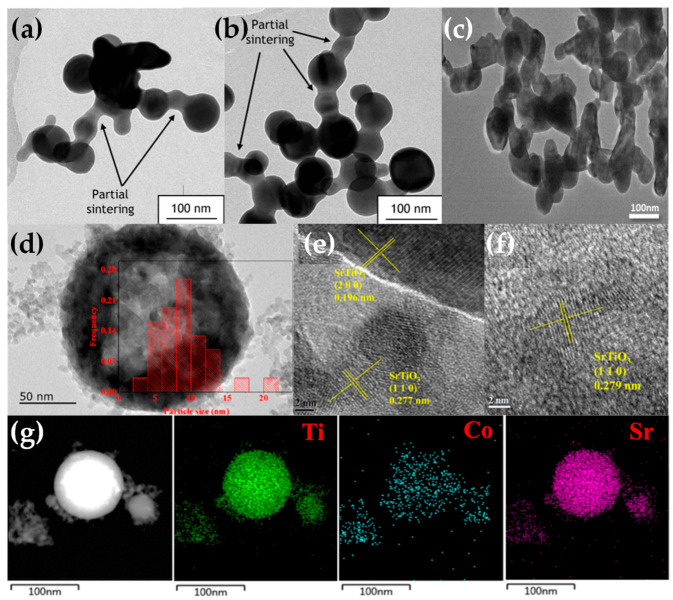
(**a**,**b**) TEM images illustrating necked particles of BiVO_4_. Reprinted (adapted) with permission from [122]. Copyright 2011 American Chemical Society. (**c**) TEM images of necked-sintered BiFeO_3_ particles. Reprinted from [124], with permission from Elsevier. (**d**–**f**) HRTEM micrographs of SrTi_1−x_Co_x_O_3_ and the miller planes. (**g**) EDX element mapping of SrTi_1−x_Co_x_O_3_ for the Ti, Co, and Sr atoms. Reprinted (adapted) with permission from [129]. Copyright 2021 American Chemical Society.

The publications regarding perovskite synthesis by FSP (see Figure 15), listed in Table 6, are rather limited in number so far. The first publication was by Kho et al. who demonstrated the synthesis of BiVO_4_ by FSP [122], where they noticed a bottleneck disadvantage of crucial importance for FSP perovskite formation—that is the short HTPRT, in the order of milliseconds, was inadequate to induce the perovskite phase (see Figure 16a,b). In situ thermal curing, on the filter, was necessary to achieve the desired scheelite BiVO_4_ phase [122]. The required filter temperature was T > 310 °C up to 360 °C to reach 100% crystallization, resulting in the scheelite-monoclinic phase, in agreement with the Tammann temperature of BiVO_4_ [109], which occurred at 300 °C. Stathi et al. [123] demonstrated that FSP can engineer W- and Zr-Doped BiVO_4_ in tandem with control of the BiVO_4_ lattice oxygen vacancies (V_O_). W-doping had a minor change on the BiVO_4_ XRD peaks, although W-doping (10%) caused a major deterioration of the crystallinity. Zr-doping above 1% showed peaks attributed to cubic-ZrO_2_ particles of 3 nm diameter. Function-wise, the presence of oxygen vacancies in W-BiVO_4_ and Zr-BiVO_4_ drastically improved the O_2_ production efficiency [123].

Psathas et al. [124,125], in their study of the engineering of perovskite BiFeO_3_ by FSP, concluded that the short HTPRS did not allow for the in situ formation of crystalline BiFeO_3_. They observed that a very short post-calcination of 5 min at 550 °C allowed for the formation of pure phase BiFeO_3_. This shows that the FSP-made BiFeO_3_ material consisted of small nanometric crystallites that were organized in larger BiFeO_3_ crystalline particles upon a small thermal/calcination boost [124,125]. Additionally, via the same protocol, mullite-type Bi_2_Fe_4_O_9_ was synthesized (see Figure 16c). These BFO materials were employed for the reduction of 4-Nitrophenol to 4-Aminophenol [124] and showed a low-activation energy E_a_ = 22 kJ mol^−1^ for BiFeO_3_, which is comparable to that of noble metal-based catalysts. Highly efficient photocatalytic O_2_ evolution, as shown in [125], was promoted via the introduction of Fe^2+^ centers in BiFeO_3_ via a downshift of the CB and VB edges. FSP-made Bi_2_Fe_4_O_9_ exemplified a highly efficient O_2_ evolution photocatalyst for the first time [125].

Punginsang et al. [126] reported the engineering of layered perovskite oxide Bi_2_WO_6_ consisting of FSP-made orthorhombic phase spherical Bi_2_WO_6_ nanoparticles (3–30 nm in diameter) and a very high specific surface area of 197.8 m^2^g^−1^. The high thermal stability FSP-Bi_2_WO_6_ nanoparticles were applied for gas-sensing measurements and displayed a stable and selective response of 3.72–2000 ppm toward acetone at 350 °C and good selectivity against other gases, surpassing similar materials made by other methods.

Recently, Xiao et al. [127] reported a successful FSP synthesis of LaCo(Fe-dopped) O_3_. In [127], a LaCo_1−x_Fe_x_O_3_-based sensing was evaluated as an ammonia sensor, reaching a detection limit of 2 ppm at 475 °C. These effects were corroborated based on the oxygen vacancy and improvement in electrocatalytic performance by doping iron. Moreover, the sensor exhibited good selectivity against other gases and good stability against oxygen and water vapor concentration fluctuation, with long-term stability for 22 days [127].

Perovskite La_1−x_FeO_3−δ_ sensors (x = 0, 0.02, 0.05, 0.07, and 0.1), with A-site deficiency, were successfully synthesized by FSP [128], with pure orthorhombic phase and size below 10 nm size. The materials were applied for chemo-resistive CO_2_ sensors, La_0.95_FeO_3−δ_ was the optimal stoichiometric material for 5–15% CO_2_ at 425 °C. Due to surface oxygen vacancies attributed to the small amount of Fe^4+^, this A-site deficiency might be responsible for enhanced sensing performance [128].

**Table 6:** The literature summary of characteristics/conditions for FSP-made perovskite-type nanomaterials.

Nanomaterial	Precursors	Solvents	Molarity (mol L^−1^)	Precursor (mL min^−1^)	Oxygen (L min^−1^)	Size (nm)	SSA (m^2^ g^−1^)	Ref.
BiVO_4_	Bi acetate/V oxytripropoxide	2-ethylhexanoic acid/xylene	0.25 (Bi)/ 0.25 (V)	10	5	62–71	7–12	[122]
W-, Zr-BiVO_4_	Bi(III) nitrate pentahydrate/V(V) oxytripropoxide	Ethoxy triglycol, acetic acid (70/30 *v*/*v*)/xylene (Total: 50/50 *v*/*v*)	0.5 (Bi)/ 0.5 (V)	5	5	15–22	38–49	[123]
BiFeO_3_	Bi(III) acetate/ Fe(III) acetylacetonate	2-Ethylhexanoic acid/xylene (50/50 *v*/*v*)	0.1 (Bi)/ 0.1 (Fe)	3	7	48–67	11–14	[125]
Bi_2_WO_6_	Bi(III) nitrate pentahydrate/ W(VI) ethoxide	Ethanol, acetic acid (70/30 *v*/*v*)	–	5	5	198	3.3	[126]
LaCoO_3_	Co(II) nitrate hexahydrate/Fe(III) nitrate nonahydrate	Methanol	0.3 (La)/ 0.3 (Co)	–	5	10	–	[127]
LaFeO_3_	La nitrate hexahydrate/Fe(III) nitrate nonahydrate	Ethanol	–	–	5	10	–	[128]
SrTi_1−x_B_x_O_3_	Sr acetate/ tetrabutyl titanate	Acetic acid/ethanol (50/50 *v*/*v*)	0.075 (Sr)/ 0.075 (Ti)/	3	5	19–37	31–46	[129,130,131]
SrTiO_3_/CuO	Sr acetate/Ti(VI) iso-propoxide	Acetic acid/xylene (50/50 *v*/*v*)	0.2 (Sr)/ 0.2 (Ti)/	5	5	45–55	32–57	[58]
NaTaO_3_/ NiO–Pt^0^	Na 2-ethylhexanoate/ Ta(V) chloride	Ethanol	0.05–0.3 (Na)/ 0.05–0.3 (Ta)	3–9	3–9	12–34	19–84	[72,132]

Recently use of FSP to engineer SrTiO_3_ perovskite (see Figure 14a–f) was established by Yuan et al. [129,130,131] and Psathas et al. [58]. In [129], SrTi_1−x_B_x_O_3_ (B = Co, Fe, Mn, Ni, and Cu) were produced by FSP at different concentrations (10%, 30%, 50% for Co, 10% for everything else). Co cation was shown to be evenly dispersed in the SrTiO_3_ structure based on EDX mapping, with an increase in dopant and the growing diameter of active Co particles aligning with TEM findings (see Figure 16d–g). As the cobalt concentration increased from 10 to 50 mol%, the size of Co particles progressively expanded, consequently reducing metal dispersion from 6.50% to 2.18%. Substituting B-site in SrTiO_3_ with varied valence metal cations created vacancies, enhancing CO/CH_4_ oxidation, with SrTi_0.5_Co_0.5_O_3_ exhibiting the highest activity [129]. In [130], SrTi_1−x_Cu_x_O_3_ particles were reported, with different copper concentrations (10%, 15%, 30%), with the copper species either as amorphous species or/and highly dispersed particles below 5 nm, with XRD indicating that copper ions replaced the Ti^4+^ in the perovskite lattice. Catalytic low-temperature CO oxidation and CH_4_ combustion at high temperatures were exemplified [130]. In [131], Sr_1−x_Na_x_Ti_1−y_B_y_O_3_ (B = Co, Mn) with x = 0, 0.1 and y = 0, 0.3, 0.5 were produced by FSP [131]. Doping the perovskite with Na and Co resulted in notably larger SSA from 43 to 65 m^2^ g^−1^ compared with cobalt doping alone. Sr_0.9_Na_0.1_Ti_0.5_Co_0.5_O_3_ demonstrated superior low-temperature reducibility, higher adsorbed oxygen ratio, and well-dispersed Co elements, resulting in outstanding thermodynamic and kinetic activity for formaldehyde oxidation [131].

Recently, Psathas et al. presented the synthesis of La:SrTiO_3_/CuO particles by DN-FSP, in tandem with La doping [58]. EDX showed that La doping was homogeneous throughout the SrTiO_3_ matrix, with no secondary phases formed, such as La_2_O_3_. Additionally, a pink-hue color is observed in comparison to the white SrTiO_3_, evidencing a change in the band gap of the SrTiO_3_. Interestingly, it was found that La doping significantly increased the SSA and pore volume of La:SrTiO_3_, from 32 to 53 m^2^/gr, and more significantly, produced a 300% increase in pore volume to 0.39 cm^3^ g^−1^. 

As discussed in [58], this is a result of the FSP process where the La doping decreases the packing/aggregation of the SrTiO_3_. La doping consistently boosted SrTiO_3_ photocatalytic activity, with 0.9% La doping exhibiting a five-fold increase in H_2_ production to 12 mmol g^−1^ h^−1^ compared with 3 mmol g^−1^ h^−1^ for pristine SrTiO_3_ [58].

Small-size NaTaO_3_ was achieved by FSP recently [72,132]. As discussed in [72,132] the inherent need for high-temperature FSP, i.e., HTPRT, required for achieving the ABO_3_-perovskite formation, poses the challenge of retaining low particle size. As shown in [72], this can be achieved by FSP via diligent control of the temperature profile in the FSP reactor. In this way, highly crystalline small NaTaO_3_ particles < 15 nm were engineered in one step, outperforming conventional synthesis methods where the previous best size reached 25 nm and typical sizes range around 100 nm [72]. An electron paramagnetic resonance study [132] of these ultrasmall NaTaO_3_ particles, 12 nm, revealed the significant influence of the nanosize on the life time of photoinduced {h^+^/e^−^} pairs. As the NaTaO_3_ particle size increases, the recombination rate increases and this holds the key to unraveling the fundamental photocatalytic capabilities, e.g., in H_2_ production [72] of such nanomaterials [132].

### 3.2. Engineering of Metal Non-Oxides by FSP

The patent of Grass et al. in 2007 marked a pivotal milestone in reducing flame spray pyrolysis (R-FSP) [44]. Since then, FSP technology has undergone a continuous advancement, exploring the engineering of “metal non-oxides” notably encompassing metal sulfides, carbides, halides, phosphates, and carbonates listed in Table 7.

To the utmost extent of our current knowledge, no investigation in regard to the synthesis of nitrides through the FSP methodology has been documented up to the present date. Nitride synthesis via FSP entails a multitude of intricate factors, encompassing nitrogen reactivity, gas chemistry, temperature control, precursor material selection, and prudent safety considerations, collectively rendering it a formidable and demanding process. Kennedy and his colleagues employed the ceramic crucible method to synthesize gallium zinc oxynitrides (Ga_x_Zn_1−x_O_y_N_1−y_), with the objective of potentially producing these oxynitrides using the FSP method in forthcoming endeavors [133]. However, a limited body of research exists wherein, in lieu of nitrides, researchers have generated nitrogen (N)-doped titanium dioxide (TiO_2_) using single-step FSP technology [134,135,136,137,138,139]. Huo et al. [134] and Bi et al. [135] exhibited a novel approach to the synthesis of N-doped TiO_2_, utilizing a modified FSP reactor. In this method, a spray nozzle was employed to introduce ammonia water, which subsequently underwent vaporization to form H_2_O vapor and NH_3_ gas reacting with the TiO_2_ nanoparticles. Other investigators [136,137,138,139] successfully attained N-doped TiO_2_ through a straightforward FSP modification, involving the addition of dilute nitric acid to the precursor during the synthesis process, coupled with the introduction of a secondary nitrogen source, specifically urea.

**Figure 17:**
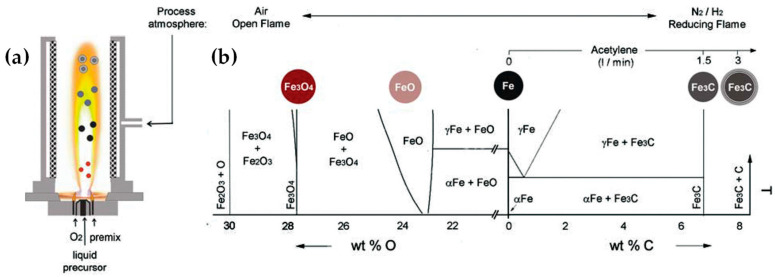
(**a**) Reducing FSP configuration for engineering Fe_3_C or C/Fe_3_C nanostructures. (**b**) Controlling the process atmosphere in flame spray synthesis enables the production of diverse iron-based nanoparticles. The resultant composition aligns with the relevant phase diagrams for Fe/O (left side) under oxidizing conditions. Subsequent reduction processes yield iron and iron carbide nanoparticles in accordance with the predictions derived from the Fe/C phase diagram (right side). Reprinted (adapted) with permission from [140]. Copyright 2009, American Chemical Society.

In the aim of carbide synthesis, Herrmann et al. [140] reported the engineering of carbon-encapsulated iron carbide (C/Fe_3_C) via an R-FSP process. In this case, the spray nozzle was placed in a glove box with a nitrogen atmosphere, which was continuously purged with nitrogen; surrounding the flame allowed for radial inflow of N_2_ at a flow rate of 25 L min^−1^ and with a carbon content varying between 1.8 and 8.05 wt%, depending on the acetylene flow rate. Iron exhibits a highly intricate Fe/C phase diagram, featuring numerous intermetallic carbon–iron phases that yield a range of technically valuable metals and steel alloys. By methodically modifying the composition of the flame feed, the authors in [140] were able to monitor the successive reduction of iron oxide to iron and iron carbides (refer to Figure 17).

**Figure 18:**
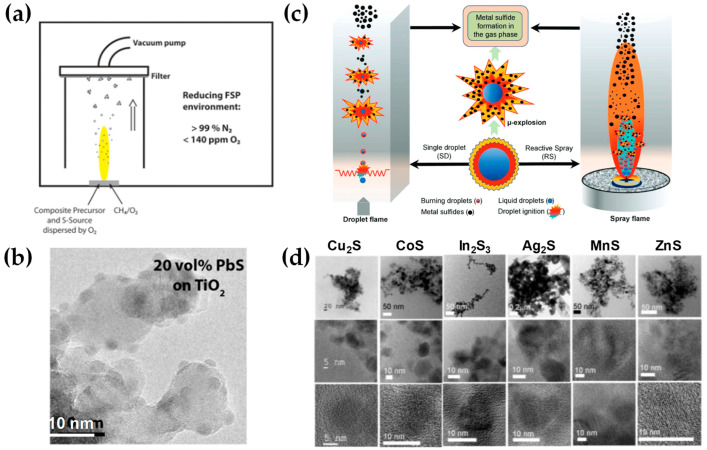
(**a**) In a one-step process, a liquid precursor containing lead, titanium, and sulfur is transformed into composite nanoparticles using a high-temperature flame reactor. Precise control of oxygen content enables fine-tuning of the system’s chemistry, facilitating the selective formation of titania support particles (oxides) and lead sulfide quantum dots (sulfides). (**b**) TEM analysis of a PbS−TiO_2_ heterojunction. The light contrast TiO_2_ nanoparticles serve as a support for PbS (darker contrast). Reprinted (adapted) with permission from [142]. Copyright 2012 American Chemical Society. (**c**) A schematic representation is presented depicting the operation of enclosed single-droplet (SD) combustion and enclosed reactive spray (RS) flame reactors for the production of metal sulfide particles. Emphasis is placed on elucidating the involvement of micro-explosions in the gas-to-particle pathway, observed in both SD and RS configurations. (**d**) From top to bottom in the column, the images depict the following: an overview of the particles, a high-resolution image of the particles, and a representative single crystalline particle—each of Cu_2_S, CoS, In_2_S_3_, Ag_2_S, MnS, and ZnS. Reproduced with permission from ref. [143]. Copyright 2023 Wiley-VCH.

Employing contemporary state-of-the-art methodologies, metal sulfides are typically acquired through diverse chemical processes, including thermolysis, solid-state metathesis, liquid-phase reactions, and thermal decomposition [141]. With respect to flame-made sulfides, a limited number of investigations have been reported in the scientific literature [50,142,143]. Originally, to facilitate the controlled combustion of a ZnS precursor within an environment characterized by oxygen-poor conditions (O_2_ < 250 ppm), the flame spray nozzle was positioned within a glove box and supplied with nitrogen to establish a reducing atmosphere [50]. Specifically, in [50], Athanassiou et al. mixed ZnO aerosol stream with in situ H_2_S, which the latter obtained via reductive decomposition of tetrahydrothiophene (THT). Additionally, reducing FSP synthesis was used to investigate the manufacturing of sulfide–oxide PbS−TiO_2_ heterojunction nanoparticles [142]. Similarly, these investigations were conducted within an enclosed box under a N_2_ purging environment (as shown in Figure 18a,b). Recently, Pokhrel et al. [143] achieved a significant advancement in this front regarding metal-sulfide nanoparticle synthesis with FSP. As a proof-of-principle [131], MnS, CoS, Cu_2_S, ZnS, Ag_2_S, In_2_S_3_, SnS, and Bi_2_S_3_ are synthesized in an O_2_-lean and sulfur-rich environment, specifically with metal:sulfur ratios ranging from 1:20 to 1:45 and average primary particle sizes in the range of 10–30 nm (refer to Figure 18d). Explicitly, the researchers mixed all the metal-organic precursors (M = Mn, Co, Cu, Zn, Ag, In, Sn, Bi) with THT directly utilizing enclosed single-droplet (SD) combustion and enclosed reactive spray (RS) FSP configurations (see Figure 18a) [143]. This protocol entailed the need for extreme N_2_ co-flow, i.e., up to 210 L min^−1^ of N_2_ supplied through a quenching ring.

**Figure 19:**
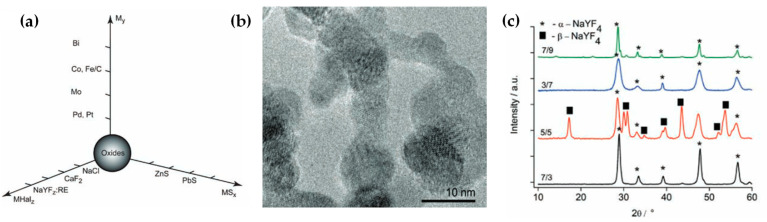
(**a**) A diagrammatic representation of the diverse nanoscale structures that can be engineered by the FSP method. (**b**) TEM depicts the hexagonal FSP-made NaYF_4_:Yb,Tm nanostructures. (**c**) XRD data of NaYF_4_:Yb,Tm nanoparticles obtained under varying fuel/oxygen flow ratios. Reprinted from [145].

In the context of FSP-made halides, Grass and Stark [144] explored FSP for the production of non-oxidic salts, including chlorides in the form of NaCl and fluorides, namely, CaF_2_, SrF_2_, BaF_2_, and Ho-BaF_2_, using carboxylate precursors and a suitable halide anion source [144]. TEM data showed that the FSP-made BaF_2_ exhibited well-defined cubic crystallites, whereas SrF_2_ and CaF_2_ exhibited slightly irregular spheroidal nanoparticles characterized by dimensions within the range of 10–40 nm [144]. Later, Stepuk et al. [145] reported the synthesis of NaYF_4_, a sodium yttrium fluoride compound, using a reducing FSP setup (see Figure 19a–c). NaYF_4_ was doped with rare-earth elements Yb, Tm, or Er for photonic-upconversion applications [136]. The FSP process involved the combustion of precursors within a nitrogen-rich atmosphere (O_2_ < 100 ppm), with a flow rate of 230 L per hour. TEM analysis evidenced the formation of spherical nanoparticles with sizes 20 to 40 nm (see Figure 19b,c). NaYF_4_ displayed the capability to undergo a phase transition from cubic to hexagonal upon thermal treatment. The co-doping of Yb-Tm and Yb-Er ion pairs resulted in the generation of blue and green upconversion luminescence, respectively. The aforementioned phosphors are characterized in terms of emission capability, phase purity, and thermal phase evolution [145].

**Table 7:** The literature summary of FSP characteristics/conditions for multifunctional non-oxide nanostructures.

Nano- Structure	FSP Configuration	Precursor(s)	Solvent(s)	Molarity (mol L^−1^)	Pilot Flame O_2_/CH_4_ (L min^−1^)	Precursor Flow (mL min^−1^)	Oxygen Flow (L min^−1^)	Reducing N_2_ Flow (L min^−1^)	Size (nm)	SSA (m^2^ g^−1^)	Ref.
***Sulfides***											
ZnS:Mn^2+^	Glove box with N_2_ atmosphere, two adsorption columns	Zn 2-ethylhexanoate, Mn naphthenate	Tetrahydrofurane, tetrahydrothiophene	0.25 [S:Zn = 5:1, Mn:Zn = 1/100 at.%]	2.2/1.2	6	5	O_2_ < 250 ppm	23	33	[50]
PbS–TiO_2_	Enclosed box under N_2_ purge	Pb(II) 2-ethylhexanoate, Ti(IV) isopropoxide, thiophene	Ethylhexanoic acid, THF (2:1)	S/Pb = 2.5	2.4/1.13	5	4–4.5	>99% N_2_, O_2_ < 140 ppm	PbS/TiO_2_ = 2/10	75–85	[142]
MnS	Enclosed reactive spray (large tube) reactor, N_2_ co-flow through porous ring, additional N_2_ flow via quenching ring	Mn naphthenate	Tetrahydrothiophene	0.25 [Mn:S = 1:34]	2.2/1.2	3	2.46	250	13.4	~62	[143]
CoS		Co naphthenate	Tetrahydrothiophene	0.25 [Co:S = 1:34]	2.2/1.2	3	2.46	250	13.4	~10	[143]
Cu_2_S		Cu naphthenate or Cu 2-ethylhexanoate	Tetrahydrothiophene	0.25 [Cu:S = 1:20, 1:41]	2.2/1.2	3	2.43–5.61	250	20.5	~46	[143]
ZnS		Zn naphthenate	Tetrahydrothiophene	0.25 [Zn:S = 1:43]	2.2/1.2	3	2.36	250	5.0	~64	[143]
Ag_2_S		Ag 2-ethoxyhexaonate	Tetrahydrothiophene	0.25 [Ag:S = 1:45]	2.2/1.2	3	2.30	250	11.6		[143]
In_2_S_3_		In acetylacetonate	Tetrahydrothiophene	0.25 [In:S = 1:43]	2.2/1.2	3	2.35	250	11.6	~103	[143]
SnS		Sn 2-ethylhexanoate	Tetrahydrothiophene	0.25 [Sn:S = 1:42]	2.2/1.2	3	2.41, 4.83	250	11.2	~106	[143]
Bi_2_S_3_		Bi neodecanoate	Tetrahydrothiophene	0.25 [Bi:S = 1:35]	2.2/1.2	3	2.45	250	20.2	~28	[143]
***Carbides***											
Fe_3_C	Glove box with radial N_2_ atmosphere, optional acetylene flow	Carbonyl iron Fe +2-ethylhexanoic acid heating at 140 °C for 24 h→Fe(III)-2-ethylhexanoate	Tetrahydrofuran	0.5	2.2/1.2	6	5	25	30	30.5	[140]
***Halides***											
CaF_2_	Open-flame FSP reactor, sheath gas O_2_ (230 L h^−1^) through a con-centric sinter metal ring	Ca hydroxide, hexafluorobenzene	2-ethylhexanoic acid, xylene		2.4/1.13	5	5	–	14	139	[144]
SrF_2_		Sr acetate, hexafluorobenzene	2-ethylhexanoic acid, xylene		2.4/1.13	5	5	–	17	84	[144]
BaF_2_		Ba acetate, hexafluorobenzene	2-ethylhexanoic acid, xylene		2.4/1.13	5	5	–	34	36	[144]
Ho–BaF_2_		Ba acetate, ho oxide, hexafluorobenzene	2-ethylhexanoic acid, xylene		2.4/1.13	5	5	–	26	48	[144]
NaCl		Sodium hydrogencarbonate, chlorobenzene	2-ethylhexanoic acid, xylene		2.4/1.13	5	5	–	92	30	[144]
NaYF_4_:Yb (Tm, Er)	Glove box with N_2_ gas flow	Tm acetate, Y acetate, Yb acetate, Er acetate, sodium bicarbonate	2-ethylhexanoic acid, xylene			3, 5, 7, 9	7, 5, 3	O_2_ < 100 ppm	20–40	29.5–31	[145]
***Phosphates***											
VOPO_4_	The solution was stirred at 60 °C and aged for 1 day	Ammonium vanadate, ammonium dihydrogen phosphate, sucrose	Deionized water, dimethyl-formamide	0.1	3/1	5	5	–	21.7–26.3		[146]
FePO_4_	Open-flame FSP reactor	Fe(III)-acetylacetonate, tri-butylphosphate	Xylene	0.2	2.4/1.13	3–7	3–8	–	30.5, 10.7	68.6, 194.7	[147]
FePO_4_	Open-flame FSP reactor	Fe(III) nitrate nonahydrate	Denaturized absolute ethanol, tributyl phosphate, 2-ethylhexanoic acid	0.2	2.5/1.25 or 2/3–9	2–6	6	–	10–20	104	[148]
Carbon-coated LiFePO_4_	Enclosed FSP with quartz tubes, C_2_H_2_ and N_2_ gas flows, sheath O_2_/N_2_ mixture (19 L min^−1^)	Li-acetylacetonate, Fe(III) acetylacetonate, tributyl phosphate	2-ethylhexanoic acid, toluene, diethylene glycol monobutyl ether, ethanol	0.24 [Li:Fe:P = 1:1:1]	2.5/1.25	3	3	20 L min^−1^ N_2_, 1 L min^−1^ C_2_H_2_	58	30	[149]
LiTi_2_(PO_4_)_3_	Open-flame FSP reactor	Li t-butoxide, Al tri-sec-butoxide, Ti isopropoxide, trimethyl phosphate	2-methoxyethanol	2.8	5/5.2	12.5	20	–	< 50		[150]
LiGe_2_(PO_4_)_3_	Open-flame FSP reactor	Li t-butoxide, Al tri-sec-butoxide, Ge ethoxide, trimethyl phosphate	2-methoxyethanol	3.2	5/5.2	12.5	20	–	< 50		[150]
Biphasic Ca_3_(PO_4_)_2_	Ultrasonic droplet generator, quartz reactor	Ca Nitrate Tetrahydrate, Diammonium hydrogen phosphate	Ethyl alcohol, distilled water (0.6:0.4)	0.4	C_3_H_8_ + O_2_ (5 L min^−1^)	5	40	–	32–38		[151]
CaP	Open-flame FSP reactor	Ca acetate hydrate, tributyl phosphate	Propionic acid	0.4	2/2	2.4	9	–	23	40–50	[152]
CaP:Eu (5 at%)	Open-flame FSP reactor	Ca acetate hydrate, Eu nitrate hexahydrate, tributyl phosphate	2-ethylhexanoic acid, propionic acid	0.2, 0.1	3.2/1.5	8, 3	3, 8	–	26, 8	73, 246	[153]
***Carbonates***											
CaCO_3_	Open-flame FSP reactor	Ca 2-ethylhexanoate	2-ethylhexanoic acid, xylene	0.4	3–7		3–7	–	20–50	31.1–102.6	[154]
BaCO_3_	Open-flame FSP reactor	Ba(II) 2-ethylhexanoate	Ethanol	0.2		5	5	–	50–100	20.5	[155]
***Pure metals***											
Bi	Glove box with radial N_2_ flow, two adsorption columns	Bi(III) 2-ethylhexanoate	Tetrahydrofuran		2.2/1.2	2–6	2.5–5	25 (O_2_ < 100 ppm)	51–127		[46]
C/Cu	Glove box with N_2_ flow	Cu(II)-2- ethylhexanoate	Tetrahydrofuran		2.2/1.2	4.5	5	N_2_: 1–4 m^3^/h (O_2_ < 10 ppm)	10–20	67	[47]
Fe with Fe_3_O_4_ shell	Glove box with N_2_ flow, supported inverse H_2_/air diffusion flame	Ferrocene	Xylene, tetrahydrofuran	0.2–0.8		4	2.5	N_2_: 1.5 m^3^/h (H_2_: 0.76 m^3^/h, air: 0.8 m^3^/h)	30–80		[156]
Co	Glove box with radial N_2_ or CO_2_ flow, two adsorption columns	Co 2-ethylhexanoate	Tetrahydrofuran		2.2/1.2	4.5–6	5	N_2_ or CO_2_: 25 (O_2_ < 100 ppm)	30		[57]

**Figure 20:**
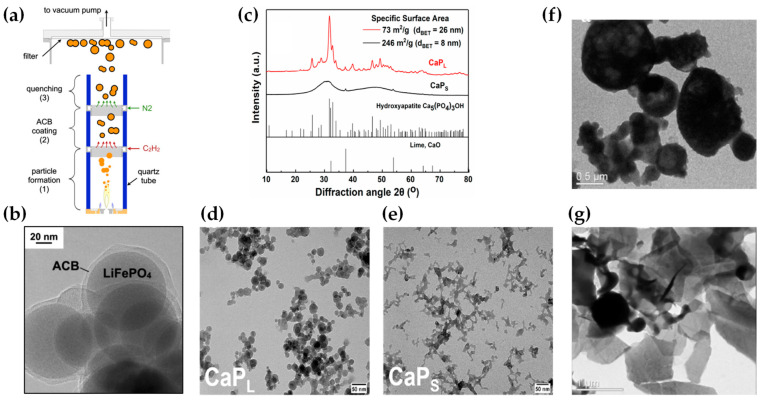
(**a**) Illustration of an enclosed FSP configuration, comprising a particle formation zone (1), an acetylene carbon black (ACB)-coating region (2), and a quenching zone (3). The precise regulation of O_2_ stoichiometry within the ACB-coating area is achieved by enclosing the unit with quartz tubes. Subsequently, the coated particles are subjected to cooling using nitrogen (N) at the conclusion of the coating zone to prevent carbon black combustion upon exposure to ambient air during the filtration process. (**b**) TEM image of as-prepared segregated ACB and LiFePO_4_ nanostructures. Reprinted from [149], with permission from Elsevier. (**c**) The XRD pattern of the calcium phosphate (CaP) nanoparticles is presented. Depending on the FSP synthesis conditions employed, the resultant nanoparticles manifest either crystalline or amorphous characteristics. Predominant diffraction peaks are attributed to hydroxyapatite; Ca_5_(PO_4_)_3_OH, though the presence of CaO, is also detected. TEM micrographs of the freshly synthesized (**d**) CaP_L_ and (**e**) CaP_S_ materials are provided. The CaP_L_ nanoparticles display a spherical morphology characterized by a loosely agglomerated structure. In contrast, the CaP_S_ particles are evidently fused, with discernible sintered necks. Reprinted from [153]. (**f**,**g**) TEM images for as-synthesized vanadium phosphate (VOPO_4_) particles from (**f**) sucrose-based solutions and (**g**) DMF-based solutions. Reprinted from [146].

Regarding FSP-synthesized phosphates, several investigations have been conducted from the onset of the 21st century to the present day. These studies encompass a broad spectrum of research, examining the properties, applications, and advancements in the realm of phosphates produced via FSP methodology. Kang et al. [157] explored the synthesis of Sr_5_(PO_4_)_3_Cl:Eu^2+^ phosphor particles through a dual-method approach, employing both conventional spray pyrolysis and flame spray pyrolysis techniques. Subsequently, these synthesized particles underwent post-treatment at a high temperature of 1000 °C over a duration of 3 h within a controlled reducing atmosphere consisting of a gas mixture containing 5% H_2_/N_2_. The primary objective of this post-treatment was to activate and optimize the luminescent properties associated with the europium (Eu) component integrated into the phosphor matrix. This process served to enhance the photoluminescence (PL) intensity for light-emitting diode applications [157]. Another work [146] focused on the polymorphic compound VOPO_4_, known for its exceptional catalytic and electronic properties. For the first time, the utilization of FSP, as an alternative to the conventional hydrothermal synthesis method, is explored to produce various VOPO_4_ polymorphs [146]. The precursor materials employed in this process consisted of ammonium-based salts, i.e., vanadium and phosphorus, dissolved in an aqueous solution. The resulting FSP products were characterized as hollow, amorphous particles [137] with dimensions 2 to 10 μm and shell thicknesses between 200 and 300 nm (see Figure 20f,g). Last, the study highlighted the size-dependent stability of different VOPO_4_ polymorphs and examined the influence of distinct precursors in stabilizing the alpha-II and Beta VOPO_4_ polymorphic forms [146].

Nanostructured iron phosphate particles hold appeal for the fortification of staple food products, such as rice and bread, on a large scale [158,159], as well as for their utility in lithium-ion battery materials [160]. Rohner et al. [147] aimed to synthesize FePO_4_ nanoparticles for bioavailability and assess their toxicity in rats. Amorphous, spherical FePO_4_ nanopowders were produced via FSP and compared with commercial FePO_4_ and FeSO_4_. The FePO_4_ nanoparticles, both commercial and FSP-produced (mean particle sizes: 30.5 and 10.7 nm), exhibited promising SSA and in vitro solubility, with RBV values close to FeSO_4_. Importantly, no toxicity indications were observed in histological examinations and thiobarbituric acid reactive substances (TBARS) analysis, suggesting that reducing poorly soluble Fe compounds to the nanoscale could enhance their suitability for human nutrition [147]. Likewise, Rudin and Pratsinis [148] produced FePO_4_ nanostructured particles FSP, utilizing cost-effective precursor materials. The resulting powders consisted of a fraction of large (mostly > 50 nm) particles made by droplet-to-particle conversion that consisted of maghemite iron oxide (Fe_2_O_3_) and a small fraction of the desired amorphous FePO_4_. The FePO_4_ powders exhibited excellent solubility in dilute acid, an indicator of relative iron bioavailability [148]. Waser and colleagues [149] employed FSP to synthesize nano-sized spherical LiFePO_4_ (58 nm)–carbon core-shell (≈5 nm) particles (see Figure 20b) in a single-step process, achieving a production rate of 7 g per hour, with the intended application for use in Li-ion batteries. A customized FSP apparatus, enclosed within three quartz glass tubes, was employed for this study (as depicted in Figure 20a) [149]. At specific heights, precisely 40 cm and 55 cm above the burner, gaseous C_2_H_2_ and N_2_, respectively, were introduced into the gas stream. This configuration partitioned the reactor into three clearly defined zones: [i] an oxygen-rich core particle formation zone, [ii] an oxygen-deficient region dedicated to C_2_H_2_ pyrolysis and carbon coating, and [iii] a designated area for quenching and cooling processes [149]. Li-acetylacetonate, iron(III)-acetylacetonate, and tributylphosphate were combined in a stoichiometric ratio of 1:1:1 (mole ratio Li:Fe:P) and subsequently dissolved within an equimolar mixture of 2-ethylhexanoic acid, toluene, diethylene glycol monobutyl ether, and ethanol. This process yielded a precursor solution with a concentration of 0.24 mol L^−1^. Additionally as evidenced, the work of Badding et al. [150] pertains to an FSP technique for the production of nanoscale lithium metal phosphate ceramic powders. The precursor solution may incorporate an excess of lithium ranging from 1% to 20% concerning the stoichiometric composition of the ceramic powder. The resulting nanoscale ceramic powders are characterized by nominal compositions denoted as Li_x_Al_2−y_M_y_(PO_4_)_3_ (<50 nm diameter), with M representing either titanium (Ti) or germanium (Ge), 1 ≤ x ≤ 2 and 1.3 ≤ y ≤ 1.9. Various precursors, such as Li t-butoxide, Al tri-sec-butoxide, Ti isopropoxide, trimethyl phosphate, Li chloride, or Ge ethoxide, were dissolved in 2-methoxyethanol or ethanol to produce the aforementioned lithium metal phosphate nanopowders [150].

Last but not least, it is worth noting that in the past decade and up to the present, various other studies have employed FSP to fabricate nanoscale calcium phosphates (CaP) [161], representing a significant area of scientific investigation and technological development in the field of biomaterials and biomedicine [151,152,153]. Nanometer-sized biphasic calcium phosphate (BCP) powders, featuring various Ca/P molar ratios to achieve specific phase ratios of hydroxyapatite (HA) and beta-tricalcium phosphate (b-TCP), were systematically synthesized using a high-temperature FSP process [151]. These BCP powders displayed spherical morphologies and exhibited narrow size distributions (mean particle size = 38 nm), irrespective of the Ca/P ratios employed. The composition ratio of Ca/P was precisely controlled within the range of 1.500 to 1.723 in the spray solution, allowing for systematic adjustment of the required HA/TCP phase ratios. The FSP configuration comprised an ultrasonic droplet generator and quartz reactor. The precursors employed in this process consisted of calcium nitrate and tetrahydrate diammonium phosphate dissolved in a mixture of ethyl alcohol and distilled water. The degradability of the synthesized BCP powders was studied by the dissolution of calcium ions in a buffer solution under conditions mimicking a human physiological environment [151]. Ataol and colleagues [152] conducted an evaluation of the synthesis of biocompatible nano CaP particles with osteoconductive and osteoinductive properties. These particles were synthesized using an industrially applied aerosol-derived FSP method, with a focus on their potential applications in the biomedical field. The characterization results confirmed that nanometer-sized, amorphous, spherical CaP particles were produced with an average primary particle size of 23 nm. Urine-derived stem cells, resembling mesenchymal stem cells, were exposed to the synthesized amorphous nanoparticles (5–50 µg/mL) with no cytotoxicity. Nanoparticle-treated cells exhibited increased alkaline phosphatase (ALP) activity, indicating osteogenic differentiation, although a slight decline in ALP activity occurred at the highest Ca:P ratios (50 µg/mL) on day 7. These findings suggest that the CaP nanoparticles generated in this study hold promise as potential biomaterials for biomedical applications [152].

Nanoparticles present considerable potential as drug delivery vehicles in the realm of biomedicine, characterized by their substantial surface-to-volume ratio that enables efficient drug loading [162]. In [153], the authors utilized FSP to produce spherical CaP nanoparticles (primary particle diameter of 8 and 26 nm for two samples) with customizable properties (see Figure 20c–e). CaP nanoparticles doped with 5 at% europium (vs. Ca) enabling their detection through luminescence monitoring. These CaP nanoparticles were loaded with model proteins and peptides, and the factors affecting their loading capacity were explored. They also successfully load LL-37, an antimicrobial peptide currently in clinical trials, onto CaP nanoparticles, achieving high loading efficiency and protection against proteolysis in vitro. Importantly, LL-37 maintains its antimicrobial activity against specific pathogens when loaded onto nanoparticles, emphasizing the potential of nanocarriers for optimizing the therapeutic performance of biological drugs [153].

**Figure 21:**
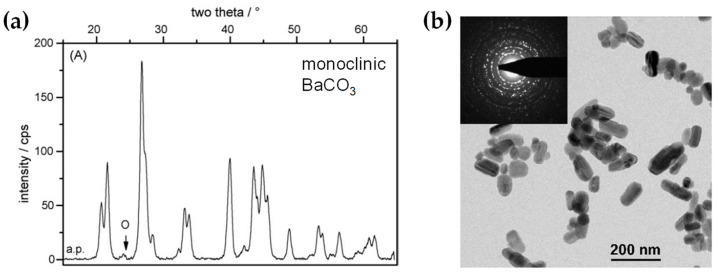
(**a**) XRD pattern of the as-synthesized barium carbonate (BaCO_3_) is presented. Reflections not labeled are attributable to the monoclinic phase of BaCO_3_. Only minute traces of the orthorhombic phase (O) have been detected. (**b**) TEM showcasing nanoparticles of BaCO_3_ synthesized via flame-based methods. The inset provides a representation of the corresponding electron diffraction pattern. Reprinted from [155], with permission from Elsevier.

It is noteworthy that a limited body of research, as documented in studies [154,155], has pursued the synthesis of metal carbonates by FSP. Huber and colleagues demonstrated the production of calcium carbonate (CaCO_3_) nanoparticles of 20–50 nm by FSP [144]. This method involved the combustion of designated calcium-containing precursors, leading to the formation of either amorphous or crystalline calcium carbonate particles, contingent upon the specific spray flow conditions employed. Moreover, Strobel et al. fabricated nanoparticles of bean-like-shaped barium carbonate (BaCO_3_) with sizes ranging from 50 to 100 nm using FSP (refer to Figure 21). They used barium(II) 2-ethylhexanoate precursor dissolved in ethanol. The key aspect of this preparation process was the rapid quenching, which led to the unique occurrence of pure monoclinic BaCO_3_ formation.

### 3.3. Engineering of Metallic Nanoparticles by FSP

**Figure 22:**
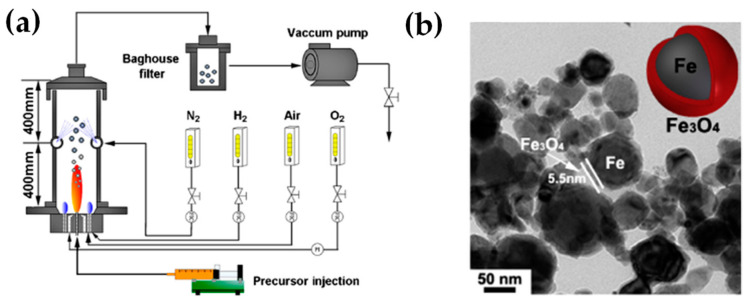
(**a**) Illustration depicting the experimental arrangement employed for FSP synthesis of magnetic nanoparticles. Specifically, the reducing flame spray synthesis (RFSP) was executed under a controlled nitrogen atmosphere, with the pilot flame constituted of an O_2_/H_2_/air mixture. (**b**) TEM image of flame-made Fe/Fe_3_O_4_ nanostructures. Reprinted from [156], with permission from Elsevier.

Reducing FSP has been shown to allow for the production of metallic nanoparticles (Bi, Cu, Fe, Co). Highly pure metallic bismuth (Bi) nanoparticles [46], with a purity exceeding 98%, were synthesized using a modified flame spray synthesis method conducted in an inert N_2_ atmosphere, with a radial flow rate of 25 L min^−1^. This synthesis process involved the oxygen-deficient combustion (O_2_ < 100 ppm) of a precursor derived from bismuth carboxylates. Scanning electron micrographs revealed the presence of nearly spherical nanoparticles with diameters of 20–80 nm. Copper nanoparticles (C/Cu) featuring an average carbon coating thickness of approximately 1 nm were synthesized by Athanassiou et al. using a reducing FSP method with a N_2_ atmosphere. TEM images of the C/Cu powder showed that the product consisted of spherical nanoparticles with a diameter of 10–20 nm [47]. Using R-FSP, Li et al. [156] synthesized metallic spherical Fe nanoparticles (size: 30–80 nm) encapsulated by a magnetite Fe_3_O_4_ core-shell structure, exhibiting a uniform thickness of 4–6 nm (see Figure 22b). Precursors were based on ferrocene in a solvent mixture xylene and tetrahydrofuran. The ignition of the spray occurred through the utilization of a supported inverse H_2_/air diffusion flame with specified flow rates (H_2_: 0.76 m^3^/h, air: 0.8 m^3^/h) (see Figure 22a). Additionally, cooling of the spray flame was achieved by introducing 1.5 m^3^/h of N_2_. The systematic investigation revealed that controlled nonstoichiometric combustion of the precursor solution leads to morphological and compositional variations in flame-synthesized metallic Fe nanostructures. They proposed a competitive mechanism between in situ flame combustion reduction and oxidation reactions to elucidate the metallic Fe formation [156]. Cobalt nanoparticles [57] were systematically produced through R-FSP conducted under strongly reducing conditions, akin to [46]. A sintered metal tube encompassing the flame facilitated radial inflow of an inert mixing gas (N_2_ or CO_2_) at a flow rate of 25 L min^−1^, ensuring stable combustion. These spherical nanoparticles, with diameters of 20–60 nm, exhibited a metallic face-centered cubic cobalt structure. For further details regarding the chemical synthesis process, please refer to Table 7. Notably, the metal particles were shielded from oxidation by a surface layer measuring less than 1 nm in thickness, primarily composed of cobalt oxide.

### 3.4. Engineering of Quantum Dots by FSP

Quantum dots (QDs)—with early contributions from pioneering Nobel laureates Ekimov, Brus, and Bawendi [163,164,165]—represent quasi-zero-dimensional (0D) nanomaterials that have garnered considerable interest among researchers [166,167,168,169,170,171]. QDs are semiconductor nanoparticles characterized with dimensions ≪ 10 nanometers. When their dimensions become commensurate with or smaller than the Bohr radius of the respective material, they manifest distinctive characteristics positioned between those of bulk semiconductors and discrete molecules [168,172]. This phenomenon is distinguished by the confinement of excitons within all three spatial dimensions, resulting in the quantization of their energy levels into discrete states [172,173]. A complex challenge concerning the photocatalytic properties of QDs pertains to the differentiation between the advantages arising from increased specific surface areas, stemming directly from reduced particle size, and the emergent properties of the nanolattice, which underpins fundamental solid-state physics [174].

So far, QDs are typically synthesized through [i] top-down syntheses or [ii] bottom-up approaches [170]. For [i], the most commonly used to achieve QDs are electron beam lithography [175,176] and etching processes [177,178] while the wet-chemical (sol-gel [179,180,181], microemulsion [182,183], hot-solution decomposition [165,184]), and vapor-phase methods (molecular beam epitaxy [185], sputtering [186], chemical vapor deposition [187]) are included in [ii]. The aforementioned methods are characterized by extended processing times, multiple steps, and yielding production rates on the order of milligrams per day.

**Figure 23:**
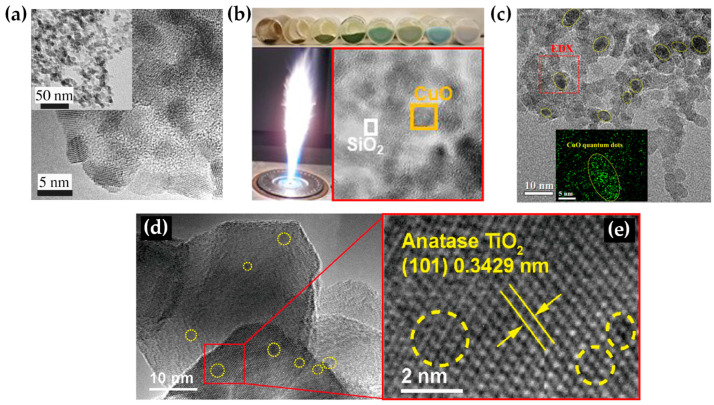
(**a**) A HR-TEM image of as-prepared mixed silica-ZnO crystallites. This image prominently displays the crystalline lattice structure of the ZnO component. Additionally, an inset is provided, offering a lower magnification view that elucidates the overall morphology of the powder under investigation. Reprinted from [188], with the permission of AIP Publishing. (**b**) Combustion flame of the SiO_2_-metal oxide solution with all the produced colored metal oxide QDs and the TEM image of CuO QDs. Reprinted from [189]. (**c**) TEM analysis of CuO-SrTiO_3_ nanostructures, where the CuO QDs are distinctly delineated by dashed yellow circles. *Inset:* elemental mapping of CuO QDs within a specific region of interest. Reprinted (adapted) with permission from [130]. Copyright 2021 American Chemical Society. (**d**,**e**) HR-TEM images and the locally enlarged HR-TEM images of CQDs/TiO_2_-C. Reprinted from [190], with permission from Elsevier.

Regarding the FSP production of metal-oxide QDs, there are only a few studies, see Table 8. Mädler et al. [188] reported FSP synthesis of stable zinc oxide quantum dots (ZnO QDs) down to 1.5 nm in diameter (see Figure 23a) stabilized and prevented from growth by adding controlled amounts of silica during spray combustion of Zn/Si precursors [167]. These ZnO QDs exhibited a quantum size effect with a blue shift of light absorption, and their band-gap energy of the ZnO QDs was shown to increase with silica content in the spray and particles consistently [167]. Correspondingly, Riad and colleagues [189] had proposed embedding quantum dots in amorphous matrices such as silica (see Figure 23b). The study demonstrated that FSP can be used to synthesize QDs of many metal oxide semiconducting materials, including TiO_2_, ZnO, SnO_2_, and CuO, embedded in a SiO_2_ matrix [168]. The resulting spherical QDs (mean sizes: 2–8 nm) were found to have a wide range of band gap energies, which could be controlled by adjusting the metal oxide material and the silica content. The SiO_2_ matrix provided higher mechanical, thermal, and/or chemical stability to the QDs, making them suitable for various applications. Yuan et al. [130] explored the utilization of FSP-made CuO(QD)-SrTiO_3_ nanocatalysts for the full oxidation of lean CO and CH_4_. Good performance was achieved due to CuO QDs and metal–support interaction (see Figure 23c). The SrTiO_3_ perovskite support was discovered to efficiently block CuO quantum dot sintering at high temperatures, resulting in good sintering and water deactivation resistance.

Bi et al. [190] fabricated in situ a nano–TiO_2_ composite material modified by incorporation of internal and external C-species. Specifically, the authors developed a heterostructure composed of carbon quantum dots (CQDs) and TiO_2_-C through FSP and post-FSP thermal treatment (under Ar/O_2_ atmosphere) (see Figure 23d,e). During the millisecond-scale reaction process, a portion of the residual carbon resulting from the incomplete combustion of ethanol infiltrates the TiO_2_ lattice, giving rise to interstitial carbon (C_i_) and substituent carbon (C_s_). Concurrently, the remaining amorphous CQDs adsorb onto the TiO_2_ surface. Their investigations, employing in situ temperature-programmed X-ray photoelectron spectroscopy (in situ TPXPS) in combination with various analytical techniques, confirmed the successful fabrication of the CQDs/TiO_2_-C heterostructure through the flame-based approach, with the atomic ratio of the two carbon species approximating 1:1. This investigation sheds light on the impact of a novel electron transfer pathway established between lattice-bound carbon (C) and CQDs within the CQDs/TiO_2_–C system during the process of CO_2_ photoreduction, resulting in a conversion efficiency of 46 μmol g^−1^ h^−1^ and nearly 100% selectivity for CO_2_-to-CO conversion.

### 3.5. Engineering of Nanoplasmonics by FSP

Nanoplasmonics is a multidisciplinary field of study within the realm of nanophotonics that investigates the interaction between electromagnetic waves and collective oscillations of free electrons, known as surface plasmons, at the interface of metals and dielectric materials [192,193]. In the context of nanoscale dimensions, the phenomenon of localized surface plasmon resonance (LSPR) becomes prominently evident [194,195]. LSPR can be analyzed as comprising both radiative and nonradiative processes: [i] The radiative plasmon decay encompasses the phenomenon of light scattering by the surface of plasmonic particles, thereby giving rise to the distinctive and vibrant colors associated with noble metals. It involves the transfer of energetically “hot” electrons upon irradiation, characterized by substantial kinetic energy, to a compatible acceptor species [196]. Additionally, it engenders the creation of localized amplified electric near-fields, the so-called hot spots owing to the confinement of surface plasmons and incoming photons in the immediate vicinity of the particle [197]. [ii] The nonradiative plasmon decay and the ensuing thermal energy generation may be comprehended as the dissipation of electromagnetic energy into thermal energy. This phenomenon has led to the emergence of a distinct subfield known as thermoplasmonics [198], which harnesses the photothermal characteristics of metallic nanoparticles for various applications. Overall, plasmonics finds diverse applications in various scientific and technological domains, including nanoscale optical waveguiding, sensing, imaging, and enhancing the efficiency of photonic devices, making it a promising area for advancing our understanding of light–matter interactions and facilitating the development of innovative technologies across multiple disciplines. Hereby in this subsection, we shall delineate the research endeavors in which the FSP technique has been successfully employed to engineer plasmonic nanoensembles, with the corresponding characteristics listed in Table 9.

The concept of plasmons was originally postulated in the year 1952 by David Pines and David Bohm [199]. These plasmons were subsequently demonstrated to originate from a Hamiltonian governing the long-range electron–electron correlations [200]. Given that plasmons represent the quantized manifestations of classical plasma oscillations, a substantial portion of their characteristics can be directly deduced through the framework of Maxwell’s equations [201]. Nevertheless, it took approximately half a century for the synthesis of plasmonic nanostructures utilizing the FSP methodology to be achieved. Plasmonic phenomena are principally ascribed to noble metallic elements, notably silver (Ag), gold (Au), and copper (Cu), notwithstanding the potential for plasmonic attributes to manifest in other metallic and material compositions, contingent upon specific environmental conditions. Ag and Au, in particular, display strong plasmonic properties primarily in the visible and near-infrared regions, while copper also exhibits plasmonic behavior, albeit less pronounced, especially in the visible and ultraviolet spectrum.

**Figure 24:**
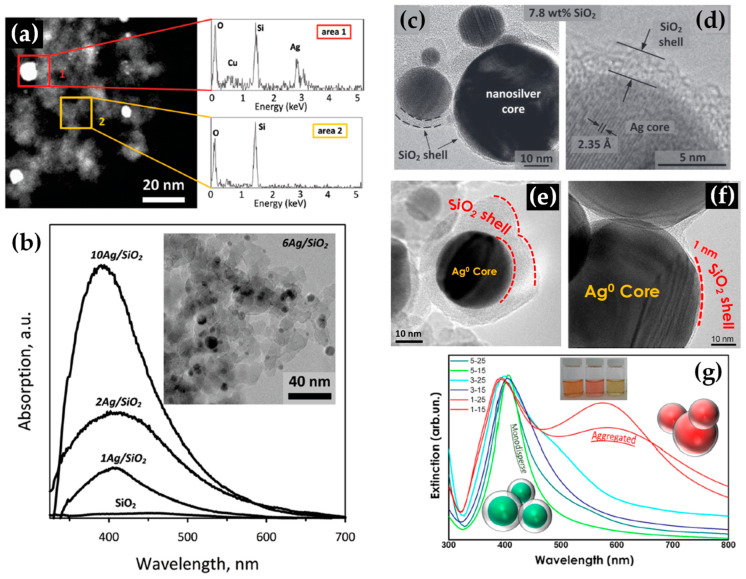
(**a**) STEM image of the 2Ag/SiO_2_ composite material EDX analysis: area 1, containing silver, and area 2, representing pure SiO_2_. (**b**) Diffuse reflectance ultraviolet/visible (UV/vis) spectra for various xAg/SiO_2_ compositions, where x denotes the silver concentration. A consistent plasmon absorption band of Ag metal at 410 nm was observed in all Ag-containing samples. TEM image, shown as an inset, depicted Ag nanoparticles (dark dots) dispersed on nanostructured silica support (gray) in the 6Ag/SiO_2_ nanostructure. Reprinted (adapted) with permission from [202]. Copyright 2010, American Chemical Society. (**c**,**d**) TEM images of the nanosilver coated with 7.8 wt% SiO_2_ are presented. Reproduced with permission from ref. [78]. Copyright 2010 Wiley-VCH. (**e**,**f**) TEM images of (**e**) 5–25 SiO_2_@Ag^0^ NPs (SiO_2_ thickness = 5 nm), and (**f**) 1–25 SiO_2_@Ag^0^ NPs (SiO_2_ thickness = 1 nm). (**g**) UV/vis spectra were recorded for suspensions of SiO_2_@Ag^0^ NPs with uniform particle size across three distinct variants: 1–15, 25 (SiO_2_: 1 nm), 3–15, 25 (SiO_2_: 3 nm), and 5–15, 25 (SiO_2_: 5 nm). The inset of the figure presents photos of these particle suspensions. Additionally, schematic representations of the particles are provided to visually convey the influence of shell thickness. Reprinted (adapted) with permission from [80]. Copyright 2019 American Chemical Society.

**Figure 25:**
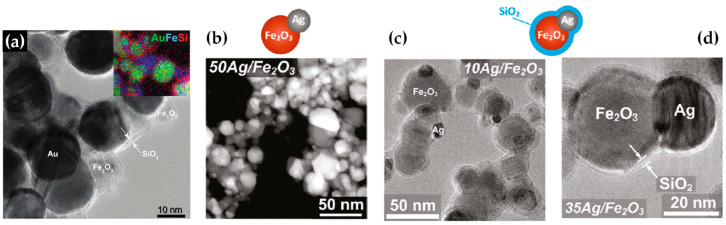
(**a**) HR-TEM image depicting nanoparticles consisting of Au/Fe_2_O_3_ cores enveloped by amorphous SiO_2_ shells, measuring 2.6 nm in thickness with the SiO_2_ content in these shells quantified at 5.7 wt%. *Inset:* Elemental EDXS mapping for all three elements (Au, Fe, Si) together in a merged image. Reproduced with permission from ref. [203]. Copyright 2014 Wiley-VCH. (**b**) A HAADF-STEM image, characterized by Z-contrast, displaying the uncoated 50Ag/Fe_2_O_3_ sample. In addition, TEM images are presented for (**c**) the SiO_2_-coated 10Ag/Fe_2_O_3_ sample and (**d**) the SiO_2_-coated 35Ag/Fe_2_O_3_ sample. Above these visual representations, schematic diagrams are included, illustrating the structural characteristics of both uncoated and SiO_2_-coated particles. Reprinted (adapted) with permission from [204]. Copyright 2011 American Chemical Society.

Several scholarly studies have documented the production of plasmonic materials integrated with support matrices (metal oxides) through the FSP method. The predominant synthesis technique for plasmonic materials involves the use of SiO_2_ as a core-shell structure (see Figure 24). Silica (SiO_2_) encapsulation of plasmonic Ag nanoparticles through FSP offers a host of valuable advantages. This method ensures stability by protecting silver nanoparticles from oxidation, while also enhancing their plasmonic properties. Researchers can precisely tailor the nanoparticles’ properties by controlling the thickness of the silica shell. Moreover, the biocompatibility of silica makes these nanoparticles suitable for diverse medical applications. SiO_2_ shell effectively prevents particle aggregation, ensuring stability in various solvents. Additionally, the silica surface provides a platform for easy functionalization, allowing for targeted interactions. These attributes collectively position them as valuable tools in nanotechnology, materials science, and biotechnology applications (see Figure 25).

In 2004, Johannessen and colleagues [205] synthesized supported noble metals like Au/TiO_2_ using a quench-cooling device for controlling the residence time at high temperatures in the FSP technique. Hannemann et al. [206] fabricated monometallic Au and bimetallic Au–Ag nanostructures by FSP and characterized their catalytic activity in CO oxidation. The particle size of monometallic Au nanoparticles ranged from 1 to 6 nm, while in the case of bimetallic nanoparticles, the size exhibited a range of 1–10 nm and 25–40 nm [206]. Over the years, a considerable body of research has been dedicated to the synthesis of plasmonic nanoparticles encapsulated within SiO_2_ and various other matrixes for diverse applications, a trend that continues to be relevant in contemporary studies. Sotiriou and Pratsinis [79] synthesized Ag/SiO_2_ nanostructures and conducted investigations on the mitigation of nanosilver toxicity, elucidating two mechanisms: (i) the prevention of direct cellular contact with silver and (ii) the inhibition of the release of toxic Ag^+^ ions. In separate studies [78,202], the same researchers, in collaboration with other colleagues, discriminated between the antibacterial impacts of Ag^+^ ions and nanosilver particles. They found that smaller Ag nanoparticles (< 10 nm) exhibited a predominant antibacterial effect due to the release of high concentrations of Ag^+^ ions, whereas larger Ag nanoparticles resulted in antibacterial effects comparable to those of released Ag^+^ ions and nanosilver particles. All the produced Ag nanoparticles displayed a distinct peak at approximately 400 nm in their UV/visible spectra, which corresponds to the plasmon resonance frequency characteristic of Ag nanoparticles. The precise control over the size of the Ag core and the presence of a SiO_2_ coating layer are particularly vital properties in numerous applications, notably in the context of bio-labeling [207]. In a related investigation [208], the impact of precursor composition on the characteristics of Ag/SiO_2_ nanoparticles was examined. Nanosilver synthesized from Ag-acetate and hexamethyldisiloxane (HMDSO) exhibited a unimodal size distribution, while that from Ag-nitrate and HMDSO or tetraethyl orthosilicate precursor solutions displayed a bimodal size distribution. The antibacterial activity against *E. coli* was evaluated, revealing that Ag-nitrate-derived nanosilver exhibited similar antibacterial effects as Ag-acetate-derived nanosilver, with the fine mode of distribution primarily responsible for bactericidal properties due to the release of silver ions. Furthermore, antibacterial effects correlated best with nanosilver surface area concentration in suspension rather than other magnitudes, suggesting surface area concentrations should be considered in toxicological studies [208].

**Table 9:** The literature summary of FSP characteristics/conditions for plasmonic hybrid nanostructures.

Nano- Structure	FSP Configuration	Precursor(s)	Solvent(s)	Molarity (mol L^−1^)	Pilot Flame O_2_/CH_4_ (L min^−1^)	Precursor Flow (mL min^−1^)	Oxygen Flow (L min^−1^)	Sheath Oxygen (L min^−1^)	Size (nm)	Ref.
Ag/SiO_2_	Open-flame FSP reactor, mixed precursors	(a): [Ag nitrate, HMDSO], (b): [Ag acetate, HMDSO], (c): [Ag nitrate, HMDSO]	(a): [Ethanol, diethylene glycolmonobutyl ether], (b): [2-ethylhexanoic acid, toluene], (c): [2-propanol, tetraethyl orthosilicate]	0.5					~150	[202]
Core-shell Ag/SiO_2_	Enclosed by 2 quartzes tubes of 40 cm + ring deposition with N_2_ flow (0.8 and 10–30 L min^−1^)	Ag-acetate, HMDSO	Acetonitrile, 2-ethylhexanoic acid		3.2/1.5	5	5	40	Ag: 10–30, SiO_2_: 3–6	[79]
Core-shell Ag/SiO_2_	Enclosed by quartz tube of 22 cm + ring deposition with N_2_ flow (0.3–3 and 10–15 L min^−1^)	Ag-acetate, HMDSO	Acetonitrile, 2-ethylhexanoic acid	0.3–0.5 SiO_2_: 8–27 wt%	5/2.5	5–7	5	5–20	Ag: 15–25, SiO_2_: 1–5	[80]
Au/TiO_2_	Open-flame FSP reactor, mixed precursors	Ti(IV)-isopropoxide, dimethyl-Au(III) acetylacetonate	Xylene, pyridine (8/2)			3.1	6		Au: 1–2, TiO_2_: 9–10	[209]
[monometallic Au]–TiO_2_, Fe_2_O_3_/Fe_3_O_4_, SiO_2_ [bimetallic Au/Ag]–TiO_2_, Fe_2_O_3_/Fe_3_O_4_, SiO_2_, Al_2_O_3_	Open-flame FSP reactor, mixed precursors	Dimethyl-Au-(III)-acetylacetonate, silver- (I)-benzoate, Ti(IV)-isopropoxide, Fe(II) naphthenate, tetraethyl ortho-silicate	Xylene, pyridine		3.5	3	3		Au: 1–6, Au/Ag: 1–10, 25–40	[206]
Ag/Au–SiO_2_	v FSP reactor, mixed precursors	Ag acetate, Au acetate, HMDSO	Acetonitrile, 2-ethylhexanoic acid	0.15		5	5		4–14	[210]
Ag/SiO_2_	Closed FSP reaction zone with a H_2_/air diffusion flame	Ag nitrate, tetraethyl orthosilicate	Ethanol			3	5	H_2_/air: 2/25 L min^−1^	Ag: ~30, SiO_2_: 1–4	[211]
TiO_2_– Ag/TiO_x_	Open-flame FSP reactor, mixed precursors	Ag acetate, Ti(IV)-isopropoxide	Acetonitrile, 2-ethylhexanoic acid	0.16	3.2/1.5	3, 8	5	20	${\mathrm{TiO}}_{2}:\;\sim$20, Ag: 3–5	[212]
SiO_2_-coated Ag/Fe_2_O_3_	Enclosed by 30 cm quartz tube + ring deposition with N_2_ flow (15 L min^−1^) + 25 cm quartz tube	Ag acetate, Fe(III) acetylace- tonate, HMDSO	Acetonitrile, 2-ethylhexanoic acid	Fe_2_O_3_: 0.5, Ag: 0–50 wt% SiO_2_: 23 wt%	3.2/1.5	5	5	40	Fe_2_O_3_: 15, Ag: 10–20, SiO_2_: 1–2	[204]
SiO_2_-coated Au/Fe_2_O_3_	Enclosed by 30 cm quartz tube + ring deposition with N_2_ flow (16 L min^−1^) + 25 cm quartz tube	Au(III) acetate, Fe(III) acetylace- tonate, HMDSO	Acetonitrile, 2-ethylhexanoic acid	Au: 0.25, Fe_2_O_3_: 30 at%, SiO_2_: 0–13 wt%	3.2/1.5	5	5	40	Fe_2_O_3_: 50–100, Au: 30, SiO_2_: 2.6	[203]
Ag/Ca_3_(PO_4_)_2_	Open-flame FSP reactor, mixed precursors	Ag acetate, Ca hydroxide, tributyl phosphate	2-ethylhexanoic acid, toluene	0.75 Ca/P: 1.5 Ag: 0–10 wt%	5	5	5	4	Ca_3_(PO_4_)_2_: 20–50, Ag: 1–2	[213]

**Figure 26:**
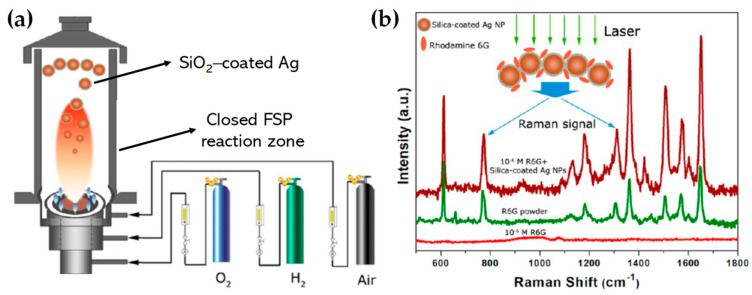
(**a**) A schematic figure illustrating the FSP setup employed in the synthesis of silica-coated silver nanoparticles. (**b**) The Raman spectra analysis encompassed two distinct samples: pure rhodamine R6G powder and R6G molecules at a concentration of 10^−6^ mol L^−1^, both with and without the presence of 5 wt% SiO_2_-coated Ag nanoparticles. The presence of plasmonic particles induces the surface-enhanced Raman scattering (SERS) effect. Reprinted (adapted) with permission from [211]. Copyright 2013, American Chemical Society.

Plasmonics give rise to surface-enhanced Raman spectroscopy (SERS) mechanism by facilitating the enhancement of Raman signals. This stems from their ability to create localized electromagnetic field enhancements at the nanoscale, leading to increased scattering cross sections and, thus, contributing significantly to the amplification of the Raman signal of analyte molecules in close proximity [214,215]. In the study of Hu et al. [211], the ultrathin SiO_2_ shell (1 nm), served a dual purpose by effectively preventing the coalescence of Ag nanoparticle cores at elevated temperatures and functioning as a protective layer for the SERS-active nanostructure. Silica-coated Ag nanoparticles form agglomerates within a large temperature gradient zone, maintaining nanometer-scale gaps between them without direct contact (see Figure 26a). This distinctive feature leads to the creation of numerous Raman hot spots at the interstitial regions among the active Ag core sites, enhancing the overall performance of the SERS substrate. The results [211] demonstrated that the maximum enhancement factor can reach approximately 10^5^, with a detectable concentration as low as 10^−10^ mol L^−1^ for rhodamine 6G (R6G) molecules, highlighting the potential of this unique nanostructure for SERS applications (as shown in Figure 26b).

Recently, Moularas et al. [80] engineered core-shell nanoassemblies composed of Ag/SiO_2_ with diverse SiO_2_ layer thicknesses (1, 3, and 5 nm (see Figure 24e–g)). These nanostructures were synthesized employing the ring deposition FSP methodology. They investigated the thermoplasmonic heat generation efficiency of these nanostructures [80] and examined the mechanisms underlying plasmon-mediated hot-electron transfer from these plasmonic structures to redox-active metals via hexavalent chromium reduction [81]. In this study, they presented a novel experimental concept and methodology for capturing and quantitatively evaluating plasmon-induced hot electrons produced by core-shell plasmonic nanoaggregates, utilizing EPR spectroscopy [81]. Proton-coupled electron transfer (PCET) reactions, which involve the simultaneous transfer of a proton and an electron, are pivotal in numerous chemical and biological processes. We have [88] introduced a novel phenomenon, plasmon-enhanced PCET, which was achieved using flame-made SiO_2_-coated Ag nanoparticles functionalized with gallic acid (GA), a natural antioxidant capable of PCET. These GA-functionalized nanoparticles exhibit enhanced plasmonic response in the near-IR range due to GA-induced particle agglomeration. Near-IR laser irradiation induces localized hot spots on the nanoparticles, facilitating PCET by lowering the GA-OH bond dissociation energy through plasmon energy transfer [88].

Nanosilver particles (7–30 nm) immobilized on nanosilica were fabricated by Fujiwara et al. utilizing an open FSP reactor where the two (Ag, SiO_2_) precursors were mixed together at various precursor feed/dispersion O_2_ ratios [88]. The presence of Ag_2_O on the nanosilver surface was confirmed by UV-vis spectroscopy and quantified using thermogravimetric analysis and mass spectrometry. The release of Ag^+^ ions in deionized water was linked to the dissolution of Ag_2_O on the nanosilver surface, with rapid pH increase inhibiting further dissolution. However, exposure to CO_2_ in ambient air led to pH reduction, promoting metallic Ag dissolution and Ag^+^ ion release. This study investigated Ag^+^ ion release under different conditions, including CO_2_ presence, and its relevance to nanosilver’s antibacterial activity [88].

**Figure 27:**
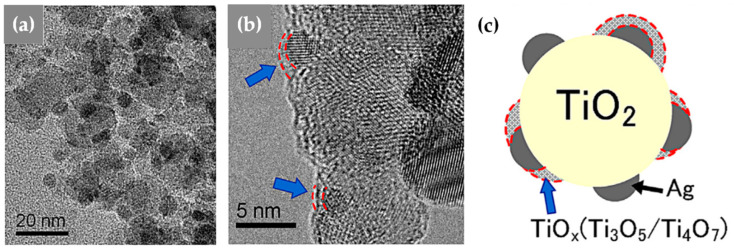
(**a**) TEM image of 20Ag/TiO_2_, synthesized under the condition X/Y = 8/5 (precursor feed rate/dispersion gas) accompanied by selected (**b**) high-resolution images for detailed examination. Notably, disordered titanium oxide, observable on both the nanosilver and TiO_2_, is highlighted using blue arrows and red dashed lines. (**c**) An illustrative diagram is included, depicting the formation of titanium suboxide (Magnéli phases) on nanosilver and TiO_2_, resulting from robust metal–support interactions (SMSI). Reprinted from [212], with permission from Elsevier.

Visible-light active, black TiO_2_-Ag nanoparticles were synthesized by an FSP setup [205] that promotes the formation of Magnéli Ti_4_O_7_ and Ti_3_O_5_ nano-spots [205] on their surfaces through the mediation of strong metal–support interactions (SMSI) (see Figure 27) [212]. The TiO_2_ precursor (Ti-isopropoxide) was combined with various Ag precursor (silver acetate) loadings in a common solution (2-ethylhexanoic acid and acetonitrile), which was subsequently introduced into the flame. These “black titania” plasmonic nanoparticles exhibit excellent stability under ambient conditions and exceptional photoactivity under UV and visible light, making them promising for photocatalytic and solar energy applications [212]. Furthermore, Solakidou et al. [68] employing single-nozzle and double-nozzle FSP (reviewed in Section 2.3), synthesized {plasmonic metal–TiO_2_ nanohybrids}, specifically Au/TiO_2_ and Ag/TiO_2_. These nanohybrids, leveraging the LSPR phenomenon and energetic hot electrons, demonstrated exceptional efficiency in H_2_ production from photocatalytic reactions involving H_2_O/methanol [68].

Biodegradable silver carriers in polymer coatings improve antimicrobial surfaces compared with traditional inert glass or silica-based silver agents. The enhanced silver activity on calcium phosphate Ag/Ca_3_(PO_4_)_2_ as synthesized by Loher et al. [213] is linked to microorganism uptake of nutrition minerals, facilitating timely silver release. This effect results in up to a three-fold increase in bacteria-killing efficiency, notably for *E. coli*, and reduces silver consumption. These silver carriers have potential applications in food and pharmaceutical production, as well as domestic settings, addressing environmental concerns and offering self-sterilizing surfaces. In healthcare, they exhibit high efficacy against clinically important microorganisms, including *C. albicans* and *P. aeruginosa* [213].

Hybrid Ag/Fe_2_O_3_ nanoparticles with Janus or dumbbell-like morphology were prepared and coated with a nanothin SiO_2_ layer using scalable flame aerosol technology [204]. This process involved the utilization of silica vapor through ring deposition. The plasmon absorption band of Ag became more pronounced with increasing Ag content and size, and the Fe_2_O_3_ component enabled magnetic manipulation in aqueous suspensions. The SiO_2_ coating significantly reduced the release of Ag^+^ ions, rendering the nanoparticles biocompatible for bioimaging. SiO_2_ coating also minimized agglomeration in contrast to uncoated Ag/Fe_2_O_3_ particles, which tended to flocculate and settle quickly (see Figure 25b–d). These hybrid {plasmonic-magnetic} nanoparticles were successfully used as bioprobes, labeled and bound to the membranes of tagged Raji and HeLa cells and detected under dark-field illumination. They combine the benefits of Fe_2_O_3_ (particle stability), Ag (plasmonic properties), and SiO_2_ (inert surface) while overcoming their individual limitations [204]. Similarly, employing the same method, Sotiriou et al. [203] developed biocompatible hybrid nanoaggregates (<100 nm) of Au/Fe_2_O_3_ nanoparticles with a nanothin SiO_2_ coating, suitable for in vivo tumor photothermal ablation via near-IR laser irradiation (see Figure 25a). These nanoparticles exhibit controlled interparticle distances, enabling enhanced photothermal effects. They can effectively ablate tumors in vitro and have potential in in vivo applications. Surface biofunctionalization ensures dispersion in aqueous solutions, while the SiO_2_ shell preserves superparamagnetism and allows for MRI detection [203].

### 3.6. Nanofilm Engineering by FSP

Nanofilms, also known as thin films or particle films, are precisely engineered ultrathin layers with nanometer-scale thicknesses that display distinct properties and applications. Nanofilms find diverse utilization in various scientific and technological realms, including optics, electronics, and materials science. Their applications encompass optical coatings for enhancing light transmission or reflection, functional layers in electronic devices, protective coatings against corrosion and wear, templates for the fabrication of nanostructures, and can be efficiently utilized in challenging chemical processes of immediate technological importance, such as H_2_ production and CO_2_ reduction, to name a few. The precise control over their thickness and composition allows for tailoring the properties of nanofilms to meet specific requirements, making them indispensable in the advancement of nanotechnology and various cutting-edge technologies. Nanofilms are typically fabricated using various synthesis methods: physical vapor deposition (PVD) [216], chemical vapor deposition (CVD) [217], sol-gel deposition [218], layer-by-layer assembly (LbL) [219], spin coating [220], Langmuir–Blodgett (LB) technique [221], and chemical bath deposition (CBD) [222]. Subsequently, we shall revisit some foundational studies and present recent advancements in the fabrication of nanostructured films through the utilization of FSP methodology (parameters specified in Table 10).

In the mid-2000s, Mädler and coauthors [92] synthesized both undoped and Pt-doped tin dioxide nanoparticles in a single-step process. The aerosol generated via the dry FSP approach was directly subjected to in situ thermophoretic deposition onto interdigitated Pt-electrodes, yielding a porous film with a regulated thickness within the sensor’s active region. A consistent tin oxide grain dimension (10 nm) and elevated film porosity (98%) were maintained across all film thicknesses, spanning from 9 to 40 µm, by adjusting deposition durations. Despite varying deposition times, the tin oxide grain size remained at 10 nm with a film porosity of 98%. Platinum doping had no impact on the SnO_2_ grain size or film structure. These sensors showcased exceptional carbon monoxide (CO) detection capabilities, especially at 350 °C, with high repeatability and sensitivity. In situ platinum doping improved sensor performance, while adjusting film thickness provided a means to modify sensor resistance.

**Figure 28:**
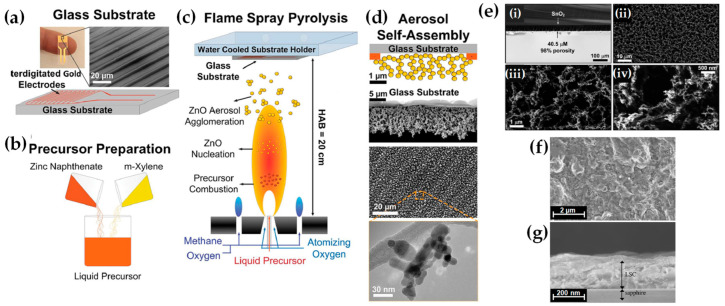
(**a**) A photodetector substrate and (**b**,**c**) the synthesis via flame spray pyrolysis and subsequent aerosol self-assembly, resulting in (**d**) ultraporous films composed of electron-depleted ZnO nanoparticles at a 20 cm height above the burner (HAB). These films are uniformly structured and predominantly consist of spherical particles with an average TEM size of 19 nm (**d**). Reproduced with permission from ref. [93] Copyright 2015 Wiley-VCH. (**e**) SEM cross-sectional (i) and top views (ii–iv) of a SnO_2_ nanoparticle film, aerosol-deposited with a precursor concentration of 0.5 mol/L, for a duration of 4 min. Reproduced with permission from ref. [98] Copyright 2012 Wiley-VCH. (**f**,**g**) SEM images of an as-deposited LSC thin film produced by flame spray deposition on a sapphire substrate: (**f**) top view and (**g**) cross-sectional view. The film was deposited over a duration of 15 min at 200 °C during the flame spray deposition process. Reprinted from [223], with permission from Elsevier.

La_0.6_Sr_0.4_CoO_3−δ_ (LSC) thin films, fabricated by Karageorgakis et al. [223] on sapphire substrates and ceria-gadolinia oxide (CGO) pellets using flame spray deposition at 200 °C, were initially amorphous and dense (refer to Figure 28f,g). The precursor solution was formulated by dissolving lanthanum nitrate, strontium chloride, and cobalt nitrate in N,N-dimethylformamide, resulting in a concentration of 0.006 M. Post-annealed at temperatures above 600 °C, they exhibited nanocrystalline grains, with sizes from 21.4 ± 5 nm at 700 °C to 124 ± 22 nm at 900 °C. Early annealing stages introduced 10–23% porosity, which reduced upon further thermal treatments. Electrochemical tests underscored the necessity of crystallinity for optimal LSC film performance [223]. Films annealed at 400 °C had high area-specific resistance (ASR) values (5.8 Ω cm^2^) at 600 °C, whereas those annealed at 700 °C achieved a lower ASR of 0.96 Ω cm^2^. However, annealing beyond 700 °C raised the ASR due to grain enlargement. Over a 5-day evaluation at 550 °C, ASR degradation was 3.9% for 700 °C-annealed films and 7% for 800 °C-annealed ones, indicating that 700 °C-annealed LSC films hold promise for micro-solid oxide fuel cells (SOFC) applications [223].

**Table 10:** The literature summary of FSP characteristics/conditions for aerosol nanofilm deposition.

Nano-Film	FSP Thin Film Configuration	Precursor(s)	Solvent(s)	Molarity (mol L^−1^)	Pilot Flame O_2_/CH_4_ (L min^−1^)	Precursor Flow (mL min^−1^)	Oxygen Flow (L min^−1^)	Remarks	Size (nm)	Ref.
Pt/SnO_2_	The open flame FSP reactor was used to directly deposit pa- rticles onto a sensor (alumina) substrate.	Sn(II) 2-ethylhexanoic acid, Pt acetylacetonate	Toluene	0.5	3.2/1.5	5	5	Sensor response = 8 for 50 ppm CO at 350 °C	~10	[92]
ZnO, TiO_2_	ZnO/TiO_2_ films are deposited on amorphous silica substrates of 10 mm × 10 mm.	Zn acetate dissolved in deionized water./ Precipitation of hydrous Ti oxide by add- ing ammonia to TiOCl_2_ solution. The preci- pitate is filtered, washed, mixed with trie-thanolamine, heated at 200 °C for 2 h, and then diluted with deionized water.		35 g L^−1^/ 50 g L^−1^	C_2_H_2_:O_2_ = (1:1)	16,000		Remazol brilliant blue dye is degraded by ZnO and TiO_2_. Reactivity: ZnO (90% after 60 min) > TiO_2_.	100–500	[96]
La_0.6_Sr_0.4_ CoO_3−δ_ (LSC)	The LSC thin films have been flame spray deposited on sapphire substrates and ceria-gadolinia oxide pellets.	La nitrate, Sr chloride, Co nitrate	N,N-dimethylformamide	0.006	7/2	0.5	20	Area-specific resistance (ASR) of 0.96 Ω cm^2^ at 600 °C. ASR degraded 3.9% in 5 days at 550 °C in the air.	38	[223]
SnO_2_, TiO_2_	Particles deposited by orthogonal FSP aerosol impingement on a 20 cm temperature-controlled substrate (alumina) holder.	Sn(II)-ethylhexanoate or Ti(VI) isopropoxide	Xylene	0.1–0.5	3.2/1.5	5	5	Introduces a theoretical framework for calculating FSP deposition rates based on the thermal gradient (ΔT) between aerosol and substrate interfaces.	SnO_2_: 10, TiO_2_: <50	[98]
Ag/PMMA-coated glass substrates	FSP deposition of Ag particles which are sub sequently embedded by spin-coating to form polymer nanocomposite films.	Ag acetate	2-ethylhexanoic acid and acetonitrile (1:1)	0.4	3.2/1.5	3	6	Scalable manufacturing of <1 μm polymeric nanocomposite films with high conductivity (5 × 10^4^ S cm^−1^), even under repeated bending.	<100	[224]
ZnO ultraporous networks	FSP deposition of ZnO nanoparticle films onto glass substrates with Au interdigitated electrodes.	Zn naphthenate	Xylene	0.3	2/1.2	5	7	Photodetector performance achieved the highest reported photo-to-dark-current ratio (3.4 × 10^5^) at very low light intensity (0.1 mW cm^−2^).	19	[93]
BiVO_4_ photoanodes	FSP and thermophoretic deposition of the nanoparticle aerosols directly on FTO glass substrates.	Bi(III) 2-ethylhe- xanoate, vanadyl naphthenate	2-ethylhexanoic acid and toluene (1:1)	0.1	2/1.8	5	5	BiVO_4_ photoanodes with 46% film porosity and 400 nm thickness, yielding significant photocurrent densities for sulfite (1.5 mA cm^−2^) and water (1.0 mA cm^−2^) oxidation.	<400	[225,226,227]
Pt-Ru	Flame aerosol synthesis of Pt-Ru deposited dire-ctly as a thin layer on the gas diffusion layer.	Ru(III) acetylacetonate, Pt(II) acetylacetonate	Isooctane and tetrahydrofuran (4:1)			0.5	2.2	Pt-Ru anode electrodes for the direct methanol fuel cell (DMFC). Flame-catalyst surpasses 10%Pt–Ru/C E-TEK commercially with 60% higher activity at 0.4 V for methanol oxidation at 90 °C.	10.3	[228]
Core-shell Ag-SiO_2_	FSP and thermophoretic deposition of plasmonic Ag-SiO_2_ on temperature-controlled cover glasses.	Silver acetate (reflex at 110 °C for 1.5 h), tetraethyl orthosilicate	2-ethylhexanoic acid and acetonitrile		3.2/1.5	5	5	FSP fabrication of SERS sensing substrates. Excellent performance for detecting pesticide residue in orange juices.	12	[229]
Ag/TiO_x_–polymer	Ag/TiO_x_ was FSP and thermophoretically deposited on Si substrates or PDMS polymer layers and mechanically stabilized with ethanol spray annealing.	Silver acetate, Ti(VI) isopropoxide	2-ethylhexanoic acid and acetonitrile (1:1)	0.16		8	5	The optimized polymer nanocomposite films effectively eradicate biofilms upon short, on-demand visible light exposure of 15–90 min with no cytotoxic effects on mammalian cells.	190–600	[230]

As discussed in Section 2.5, the deposition of SnO_2_ and TiO_2_ nanoparticles from FSP reactors onto temperature-controlled substrates is influenced primarily by the temperature difference (ΔT) between the aerosol and substrate [98]. For SnO_2_, the deposition rate increased linearly with time and was dominated by thermophoresis up to ΔT of 121 K. This mechanism was linked to the rapid formation of large agglomerates. The primary particle concentration of a SnO_2_ aerosol aligned with its mobility size distribution, accounting for agglomerate structure and size (see Figure 28e). The study also provided an entrainment rate for surrounding air. Findings suggest that nanoparticle deposition rates for similar aerosols can be predicted using initial precursor concentrations, making the model applicable to various nozzle designs and FSP settings [98].

Polymeric nanocomposite films, characterized by nanoparticle-specific attributes, are gaining attention for potential use in advanced functional materials and miniaturized devices tailored for electronic and biomedical sectors. Blattmann and his colleagues [224] reported the method of flame aerosol deposition of metallic nanosilver onto water-cooled either uncoated or polymer-coated (poly methyl methacrylate, PMMA) glass substrates. Subsequent polymer spin coating resulted in the swift creation of flexible, free-standing, electrically conductive nanocomposite films. The electrical conductivity of these films is ascertained during fabrication and is influenced by the substrate composition and the duration of Ag deposition. Consequently, these researchers have fabricated thin (less than 500 nm) and flexible nanocomposite films with conductivities comparable to metals, such as a value of 5 × 10^4^ S cm^−1^, which is maintained even upon repetitive bending [224]. In a recent publication from Nasiri et al. [93], they introduced a sophisticated hierarchical morphology for ultraviolet (UV) photodetectors. This structure offers superior selectivity and has demonstrated an unprecedented high mA photocurrent response even at minimal ultraviolet light intensities, accompanied by nA dark currents. The study elucidates a swift singular-step FSP synthesis and self-assembly process to produce transparent ultraporous films. The FSP system was employed to directly deposit ZnO nanoparticle films onto glass substrates with Au-interdigitated electrodes (see Figure 28a–d) [93]. A solution of 0.3 M zinc naphthenate in xylene was supplied at 5 mL min^−1^, dispersed using 7 L min^−1^ O_2_, and ignited with a premixed O_2_/CH_4_ flame. Substrates, situated 20 cm above the burner, were kept below 150 °C using a water-cooled holder. Through optimization of the film structure, an absorption rate exceeding 80% of incoming ultraviolet radiation while maintaining a transmission rate approaching 90% for visible light was achieved. Further examination of the photodetector’s efficacy, especially under minimal light intensity conditions (0.1 mW cm^−2^), yielded a significant photo-to-dark current ratio of 3.4 × 10^5^. Such results underscore the potential of this architecture, offering a versatile and scalable technological framework for the swift, cost-efficient production and assimilation of ultraviolet photodetectors in CMOS (complementary metal oxide semiconductor)-compatible mobile apparatuses [93].

**Figure 29:**
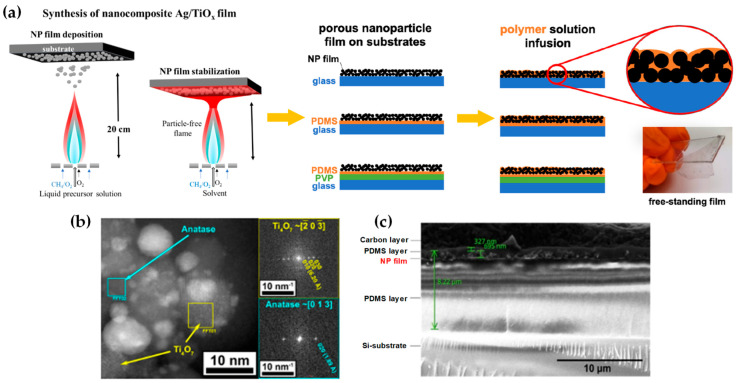
(**a**) Using flame aerosol deposition and mechanical stabilization (in situ annealing), nanostructured films are produced in a single step. Nanoparticles formed in the flame are thermophoretically deposited onto substrates like Si, glass, or polymer-coated materials. By infusing a polymer, such as through spin coating, these films gain mechanical stability. Incorporating a sacrificial layer, like polyvinylpyrrolidone (PVP), enables the creation of free-standing polymer nanocomposite films. (**b**) HRTEM image of Ag/TiO_x_ nanoparticles illustrates the pronounced crystallinity of both Ag and TiO_x_ within the synthesized nanoparticles. (**c**) Cross-sectional scanning electron microscopy (SEM) representation of the Ag/TiO_x_ polymer nanocomposite film with a deposition time (t_d_) of 15 s and a composition of 50% Ag/Ti. Reprinted from [230].

Li et al. [229] have presented FSP-based engineering SERS sensing substrates. Plasmonic Ag-SiO_2_ nanoaggregates were deposited on glass substrates, yielding consistent and efficient SERS sensing films. These substrates exhibited remarkable uniformity, sensitivity, and batch consistency [226]. Compared with other commercial alternatives, these substrates achieved similar performance but at a lower cost, suggesting their broad applicability. Their work’s standout feature was the use of a real-world sample, fresh orange juice, demonstrating SERS’s potential for on-site, label-free detection of contaminants in specific pH conditions using minimal samples [229]. Bletsa et al. [230] synthesized a visible-light-responsive Ag/TiO_x_ coating optimized for application on medical devices, aiming for targeted biofilm eradication. Fabricated via the direct deposition of flame aerosol onto substrates, this methodology facilitated simultaneous nanoparticle generation and film formation (see Figure 29). The outcome is a porous nano-thin suboxide Ag/TiO_x_ particulate layer, which exhibited visible-light photocatalytic activity within, enabling the generation of superoxide radicals adept at biofilm disruption [227]. Various Ag/Ti (Ag acetate/Ti(IV) isopropoxide in a 1:1 mixture of 2-ethylhexanoic acid and acetonitrile) weight concentrations, ranging from 5% to 50%, were produced [230]. The deposition varied in duration from 5 to 60 s, impacting film thickness. These films were mechanically stabilized via in situ annealing with an ethanol spray (as shown in Figure 29a)**.** Notably, the biocompatible surface of the coatings, derived from polydimethylsiloxane (PDMS), exhibited no cytotoxic effects on mammalian cells. Emphasizing the eradication of pre-existing biofilms and evaluating antibiofilm activity on nanoparticle coatings, this study underscores the promise and durability of such coatings for medical device applications [230].

### 3.7. FSP Nanostructures for Sensing Applications

In the realm of nanotechnology and sensor technology, extensive reviews have been conducted to assess the efficacy of flame-made particles as viable materials for sensor applications. Recent scientific literature has featured comprehensive reviews aiming to assess the potential of FSP in this context. Kemmler and colleagues [231], in particular, have embarked upon an investigation to evaluate the utility of FSP as an inherently more efficient approach for sensor development. FSP has been employed to synthesize a diverse array of semiconducting metal oxide (SMOX) materials, along with the deposition of sensitive gas films onto substrates. Additionally, Tricoli et al. [232] conducted a comprehensive investigation into the methodologies utilized in gas sensor fabrication, including FSP and other relevant techniques. Righettoni and coauthors [233] offer an evaluation of the capabilities of metal oxide chemi-resistive gas sensors concerning their utility in breath analysis and monitoring. Simultaneously, Güntner et al. [234] delineate the prevailing obstacles and strategic approaches related to breath sensors for health monitoring. A concise review authored by Sheng et al. [235] presents a catalog of catalysts and sensor materials synthesized through flame-based methods. Ultimately, the most recent review by Pokhrel and Mädler [40], encompassing the period until 2020, highlights significant instances of FSP nanostructures deployed in sensor applications. Hereinunder in the present compilation, we discuss an up-to-date record of studies conducted subsequent after 2020 (characteristics cataloged in Table 11), thereby elucidating the latest advances and research in the domain of FSP-based sensors.

**Figure 30:**
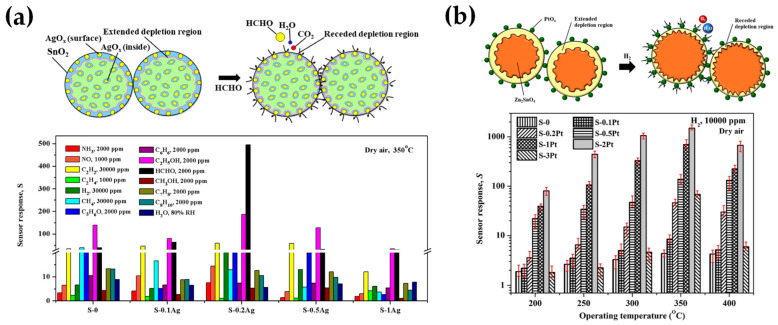
(**a**) HCHO-sensing models of SnO_2_ nanoparticles with AgO_x_-doping at a moderate content (0.2 wt%), and the response histogram of the AgO_x_-doped SnO_2_ sensors with different Ag contents (S-0 to S-1Ag) for toxic gases (NH_3_ and NO), flammable gases (C_2_H_2_, C_2_H_4_, H_2_ and CH_4_), and VOCs (C_3_H_6_O, C_6_H_6_, C_2_H_5_OH, HCHO, CH_3_OH, C_7_H_8,_ and C_8_H_10_) at 350 °C. Reprinted from [236], with permission from Elsevier. (**b**) H_2_-sensing models of PtO_x_-loaded Zn_2_SnO_4_ nanoparticles with optimum Pt contents, and the sensor response to 10,000 ppm H_2_ of 0–3 wt% PtO_x_-loaded Zn_2_SnO_4_ (S-0 to S-3Pt) as a function of operating temperature in the range of 200–400 °C. Reprinted from [237], with permission from Elsevier.

Flame-synthesized AgO_x_-doped SnO_2_ nanoparticles were assessed for formaldehyde (HCHO) detection [236]. Following FSP, both undoped and AgO_x_-doped SnO_2_ nanostructures, ranging from 0.1 to 1 wt%, were homogenously dispersed within an organic paste comprising ethyl cellulose and α-terpineol. This dispersion process was undertaken with the intention of fabricating the sensing films. An optimal 0.2 wt% Ag content yielded a strong response to HCHO, with high selectivity, stability, and minimal sensitivity to environmental conditions (refer to Figure 30a). The improved performance is attributed to p-n heterojunctions and catalytic effects between AgO_x_ and SnO_2_ [236].

Kaewsiri et al. [237] synthesized PtO_x_-loaded Zn_2_SnO_4_ nanoparticles with varying Pt concentrations (0–3 wt%) for hydrogen (H_2_) sensing using single-nozzle FSP. The structural analysis confirmed the uniform attachment of 1–3 nm PtO_x_ nanoparticles to 5–15 nm cubic Zn_2_SnO_4_ nanoparticles. Sensing layers were produced via spin coating and evaluated for their response to environmental gases and VOCs at 200–400 °C under dry ambient conditions. The sensor with an optimal 2 wt% Pt content exhibited remarkable H_2_ selectivity, with a response of 1500.4 and a fast response time of ∼3.4 s to 10,000 ppm H_2_ at 350 °C (see Figure 30b). This sensor showed low sensitivity to humidity, high stability, and reproducibility. These outcomes were attributed to the PtO_x_-Zn_2_SnO_4_ heterointerface influence on spillover mechanisms [237].

Detection of ammonia (NH_3_) is crucial for the optimal modulation of the selective catalytic reduction (SCR) system. In the study of Xiao and colleagues [127], an NH_3_ sensor was developed using a LaCo_1–x_Fe_x_O_3_ sensing electrode, yttria-stabilized zirconia (YSZ) electrolyte, and a platinum (Pt) reference electrode. The LaCo_1–x_Fe_x_O_3_ materials were synthesized via FSP, with Fe substitution at the B-site to enhance sensing efficacy. Within a concentration range of 20–70 ppm at 475 °C, the sensor with the LaCo_0.9_Fe_0.1_O_3_ electrode showcased the highest sensitivity to ammonia compared with other variants. The sensor demonstrated a segmented linear relationship between response value changes and logarithmic ammonia concentration, with sensitivities of 17.52 and 87.22 mV/decade within specified ranges. The exemplary sensing attributes are attributed to oxygen vacancies and the electrocatalytic efficiency of the perovskite electrode. The sensor also exhibited robust selectivity against various gases and maintained stability amid environmental fluctuations. A 22-day test revealed minimal response variance, and the study also touched upon the sensor’s underlying mixed potential sensing mechanism [127]. Moreover, nanoparticles of SnO_2_ doped with 0.1–2 wt% Nb were synthesized for the first time using FSP [238]. Structural analyses, including XRD and electron microscopy, confirmed Nb^5+^ integration within the lattice of nanocrystalline tetragonal SnO_2_ particles (5–15 nm). Sensing layers, created through spin coating, were evaluated against 0.05–1 vol% acetylene (C_2_H_2_) from 200–400 °C in the air. Optimal performance was noted with 0.5 wt% Nb, yielding a sensor response of ∼776 and a rapid 1.1-s response time at 350 °C. This Nb-doped SnO_2_ sensor displayed minimal humidity interference, prolonged stability, and heightened C_2_H_2_ selectivity against various compounds. The results underscore the Nb dopant’s catalytic and electronic contributions, positioning the Nb-enhanced SnO_2_ sensor as a viable candidate for C_2_H_2_-sensing applications [238].

**Table 11:** The literature summary of FSP characteristics/conditions and sensing data for FSP-made gas/VOC sensors, based on research post-2020.

Nano- Device	FSP Setup, Sensor Fabrication	Precursor(s)	Solvent(s)	Molarity (mol L^−1^)	Precursor Flow (mL min^−1^)	Oxygen Flow (L min^−1^)	Sensing Performances			Size (nm)	Ref.
							Gas, VOC	Conc. (ppm)/temp.	Response		
AgO_x_–doped SnO_2_	c FSP reactor, spin coating	Sn(II) 2-ethylhexa-noate, silver nitrate	Xylene, acetonitrile	0.5	5	5	HCHO	100, 200, 2000/350 °C	67,107,495	AgO_x_: <3, SnO_2_: 5–20	[236]
PtO_x_–Zn_2_SnO_4_	Open flame FSP reactor, powder pasting, spin coating	Zn(II) acetylacetonate, Sn(II)2-ethylhexanoate/Pt(II) acetylacetonate	Methanol/xylene	0.5	5		H_2_	150, 1000, 10,000/350 °C	30.1, 216.4, 1500.4	PtO_x_: 1–3, Zn_2_SnO_4_: 30–40	[237]
Er-doped SnO_2_	Open flame FSP reactor, powder pasting, spin coating	Sn(II) 2-ethylhexa-noate, Er(III) acetylacetonate hydrate	Ethanol	0.5			C_2_H_4_O	30, 10, 5, 1, 0.05/350 °C	347, 95, 46, 7.6, 1.3	5–20	[239]
LaCo_1–x_Fe_x_O_3_	Enclosed-flame FSP reactor (pilot flame = CH_4_: 1.25, O_2_: 3.2, Ar: 5.0 L min^−1^), powder pasting, spin coating	La nitrate, Co nitrate, Fe nitrate	Methanol	0.3		5	NH_3_	20–70/475 °C	Sensitivity: 87.22 mV/decade	10	[127]
Nb-doped SnO_2_	Open flame FSP reactor, powder pasting, spin coating	Sn(II) 2-ethylhexa-noate, Nb(V) ethoxide	Absolute ethanol	0.5	5	5	C_2_H_2_	1000/350 °C	776	5–15	[238]
Graphene/Rh–doped SnO_2_	Open flame FSP reactor, electrolytic exfoliation, powder pasting, spin coating	Rh(III) acetylacetonate, Sn(II) 2-ethylhexanoate	Xylene				H_2_S	10/350 °C	439	5–20	[240]
Ga_2_O_3_/Nb	Open flame FSP reactor with chamber, post-annealing	Potassium acetylacetonate, gallium nitrate in dichloromethane/Nb(V) 2-ethylhexanoate	Toluene	0.2	3	1.5	C_3_H_6_O, H_2_, CH_4_	20/470 °C, 20/500 °C, 10,000/500 °C	9.8, 2.5, 4	<3	[241]
La_2_O_3_–WO_3_	Open flame FSP reactor, powder pasting, spin coating	Ammonium (meta) tungstate hydrate, La(III) nitrate hydrate	Diethylene glycol monobutyl ether, ethanol	0.02	5	5	NO_2_	5, 1, 0.05/ 150 °C	7213.6, 1045.3, 15.2	La_2_O_3_: 1–2, WO_3_: 5–20	[242]
La_1–x_FeO_3–δ_ with A-site deficiency	Enclosed-flame FSP reactor (pilot flame = CH_4_: 1.25, O_2_: 3.2, Ar: 5.0 L min^−1^), powder pasting, screen-printing	La nitrate, Fe nitrate	Absolute ethyl alcohol			5	CO_2_	10%/425 °C	3.38	<10	[128]
Zn_2_SnO_4_	Open flame FSP reactor, powder pasting, spin coating	Zn(II) acetylacetonate, Sn(II) 2-ethylhexanoate	Methanol, xylene	0.5	5	5	HCOOH	1000, 50, 20/300 °C	1829, 41.65, 10	5–25	[243]

The same research team [240] synthesized Rh-doped SnO_2_/electrochemically exfoliated graphene hybrid materials via flame processes and conducted a comprehensive study on their gas sensing capabilities, particularly toward hydrogen sulfide (H_2_S). Structural analyses showcased an enhanced surface area due to the integration of graphene sheets on Rh-substituted SnO_2_ nanoparticles. In systematic evaluations against various gases from 200 to 400 °C in diverse humidity levels (20–80% RH), it was discerned that 0.5 wt% Rh doping substantially improved the H2S sensing of SnO_2_ nanoparticles. Incorporating an optimal 0.5 wt% graphene further optimized this sensing capability. Specifically, the 0.5 wt% graphene-loaded 0.5 wt% Rh-doped SnO_2_ sensor yielded peak responses of ∼439 and a swift 6.5-s response to 10 ppm H_2_S at 350 °C, with pronounced selectivity against other gases. This enhanced H_2_S detection was attributed to the combined effects of catalytic Rh dopants and the active interfaces between graphene and Rh-doped SnO_2_ [240]. Utilizing the FSP method, ultrafine β-Ga_2_O_3_ nanomaterials with a grain size of 6–12 nm and a surface area above 100 m^2^/g are produced [241]. Introducing Nb(V) during synthesis results in its integration into the β-Ga_2_O_3_ lattice but annealing above 800 °C forms a separate GaNbO_4_ phase. The incorporation of Nb(V) into β-Ga_2_O_3_ results in charge compensation within the cationic sublattice, manifesting through a Ga(III) to Ga(I) electronic transition. Despite these alterations, Nb(V) doping does not significantly enhance the gas-sensing properties of β-Ga_2_O_3_. The best sensor response is observed with 1 mol% Nb(V) doped Ga_2_O_3_ after annealing at 900 °C for 24 h. These samples excel in gas and VOC detection (H_2_, CH_4_, acetone) and offer improved long-term performance [241].

**Figure 31:**
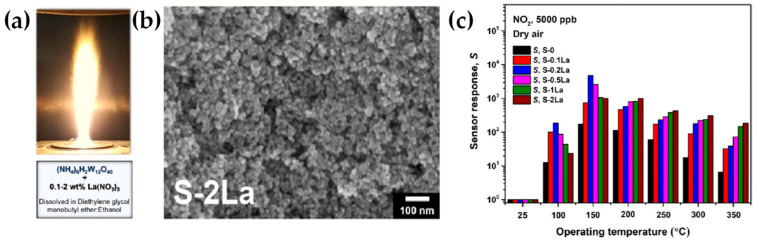
(**a**) Schematic diagrams for FSP synthesis of La_2_O_3_-loaded WO_3_ nanoparticles. (**b**) Areal-view SEM images of S-2La (two spin-coating cycles). (**c**) Sensor responses toward 5000 ppb NO_2_ of 0–2 wt% La_2_O_3_-loaded WO_3_ two-cycle spin-coated films (S-0 to S-2La) in terms of temperature (25–350 °C). Reprinted (adapted) with permission from [242]. Copyright 2023, American Chemical Society.

Recently, La_2_O_3_-loaded WO_3_ nanoparticle films, produced via FSP by Siriwalai et al. [242] using spin-coating techniques, were optimized and analyzed for NO_2_ sensing. Characterization techniques confirmed the presence of 1–2 nm La_2_O_3_ nanoparticles on 5–20 nm WO_3_ nanoparticles. For NO_2_ detection, the optimal performance was achieved with La concentrations up to 0.2 wt% and three spin-coating cycles. This configuration yielded a response of approximately 7213.6 to 5000 ppb NO_2_ with a 31.8 s response time at 150 °C. The sensor also showed high NO_2_ selectivity against other gases and volatile organic compounds, with minimal humidity interference (refer to Figure 31) [242]. Jiao et al. [128] synthesized A-site deficient perovskite nanomaterials, La_1–x_FeO_3–δ_ (0 ≤ x ≤ 0.1), using the FSP method and employed them in chemo-resistive CO_2_ sensors. XRD, TEM, and XPS confirmed the material’s structure and indicated enhanced CO_2_ sensing due to surface oxygen vacancies and Fe^4+^ ions. Among tested materials, La_0.95_FeO_3–δ_ was optimal for detecting 5–15% CO_2_ at 425 °C, showing a response of 3.38 to 10% CO_2_. This sensor effectively distinguished CO_2_ from common vehicular emission gases but showed sensitivity to water vapor. Consequently, La_0.95_FeO_3–δ_ emerges as a promising candidate for CO_2_ monitoring in vehicle exhaust [128].

### 3.8. FSP Nanostructures for Electrocatalytic and Energy Conversion Applications

**Figure 32:**
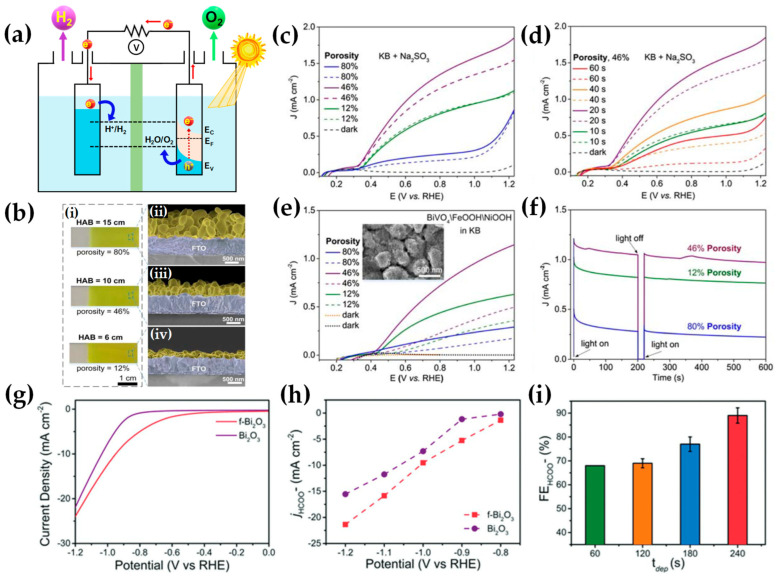
(**a**) Schematic representation of a photoelectrochemical (PEC) cell’s fundamental mechanism, featuring an n-type semiconductor photoanode for oxygen evolution and a platinum sheet photocathode for hydrogen generation in water splitting. (**b**) Optical (i) and SEM (ii–iv) imaging of flame-made BiVO_4_ films on FTO substrates demonstrate variations based on differing HAB settings, with cross sections at (ii) 15 cm, (iii) 10 cm, and (iv) 6 cm HAB corresponding to 60, 20, and 5 s deposition times, ensuring uniform absorbance in each film. (**c**–**f**) BiVO_4_ photoanode PEC metrics were assessed in relation to (**c**) porosity, (**d**) thickness during oxidation in air-saturated 1 M KB (pH 9.3) with 0.2 M Na_2_SO_3_ as a hole scavenger under FTO-side (solid), and BiVO4-side (dashed) illumination. (**e**) PEC responses in 1 M KB (pH 9.3) were measured with (solid) and without (dashed) FeOOH/NiOOH electrocatalyst modification under FTO-side illumination. The SEM inset of (**e**) displays the morphology post-FeOOH/post FeOOH/NiOOH application on a 12% porosity sample. Dark current densities were logged for the 12% porosity sample with (dark dot) and without (yellow dot) electrocatalyst overlay. Measurements were taken via voltammetry at 0.010 V s^−1^ ascending potential in 1 sun equivalent light (AM 1.5 G, 100 mW cm^−2^). (**f**) Longevity trials were conducted on FeOOH/NiOOH-modified samples in 1 M KB (pH 9). Reproduced with permission from ref. [227]. Copyright 2019 Wiley-VCH. (**g**–**i**) CO_2_RR efficacy of f-Bi_2_O_3_ catalysts was evaluated in CO_2_-saturated 0.1 M KHCO_3_. (**g**) f-Bi_2_O_3_ exhibited specific voltammetry profiles at 5 mV s^−1^, with comparative data for filter-collected Bi_2_O_3_. (**h**) The current density for formate production on f-Bi_2_O_3_ and Bi_2_O_3_ varied with potential during CO_2_RR. (**i**) The Faradaic efficiency for formate generation during CO_2_RR correlated with aerosol deposition duration, with measurements taken at −1.2 V vs. RHE. Reproduced with permission from ref. [251]. Copyright 2019 Wiley-VCH.

In the field of electrocatalysis technology, comprehensive evaluations have assessed the effectiveness of particles produced by FSP for electrocatalytic applications. Electrocatalysis involves the facilitation of electrochemical reactions via a catalyst affixed to an electrode, effectively lowering the activation energy barrier. The process encompasses the adsorption of reactants onto the electrode-bound catalyst, followed by an electron exchange during which the reactant is either oxidized or reduced. Subsequent desorption of the reaction product from the catalyst surface allows the transformed species to enter the bulk solution. This sequence enhances the reaction kinetics, with the rate-limiting step being a primary determinant of overall efficiency. Electrocatalysts are selected for their efficiency in specific electron transfer processes, optimizing the conversion of reactants to desired products. In photoelectrochemical (PEC) catalysis (see Figure 32a), light-excited semiconductors produce electron–hole pairs that drive redox reactions at electrode interfaces, aided by electrocatalysts, which lower activation energies and improve reaction kinetics, enabling efficient light-to-chemical energy conversion.

Recent review articles focus on exploring the prospects of flame spray pyrolysis (FSP) in this area. Debecker et al. [244] provide a comprehensive examination of how aerosol processing technologies are revolutionizing the preparation and application of heterogeneous catalysts in various chemical reactions, aiming at both current and future innovations. Similarly, Sheng and colleagues [235] highlight FSP as a key technology for the economical and scalable production of catalytic nanomaterials. Chen and collaborators [94] present FSP as a crucial technique for fabricating nanostructured films, emphasizing its role in advancing sustainable energy technologies, particularly in the context of water catalysis for hydrogen fuel production. In our introductory section, we referenced Tran-Phu et al. [42] who underscore the role of FSP in developing effective (photo) electrocatalysts for sustainable energy storage systems without CO_2_ emissions. Concurrently, John and Tricoli [43] delve into the advancements and future potential of flame-assisted nanofabrication in the context of device integration and industrial applications.

Some studies have selected flame-made cobalt oxide for electrocatalytic processes because it is considered a very promising material for electrochemical reactions. This is due to its high catalytic activity, reasonable electrical conductivity, chemical stability in various environments, high surface area, and versatile morphology. In the study conducted by our laboratory, Belles and coauthors [245] developed flame-made Co_3_O_4_/CoO nanocatalysts for oxygen reduction reaction (ORR) electrodes. In an acidic environment, an electrode composed of 5.2% Pt and 4.8% Co_3_O_4_ exhibited optimal ORR efficiency, achieving a maximum current density of 8.31 mA/cm^2^ and a half-wave potential of 0.66 V. Conversely, in an alkaline setting, an electrode with 0.4% Pt and 9.6% CoO/Co_3_O_4_ demonstrated enhanced performance (J_max_ = 3.5 mA/cm^2^, E_1/2_ = 0.08 V). Pozio et al. [246] and Tran-Phu et al. [247] utilized FSP to manufacture Co_3_O_4_ spinels for use in the oxygen evolution reaction (OER). In addition, Liu and colleagues [248] investigated the electrocatalytic capabilities of Co_3_O_4_ nano-islands, which were directly synthesized and deposited using FSP, as OER catalysts for hydrogen generation on FTO glass substrates.

Tran-Phu and colleagues [227] explored the photooxidation of BiVO_4_ photoanodes with porosities from 12% to 18%, finding that 46% porosity offered the best photocurrent density for sulfite and water oxidation due to optimal charge transport and reaction surface area (Figure 32c–f). Daiyan et al. [249] revealed that subjecting flame-synthesized CuO nanomaterials to mild plasma treatment introduced oxygen vacancies/defects. These defects were closely linked to enhanced performance and endurance in nitrogen oxide reduction reactions (NOxRR), resulting in an ammonium (NH_4_^+^) production rate of 520 μmol cm^−2^ h^−1^ at a cell voltage of 2.2 V in a flow electrolyzer (see Figure 32b). Bismuth (Bi) catalysts are among the best performing candidates for electrochemical reduction of CO_2_ (CO_2_RR) to formate products [250,251]. Tran-Phu et al. [251] produced flame-made fractals Bi_2_O_3_ on carbon fibers, which demonstrated superior catalytic activity for CO_2_RR to formate, with a high mass-specific formate partial current density of −52.2 mA mg^−1^ at −1.2 V (Figure 32g–i).

FSP-produced materials have primarily been utilized as electrodes in fuel cells, batteries, supercapacitors, and dye-sensitized solar cells for energy conversion. Their high-quality metal oxide semiconductors, characterized by purity and crystallinity from the flame method, exhibit desirable chemical stability and electronic traits beneficial for various energy devices. Subsequent paragraphs will offer a succinct overview of notable flame-generated material compositions and device structures.

**Figure 33:**
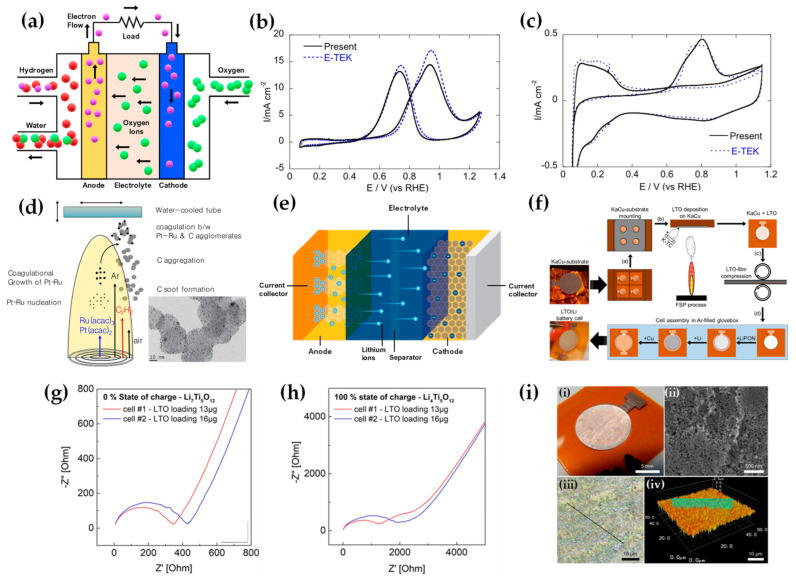
(**a**) Simplified fuel cell’s schematic representation. (**b**) Methanol oxidation and (**c**) CO stripping processes utilizing carbon-supported Pt-Ru catalyst produced via FSP in contrast to the performance of standard E-TEK catalysts. (**d**) Schematic figure depicting the apparatus used for the flame aerosol synthesis of Pt-Ru/C catalysts, with an accompanying TEM image of the synthesized Pt-Ru/C catalysts in the inset. Reprinted from [254], with permission from Elsevier. (**e**) Schematic of a Li-ion battery illustrating its constituent components. Reprinted from [255]. (**f**) The LTO battery cell fabrication involves setting KaCu substrates in an FSP reactor for LTO deposition, followed by compression and transfer to a glove box for sputtering LiPON, lithium, and copper, leading to the final cell assembly. (**g**,**h**) Nyquist diagrams for battery cells featuring fully lithiated rock-salt-type Li_7_Ti_5_O_12_ (**g**) and completely delithiated (initial state) spinel Li_4_Ti_5_O_12_ (**h**) thin films. (**i**) Morphological analysis of compressed LTO thin films includes (i) photographs, (ii) SEM imaging on KaCu substrates, (iii) 2D LSM color and laser imaging, and (iv) 3D surface profiling. Reprinted from [256], with permission from Elsevier.

A fuel cell is an energy conversion device that transforms the chemical energy of a fuel, typically hydrogen, and an oxidant, usually oxygen from the air, into electricity through an electrochemical reaction, bypassing the traditional combustion process [252]. The basic components of a fuel cell include two electrodes—an anode and a cathode—and an intervening electrolyte (refer to Figure 33a). At the anode, H_2_ fuel undergoes a chemical reaction that separates it into positively charged H_2_ ions (protons) and negatively charged electrons. The electrolyte allows only the protons to pass through to the cathode, while the electrons are directed through an external circuit, generating an electric current. On reaching the cathode, the electrons, returning from the external circuit, combine with the protons that have passed through the electrolyte and with O_2_ from the air. This reaction produces water and heat as the only byproducts, making fuel cells an environmentally friendly technology.

Seo et al. [253] used FSP to engineer Ce_1–x_Gd_x_O_2–x/2_ nanoparticles for fuel cell electrolytes, pelletizing and sintering them at 1400 °C for 3 h. They observed that higher temperatures improved ionic conductivity due to increased oxide ion mobility. Their FSP-derived nanoparticles demonstrated the highest conductivity, attributed to larger lattice constants. Lee and colleagues [254] found that incorporating catalysts into fuel cell electrodes, such as FSP-synthesized carbon-supported Pt-Ru (refer to Figure 33d), not only lowers operating temperature but also mitigates CO poisoning. Their study revealed that these flame-made materials exhibited superior methanol oxidation and CO stripping capabilities (see Figure 33b,c), along with competitive electrochemical activity, outperforming commercial catalysts of the same composition in fuel cell performance [254].

The growing importance of electric energy storage in batteries is driven by the rising use of portable devices and electric vehicles, and the need to store renewable energy. Among different technologies, rechargeable Li-ion batteries stand out for their high energy density and lifespan [257]. These batteries consist of an anode, cathode, and a separator soaked in an electrolyte, with Li-ions moving between the anode and cathode during charge and discharge cycles (Figure 33e). Traditionally made through solid-state or sol-gel methods, Li-ion batteries often face challenges like lengthy production times, multiple steps, and impurity presence. Therefore, numerous studies are focusing on enhancing the fabrication of materials and assembly of electrodes, crucial for battery performance.

Gockeln et al. [256] utilized FSP for the direct fabrication of Li_4_Ti_4_O_12_ (LTO) batteries on flexible polyimide foil (refer to Figure 33f–i). They noticed a potential plateau deviation and shortening at higher current densities, linked to cell polarization under these conditions. Additionally, they explored the performance of in situ-coated nano LTO/C batteries made via a combination of FSP and pressure-based lamination. They observed a stable voltage plateau at discharge rates up to 1 °C, suggesting enhanced charge/discharge reaction kinetics due to improved electronic conductivity in the microstructure.

## 4. Concluding Remarks—Future Perspectives

Flame spray pyrolysis (FSP) is a highly potent technology for the synthesis of advanced nanostructures. While FSP has achieved significant milestones, both in terms of material variety and process optimization, the journey toward its full potential is ongoing. So far, FSP has produced more than 500 different materials, and it is widely used in academia (more than 30 groups working with FSP), adapted by industry (officially Johnson Matthey has announced the use of an FSP facility), and the largest public reactor installed in Spain (http://www.advance-fsp.eu/, accessed on 5 November 2023) producing several kilograms/day. The future prospect lies in designing such high-enthalpy spray combinations using economic metal salts (nitrates, chlorides, acetates) that react with the solvent producing in situ metal alkoxides opening the door toward easily available wide range of inexpensive precursors.

Diligent control of FSP parameters allows for targeted manipulation of nanoparticle characteristics, which is essential for catalyst optimization. These parameters, including flame temperature and precursor feed rate, significantly affect the size and shape of nanoparticles, thereby directly impacting their catalytic activity. Smaller particles, due to their greater surface areas, exhibit higher activity because of the increased number of active sites. FSP also facilitates the synthesis of tailored composite or doped nanoparticles, offering precise control over their composition and dopant distribution, which enhances activity and selectivity. The high temperature conditions in FSP enable the production of high-purity, crystalline nanoparticles, crucial for ensuring catalyst stability and conductivity. Additionally, surface properties, which are vital in electrocatalysis, can be modified through the adjustment of FSP’s quenching rate and atmosphere, influencing surface defects and functionalities. Finally, managing nanoparticle agglomeration through FSP is key; reducing agglomeration helps maintain an effective surface area, thereby improving electrocatalyst performance.

Incorporating automation and advanced process control through the utilization of artificial intelligence (AI) and machine learning promises enhanced consistency and quality in nanoparticle synthesis. These technologies, in conjunction with sophisticated sensors, enable real-time data acquisition and dynamic optimization of the FSP process. Concurrently, the development of in situ characterization techniques during FSP synthesis offers deeper insights into nanostructure formation mechanisms, thereby facilitating a more exacting command over the synthesis process.

Despite this broad range of benefit and potential, the use of FSP synthesis for the integration of nanocomponents in devices still faces some challenges. Among those, the large amount of nanomaterial produced in the aerosol phase, the need to manage large gas volumes, and high temperatures make the integration of flame reactors in clean rooms and standard nanofabrication facilities difficult. The intrinsic very high porosity of aerosol self-assembled nanoparticle films at low temperature makes them fragile and prevents the use of such films in a liquid environment. Sufficient in situ sintering of the layers during deposition is not always possible for materials having low sintering rates and for substrates not able to withstand sufficiently high temperatures.

Innovations are required in the design of flame-assisted synthesis to overcome these challenges and establish it as a standard fabrication route for the integration of nanomaterials in a variety of devices and applications. As a forward-looking summary, several focal points emerge:

(a) *Multifunctionality Enhancement*: As the title of this review suggests, multifunctionality is at the heart of FSP’s premise. Future advancements should emphasize developing processes that can fabricate nanostructures with an even broader range of functionalities, expanding their utility across diverse sectors. There is growing interest in the development of hybrid versions of FSP that incorporate additional physical processes, such as electric fields or ultrasound [258,259]. The integration of an electric field into the FSP process, for instance, could offer enhanced control over particle formation. By applying an electric field, it is possible to influence the charge distribution within the flame, potentially leading to more uniform particle sizes and shapes.
(b) *Technological Synergies*: Harnessing the potential of complementary technologies, like AI and real-time monitoring tools, will trigger the era of “smart” FSP [260]. This would not only ensure the efficient production of desired nanostructures but might also open doors to materials previously deemed unattainable.
(c) *Economic Precursor Development*: As FSP broadens its reach, the use of economical and readily available precursors will be essential. Research geared toward identifying and harnessing such materials will significantly reduce production costs, making FSP-synthesized nanoparticles more accessible.
(d) *Challenge Mitigation*: While the review refers to the advancements, it is vital not to overlook the inherent challenges of FSP, be it in scalability, integration with standard fabrication environments, or ensuring the robustness of the nanostructures. Innovations addressing these challenges head-on will be instrumental in FSP’s widespread adoption.
(e) *Expanding Application Horizons*: While FSP-derived nanoparticles have found their place in various applications, constant research is required to explore untapped potentials. Fields such as renewable energy, biomedicine, and advanced electronics may witness revolutionary products birthed from FSP advancements. Integrating biological components, such as enzymes or other organic molecules, into the FSP process might enable the synthesis of bio-functionalized nanoparticles. These could have specific applications in targeted drug delivery, biosensors, or bio-catalysis.
(f) *Environmental and Safety Concerns*: As with all industrial processes, the environmental impact of FSP, along with safety concerns, will need continuous evaluation. Future iterations of FSP should aim for greener processes, ensuring sustainability.

In summary, FSP technology is poised to play a pivotal role in the next wave of nanotechnological innovations.

## Figures and Tables

**Table 1:** The literature summary on various techniques for synthesizing nanostructures and the criteria for their application.

Criteria	Techniques of Nanomaterials Synthesis									
	Flame Spray Pyrolysis (FSP) [2,7]	Chemical Vapor Deposition (CVD) [8,9]	Sol-Gel Process [10,11]	Hydrothermal Synthesis [12,13]	Plasma- Enhanced CVD [14,15]	Electrospinning [16,17]	Laser Ablation [18,19]	Sputter Deposition [20,21]	Microwave- Assisted Synthesis [22,23]	Atomic Layer Deposition (ALD) [5,6]
**Materials** **Versatility**	High (nanoparticles, thin films)	Moderate (thin films, nanotubes, graphene)	High (nanoparticles, thin films)	High (crystals, nanoparticles)	Moderate (thin films, nanotubes)	Moderate (fibers, nanofibers)	High (nanoparticles, thin films)	Moderate (thin films, coatings)	High (nanoparticles, crystals)	Moderate (ultra-thin films, nanocoatings)
**Cost-** **Effectiveness**	Moderate	High	Low	Moderate	High	Moderate	High	Moderate	Moderate	Moderate
**Scalability**	High	Moderate	High	Low	Moderate	High	Low	High	Moderate	Low
**Synthesis Time**	Short	Long	Long	Long	Long	Short to Moderate	Short	Moderate	Short	Long
**Application Examples**	Catalysts, sensors, energy storage	Semiconductor devices, coatings	Coatings, biomedicine	Crystal growth, mineral synthesis	Semiconductor devices, coatings	Filters, textiles	Medical implants, sensors	Electronics, optics	Pharmaceuticals, chemistry	Electronics, barrier layers
**Control Over Particle Size**	High	High	Moderate	Low	High	High	Moderate	High	High	Exceptional
**Operational Complexity**	Moderate	High	Low	Moderate	High	Moderate	High	Moderate	Moderate	High
**Environmental Impact**	Moderate	High (depends on precursors)	Low	Low	Moderate	Low	Moderate	Moderate	Low	Low
**Required Equipment**	Flame spray reactor, furnace	Vacuum system, reactors	Simple lab equipment	Autoclaves, pressure vessels	Vacuum system, plasma source	Electrospinning setup	Laser system, vacuum chamber	Sputtering system, vacuum chamber	Microwave reactor	Vacuum chamber, gas delivery system
**Precision and Uniformity**	High	Very High	Moderate	Moderate	Very High	High	High	Very High	High	Exceptional
**Safety and Handling**	Moderate risk	High risk (toxic gases)	Low risk	Moderate risk	High risk (toxic gases)	Low risk	High risk (laser hazards)	Moderate risk	Low risk	Moderate risk
**Temperature Range**	High	Very High	Low to Moderate	Moderate to High	Very High	Low to Moderate	High	Low to High	Low to Moderate	Low to Moderate
**Atmosphere** **Control**	Moderate control	Strict control	Not necessary	Strict control	Strict control	Not necessary	Moderate control	Strict control	Not necessary	Strict control

**Table 4:** The literature summary of FSP characteristics/conditions for the production of core-shell nanostructures.

Nano- Structure	FSP Configuration	Ring Characteristics	Precursor(s)	Solvent	Molarity (mol L^−1^)	Pilot Flame O_2_/CH_4_ (L min^−1^)	N_2_ Bubbling Flow Rate (L min^−1^)	Additional N_2_ Flow (L min^−1^)	Ref.
ZnO	1 quartz tube with quench ring	8 nozzles (ID = 1 mm)	Zinc acetylacetonate		1079 ppm	9.87/0.62	0.23		[74]
SiO_2_-coated Al/TiO_2_	2 quartz tubes (5–30 cm and 30 cm) with a stainless steel metal torus ring	pipe diameter = 0.38 cm, ring (ID = 4.5 cm), 16 nozzles (ID = 0.6 cm)	HMDSO, Al tert- butoxide, Ti(IV) isopropoxide	Xylene	1	3.2/1.5	0.6−0.8		[75,77]
SiO_2_-coated nanosilver			HMDSO, Ag-benzoate	2-ethylhexanoic acid/benzonitrile (1:1)	0.5	3.2/1.5	0.8		[78,79,88]
Ag^0^@SiO_2_	1 metallic tube (22 cm) with a stainless steel metal torus ring	diameter 4.3 cm, 12 nozzles (ID = 0.5 cm)	HMDSO, Ag acetate	2-ethylhexanoic acid/acetonitrile (1:1)	0.3–0.5	5/2.5	0.3–3	10–15	[80,81]
rSiO_2_@SiO_2_			HMDSO	Ethanol, xylene		5/2.5	3	5	[82]
CuO_x_@SiO_2_	Coating ring over the flame	8 nozzles	HMDSO, Cu nitrate trihydrate	Anhydrous ethanol	0.5	3.2/1.25	0.8 (Ar)	5 (Ar)	[85]
SiO_2_@YAlO_3_:Nd^3+^	Coating ring with adjustable height over the flame	circular structure and has four tubes oriented to the center of the circle	HMDSO, Y, and Nd nitrate hexahydrate, Al nitrate nonahydrate	Anhydrous ethanol/2-ethylhexanoic acid	0.4	18.06/1.37	–		[86]
SiO_2_-coated Y_2_O_3_:Tb^3+^	2 quartz tubes (5–30 cm and 30 cm) with a stainless steel metal torus ring	pipe diameter = 0.38 cm ring (ID = 4.5 cm), 16 nozzles (ID = 0.6 cm)	HMDSO, Y, and Tb nitrate hexahydrate	Ethanol/2-ethylhexanoic acid	0.5	3.2/1.5	0.5	15	[87]

**Table 8:** The literature summary of characteristics/conditions for FSP-made quantum dots nanomaterials.

Nano- Structure	FSP Configuration	Precursor(s)	Solvent(s)	Molarity (mol L^−1^)	Metal/Si Ratio: X = Metal/ (Metal + Si)	Pilot Flame O_2_/CH_4_ (L min^−1^)	Precursor Flow (mL min^−1^)	Oxygen Flow (L min^−1^)	Size (nm)	SSA (m^2^ g^−1^)	Ref.
ZnO QDs	Open-flame FSP reactor using an air-assist nozzle	Zn acrylate, hexamethyldisiloxane	6 vol% acetic acid in methanol	0.5	0.7				1.5	150	[188]
TiO_2_ QDs	Open-flame FSP reactor	Ti(IV) tetraisopropoxide, hexamethyldisiloxane	Xylene	0.25	0.7	2.5/1.25	1	3.75	2.3		[189]
ZnO QDs	Open-flame FSP reactor	Zn 2-ethylhexanoate, hexamethyldisiloxane	Xylene	0.25	0.1	2.5/1.25	1	3.75	2.1		[189]
SnO_2_ QDs	Open-flame FSP reactor	Sn-2-ethylhexanoate, hexamethyldisiloxane	Xylene	0.25	0.1	2.5/1.25	1	3.75	2.2		[189]
CuO QDs	Open-flame FSP reactor	Cu-2-ethylhexanoate, hexamethyldisiloxane	Xylene	0.25	0.1	2.5/1.25	1	3.75	1.4		[189]
CuO QDs −SrTiO_3_	Metal tube with gap, sheath Ar gas flow (8 L min^−1^)	Cu nitrate trihydrate/ Sr acetate, n-butyl titanate	Ethanol/ acetic acid	0.15	–	1.5/0.75	2–5	5	<5	38–50	
PbS QDs −TiO_2_	Enclosed box under N_2_ purge (O_2_ < 140 ppm)	Pb(II) 2-ethylhexanoate, Ti(IV) isopropoxide, thiophene	Ethylhexanoic acid, THF (2:1)		–	2.4/1.13	5	4–4.5	2	75–85	[142]
CQDs/ TiO_2_–C	Open-flame FSP reactor and post-treatment with Ar/O_2_ atmosphere	Tetrabutyl titanate	Absolute ethanol		–	H_2_/O_2_: 3.3/16.7 (L min^−1^)	5	5	<5		[190]
Bi_2_WO_6_ QDs	Ope-flame FSP reactor	W hexacarbonyl/ Bi neodecanoate	Tetrahydrofuran /xylene	0.3	–	3.2/1.5	5	5	2.2		[191]

## Data Availability

Not applicable.

## References

[1] Meierhofer F., Fritsching U. (2021). Synthesis of Metal Oxide Nanoparticles in Flame Sprays: Review on Process Technology, Modeling, and Diagnostics. Energy Fuels.

[2] Teoh W.Y., Amal R., Mädler L. (2010). Flame Spray Pyrolysis: An Enabling Technology for Nanoparticles Design and Fabrication. Nanoscale.

[3] Ulrich G.D. (1984). Special Report. Chem. Eng. News Arch..

[4] Liu S., Mohammadi M.M., Swihart M.T. (2021). Fundamentals and Recent Applications of Catalyst Synthesis Using Flame Aerosol Technology. Chem. Eng. J..

[5] Leskelä M., Ritala M. (2002). Atomic Layer Deposition (ALD): From Precursors to Thin Film Structures. Thin Solid Films.

[6] George S.M. (2010). Atomic Layer Deposition: An Overview. Chem. Rev..

[7] Karthikeyan J., Berndt C.C., Tikkanen J., Wang J.Y., King A.H., Herman H. (1997). Nanomaterial Powders and Deposits Prepared by Flame Spray Processing of Liquid Precursors. Nanostructured Mater..

[8] Cai Z., Liu B., Zou X., Cheng H.-M. (2018). Chemical Vapor Deposition Growth and Applications of Two-Dimensional Materials and Their Heterostructures. Chem. Rev..

[9] Sun L., Yuan G., Gao L., Yang J., Chhowalla M., Gharahcheshmeh M.H., Gleason K.K., Choi Y.S., Hong B.H., Liu Z. (2021). Chemical Vapour Deposition. Nat. Rev. Methods Primers.

[10] Hench L.L., West J.K. (1990). The Sol-Gel Process. Chem. Rev..

[11] Danks A.E., Hall S.R., Schnepp Z.J.M.H. (2016). The Evolution of ‘Sol–Gel’ Chemistry as a Technique for Materials Synthesis. Mater. Horiz..

[12] Byrappa K., Adschiri T. (2007). Hydrothermal Technology for Nanotechnology. Prog. Cryst. Growth Charact. Mater..

[13] Rabenau A. (1985). The Role of Hydrothermal Synthesis in Preparative Chemistry. Angew. Chem. Int. Ed. Engl..

[14] Hess D.W. (1984). Plasma-enhanced CVD: Oxides, Nitrides, Transition Metals, and Transition Metal Silicides. J. Vac. Sci. Technol. A.

[15] Vasudev M.C., Anderson K.D., Bunning T.J., Tsukruk V.V., Naik R.R. (2013). Exploration of Plasma-Enhanced Chemical Vapor Deposition as a Method for Thin-Film Fabrication with Biological Applications. ACS Appl. Mater. Interfaces.

[16] Bhardwaj N., Kundu S.C. (2010). Electrospinning: A Fascinating Fiber Fabrication Technique. Biotechnol. Adv..

[17] Teo W.E., Ramakrishna S. (2006). A Review on Electrospinning Design and Nanofibre Assemblies. Nanotechnology.

[18] Zeng H., Du X.-W., Singh S.C., Kulinich S.A., Yang S., He J., Cai W. (2012). Nanomaterials via Laser Ablation/Irradiation in Liquid: A Review. Adv. Funct. Mater..

[19] Kim M., Osone S., Kim T., Higashi H., Seto T. (2017). Synthesis of Nanoparticles by Laser Ablation: A Review. KONA Powder Part J..

[20] Jansson U., Lewin E. (2013). Sputter Deposition of Transition-Metal Carbide Films—A Critical Review from a Chemical Perspective. Thin Solid Films.

[21] Thornton J.A. (1986). The Microstructure of Sputter-deposited Coatings. J. Vac. Sci. Technol. A.

[22] Nüchter M., Ondruschka B., Bonrath W., Gum A. (2004). Microwave Assisted Synthesis—A Critical Technology Overview. Green Chem..

[23] Lidström P., Tierney J., Wathey B., Westman J. (2001). Microwave Assisted Organic Synthesis—A Review. Tetrahedron.

[24] Malekzadeh M., Swihart M.T. (2021). Vapor-Phase Production of Nanomaterials. Chem. Soc. Rev..

[25] Rsanchez Technology Readiness Assessment Guide—DOE Directives, Guidance, and Delegations. https://www.directives.doe.gov/directives-documents/400-series/0413.3-EGuide-04a.

[26] Workie A.B., Ningsih H.S., Shih S.-J. (2023). An Comprehensive Review on the Spray Pyrolysis Technique: Historical Context, Operational Factors, Classifications, and Product Applications. J. Anal. Appl. Pyrolysis.

[27] Teoh W. (2013). A Perspective on the Flame Spray Synthesis of Photocatalyst Nanoparticles. Materials.

[28] Strobel R., Pratsinis S.E. (2007). Flame Aerosol Synthesis of Smart Nanostructured Materials. J. Mater. Chem..

[29] Haynes W.M., CRC (2014). Handbook of Chemistry and Physics.

[30] Sokolowski M., Sokolowska A., Michalski A., Gokieli B. (1977). The “in-Flame-Reaction” Method for Al_2_O_3_ Aerosol Formation. J. Aerosol Sci..

[31] Bickmore C.R., Waldner K.F., Treadwell D.R., Laine R.M. (1996). Ultrafine Spinel Powders by Flame Spray Pyrolysis of a Magnesium Aluminum Double Alkoxide. J. Am. Ceram. Soc..

[32] Tikkanen J., Gross K.A., Berndt C.C., Pitkänen V., Keskinen J., Raghu S., Rajala M., Karthikeyan J. (1997). Characteristics of the Liquid Flame Spray Process. Surf. Coat. Technol..

[33] Mädler L., Kammler H.K., Mueller R., Pratsinis S.E. (2002). Controlled Synthesis of Nanostructured Particles by Flame Spray Pyrolysis. J. Aerosol Sci..

[34] Mädler L. (2004). Liquid-Fed Aerosol Reactors for One-Step Synthesis of Nano-Structured Particles. KONA Powder Part. J..

[35] Camenzind A., Caseri W.R., Pratsinis S.E. (2010). Flame-Made Nanoparticles for Nanocomposites. Nano Today.

[36] Sotiriou G.A. (2013). Biomedical Applications of Multifunctional Plasmonic Nanoparticles. WIREs Nanomed. Nanobiotechnol..

[37] Koirala R., Pratsinis S.E., Baiker A. (2016). Synthesis of Catalytic Materials in Flames: Opportunities and Challenges. Chem. Soc. Rev..

[38] Li S., Ren Y., Biswas P., Tse S.D. (2016). Flame Aerosol Synthesis of Nanostructured Materials and Functional Devices: Processing, Modeling, and Diagnostics. Prog. Energy Combust. Sci..

[39] Schneider F., Suleiman S., Menser J., Borukhovich E., Wlokas I., Kempf A., Wiggers H., Schulz C. (2019). SpraySyn—A Standardized Burner Configuration for Nanoparticle Synthesis in Spray Flames. Rev. Sci. Instrum..

[40] Pokhrel S., Mädler L. (2020). Flame-Made Particles for Sensors, Catalysis, and Energy Storage Applications. Energy Fuels.

[41] Venkatesan S., Mitzel J., Wegner K., Costa R., Gazdzicki P., Friedrich K.A. (2022). Nanomaterials and Films for Polymer Electrolyte Membrane Fuel Cells and Solid Oxide Cells by Flame Spray Pyrolysis. Renew. Sustain. Energy Rev..

[42] Tran-Phu T., Daiyan R., Ta X.M.C., Amal R., Tricoli A. (2022). From Stochastic Self-Assembly of Nanoparticles to Nanostructured (Photo)Electrocatalysts for Renewable Power-to-X Applications via Scalable Flame Synthesis. Adv. Funct. Mater..

[43] John A.T., Tricoli A. (2022). Flame Assisted Synthesis of Nanostructures for Device Applications. Adv. Phys. X.

[44] Grass R.N., Stark W.J., Athanassiou E.-K. (2007). Reducing Flame Spray Pyrolysis Method for the Production of Metal, Non-Oxidic, Ceramic and Reduced Metal Oxide Powders and Nano-Powders, European Patent Office. European Patent.

[45] Grass R.N., Albrecht T.F., Krumeich F., Stark W.J. (2007). Large-Scale Preparation of Ceria/Bismuth Metal-Matrix Nano-Composites with a Hardness Comparable to Steel. J. Mater. Chem..

[46] Grass R.N., Stark W.J. (2006). Flame Spray Synthesis under a Non-Oxidizing Atmosphere: Preparation of Metallic Bismuth Nanoparticles and Nanocrystalline Bulk Bismuth Metal. J. Nanopart. Res..

[47] Athanassiou E.K., Grass R.N., Stark W.J. (2006). Large-Scale Production of Carbon-Coated Copper Nanoparticles for Sensor Applications. Nanotechnology.

[48] Grass R.N., Athanassiou E.K., Stark W.J. (2007). Covalently Functionalized Cobalt Nanoparticles as a Platform for Magnetic Separations in Organic Synthesis. Angew. Chem. Int. Ed..

[49] Athanassiou E.K., Grass R.N., Osterwalder N., Stark W.J. (2007). Preparation of Homogeneous, Bulk Nanocrystalline Ni/Mo Alloys with Tripled Vickers Hardness Using Flame-Made Metal Nanoparticles. Chem. Mater..

[50] Athanassiou E.K., Grass R.N., Stark W.J. (2010). One-Step Large Scale Gas Phase Synthesis of Mn^2+^ Doped ZnS Nanoparticles in Reducing Flames. Nanotechnology.

[51] Strobel R., Pratsinis S.E. (2009). Direct Synthesis of Maghemite, Magnetite and Wustite Nanoparticles by Flame Spray Pyrolysis. Adv. Powder Technol..

[52] Kumfer B.M., Shinoda K., Jeyadevan B., Kennedy I.M. (2010). Gas-Phase Flame Synthesis and Properties of Magnetic Iron Oxide Nanoparticles with Reduced Oxidation State. J. Aerosol Sci..

[53] Kumfer B.M., Skeen S.A., Axelbaum R.L. (2008). Soot Inception Limits in Laminar Diffusion Flames with Application to Oxy–Fuel Combustion. Combust. Flame.

[54] Deligiannakis Y., Mantzanis A., Zindrou A., Smykala S., Solakidou M. (2022). Control of Monomeric Vo’s versus Vo Clusters in ZrO_2−x_ for Solar-Light H_2_ Production from H_2_O at High-Yield (millimoles gr^−1^ h^−1^). Sci. Rep..

[55] Zindrou A., Belles L., Solakidou M., Boukos N., Deligiannakis Y. (2023). Non-Graphitized Carbon/Cu_2_O/Cu^0^ Nanohybrids with Improved Stability and Enhanced Photocatalytic H_2_ Production. Sci. Rep..

[56] Zindrou A., Deligiannakis Y. (2023). Quantitative In Situ Monitoring of Cu-Atom Release by Cu_2_O Nanocatalysts under Photocatalytic CO_2_ Reduction Conditions: New Insights into the Photocorrosion Mechanism. Nanomaterials.

[57] Grass R.N., Stark W.J. (2006). Gas Phase Synthesis of Fcc-Cobalt Nanoparticles. J. Mater. Chem..

[58] Psathas P., Zindrou A., Papachristodoulou C., Boukos N., Deligiannakis Y. (2023). In Tandem Control of La-Doping and CuO-Heterojunction on SrTiO_3_ Perovskite by Double-Nozzle Flame Spray Pyrolysis: Selective H_2_ vs. CH_4_ Photocatalytic Production from H_2_O/CH_3_OH. Nanomaterials.

[59] Strobel R., Mädler L., Piacentini M., Maciejewski M., Baiker A., Pratsinis S.E. (2006). Two-Nozzle Flame Synthesis of Pt/Ba/Al_2_O_3_ for NO*_x_* Storage. Chem. Mater..

[60] Minnermann M., Grossmann H.K., Pokhrel S., Thiel K., Hagelin-Weaver H., Bäumer M., Mädler L. (2013). Double Flame Spray Pyrolysis as a Novel Technique to Synthesize Alumina-Supported Cobalt Fischer–Tropsch Catalysts. Catal. Today.

[61] Høj M., Pham D.K., Brorson M., Mädler L., Jensen A.D., Grunwaldt J.-D. (2013). Two-Nozzle Flame Spray Pyrolysis (FSP) Synthesis of CoMo/Al_2_O_3_ Hydrotreating Catalysts. Catal. Lett..

[62] Schubert M., Pokhrel S., Thomé A., Zielasek V., Gesing T.M., Roessner F., Mädler L., Bäumer M. (2016). Highly Active Co–Al_2_O_3_ -Based Catalysts for CO_2_ Methanation with Very Low Platinum Promotion Prepared by Double Flame Spray Pyrolysis. Catal. Sci. Technol..

[63] Horlyck J., Pokhrel S., Lovell E., Bedford N.M., Mädler L., Amal R., Scott J. (2019). Unifying Double Flame Spray Pyrolysis with Lanthanum Doping to Restrict Cobalt–Aluminate Formation in Co/Al_2_O_3_ Catalysts for the Dry Reforming of Methane. Catal. Sci. Technol..

[64] Stahl J., Ilsemann J., Pokhrel S., Schowalter M., Tessarek C., Rosenauer A., Eickhoff M., Bäumer M., Mädler L. (2021). Comparing Co-catalytic Effects of ZrO_x_, SmO_x_, and Pt on CO_x_ Methanation over Co-based Catalysts Prepared by Double Flame Spray Pyrolysis. ChemCatChem.

[65] Gäßler M., Stahl J., Schowalter M., Pokhrel S., Rosenauer A., Mädler L., Güttel R. (2022). The Impact of Support Material of Cobalt-Based Catalysts Prepared by Double Flame Spray Pyrolysis on CO_2_ Methanation Dynamics. ChemCatChem.

[66] Henning D.F., Merkl P., Yun C., Iovino F., Xie L., Mouzourakis E., Moularas C., Deligiannakis Y., Henriques-Normark B., Leifer K. (2019). Luminescent CeO_2_:Eu^3+^ Nanocrystals for Robust in Situ H_2_O_2_ Real-Time Detection in Bacterial Cell Cultures. Biosens. Bioelectron..

[67] Grossmann H.K., Grieb T., Meierhofer F., Hodapp M.J., Noriler D., Gröhn A., Meier H.F., Fritsching U., Wegner K., Mädler L. (2015). Nanoscale Mixing during Double-Flame Spray Synthesis of Heterostructured Nanoparticles. J. Nanopart. Res..

[68] Solakidou M., Georgiou Y., Deligiannakis Y. (2021). Double-Nozzle Flame Spray Pyrolysis as a Potent Technology to Engineer Noble Metal-TiO_2_ Nanophotocatalysts for Efficient H_2_ Production. Energies.

[69] Tada S., Larmier K., Büchel R., Copéret C. (2018). Methanol Synthesis via CO_2_ Hydrogenation over CuO–ZrO_2_ Prepared by Two-Nozzle Flame Spray Pyrolysis. Catal. Sci. Technol..

[70] Li H., Erinmwingbovo C., Birkenstock J., Schowalter M., Rosenauer A., La Mantia F., Mädler L., Pokhrel S. (2021). Double Flame-Fabricated High-Performance AlPO_4_/LiMn_2_O_4_ Cathode Material for Li-Ion Batteries. ACS Appl. Energy Mater..

[71] Lovell E.C., Großman H., Horlyck J., Scott J., Mädler L., Amal R. (2019). Asymmetrical Double Flame Spray Pyrolysis-Designed SiO_2_/Ce_0.7_Zr_0.3_O_2_ for the Dry Reforming of Methane. ACS Appl. Mater. Interfaces.

[72] Psathas P., Moularas C., Smykała S., Deligiannakis Y. (2023). Highly Crystalline Nanosized NaTaO_3_/NiO Heterojunctions Engineered by Double-Nozzle Flame Spray Pyrolysis for Solar-to-H_2_ Conversion: Toward Industrial-Scale Synthesis. ACS Appl. Nano Mater..

[73] Gockeln M., Pokhrel S., Meierhofer F., Glenneberg J., Schowalter M., Rosenauer A., Fritsching U., Busse M., Mädler L., Kun R. (2018). Fabrication and Performance of Li_4_Ti_5_O_12_/C Li-Ion Battery Electrodes Using Combined Double Flame Spray Pyrolysis and Pressure-Based Lamination Technique. J. Power Sources.

[74] Hansen J.P., Jensen J.R., Livbjerg H., Johannessen T. (2001). Synthesis of ZnO Particles in a Quench-Cooled Flame Reactor. AIChE J..

[75] Teleki A., Heine M.C., Krumeich F., Akhtar M.K., Pratsinis S.E. (2008). In Situ Coating of Flame-Made TiO_2_ Particles with Nanothin SiO_2_ Films. Langmuir.

[76] Teleki A., Pratsinis S.E., Wegner K., Jossen R., Krumeich F. (2005). Flame-Coating of Titania Particles with Silica. J. Mater. Res..

[77] Teleki A., Buesser B., Heine M.C., Krumeich F., Akhtar M.K., Pratsinis S.E. (2009). Role of Gas−Aerosol Mixing during in Situ Coating of Flame-Made Titania Particles. Ind. Eng. Chem. Res..

[78] Sotiriou G.A., Sannomiya T., Teleki A., Krumeich F., Vörös J., Pratsinis S.E. (2010). Non-Toxic Dry-Coated Nanosilver for Plasmonic Biosensors. Adv. Funct. Mater..

[79] Sotiriou G., Gass S., Pratsinis S.E. (2012). Hermetically Coated Nanosilver: No Ag^+^ Ion Leaching. MRS Proc..

[80] Moularas C., Georgiou Y., Adamska K., Deligiannakis Y. (2019). Thermoplasmonic Heat Generation Efficiency by Nonmonodisperse Core–Shell Ag^0^@SiO_2_ Nanoparticle Ensemble. J. Phys. Chem. C.

[81] Moularas C., Dimitriou C., Georgiou Y., Evangelakis G., Boukos N., Deligiannakis Y. (2023). Electron Paramagnetic Resonance Quantifies Hot-Electron Transfer from Plasmonic Ag@SiO_2_ to Cr^6+^/Cr^5+^/Cr^3+^. J. Phys. Chem. C.

[82] Fragou F., Stathi P., Deligiannakis Y., Louloudi M. (2022). Safe-by-Design Flame Spray Pyrolysis of SiO_2_ Nanostructures for Minimizing Acute Toxicity. ACS Appl. Nano Mater..

[83] Li Y., Hu Y., Huo J., Jiang H., Li C., Huang G. (2012). Stable Core Shell Co_3_Fe_7_–CoFe_2_O_4_ Nanoparticles Synthesized via Flame Spray Pyrolysis Approach. Ind. Eng. Chem. Res..

[84] Jeanne-Rose V., Samain H., Deligiannakis Y., Louloudi M. (2023). Metal Oxide Particles Coated with a Rare-Earth Oxide and Process for Preparing Same by Flame Spray Pyrolysis. U.S. Patent.

[85] Zhou G., Zhang Y., Zhao X., Gui Y., Wang X., Li L., Chen T., Huang Z., Lin H. (2023). FSP Synthesized Core-Shell CuO_x_@SiO_2_ Catalyst with Excellent Thermal Stability for Catalytic Combustion of Ammonia. Fuel.

[86] Wu Z., Zhang Y., Zhao X., Wang H., Li S. (2021). Dual Liquid/Vapor-Fed Flame Synthesis for the Effective Preparation of SiO_2_@YAlO_3_:Nd^3+^ Nanophosphors. Proc. Combust. Inst..

[87] Sotiriou G.A., Schneider M., Pratsinis S.E. (2012). Green, Silica-Coated Monoclinic Y_2_O_3_:Tb^3+^ Nanophosphors: Flame Synthesis and Characterization. J. Phys. Chem. C.

[88] Sotiriou G.A., Blattmann C.O., Deligiannakis Y. (2016). Nanoantioxidant-Driven Plasmon Enhanced Proton-Coupled Electron Transfer. Nanoscale.

[89] Sahm T., Rong W., Barsan N., Madler L., Weimar U. (2007). Sensing of CH_4_, CO and Ethanol with in Situ Nanoparticle Aerosol-Fabricated Multilayer Sensors. Sens. Actuators B Chem..

[90] Sahm T., Rong W., Bârsan N., Mädler L., Friedlander S.K., Weimar U. (2007). Formation of Multilayer Films for Gas Sensing by in Situ Thermophoretic Deposition of Nanoparticles from Aerosol Phase. J. Mater. Res..

[91] Deligiannakis Y., Tsikourkitoudi V., Stathi P., Wegner K., Papavasiliou J., Louloudi M. (2020). PdO/Pd^0^/TiO_2_ Nanocatalysts Engineered by Flame Spray Pyrolysis: Study of the Synergy of PdO/Pd^0^ on H_2_ Production by HCOOH Dehydrogenation and the Deactivation Mechanism. Energy Fuels.

[92] Mädler L., Roessler A., Pratsinis S.E., Sahm T., Gurlo A., Barsan N., Weimar U. (2006). Direct Formation of Highly Porous Gas-Sensing Films by in Situ Thermophoretic Deposition of Flame-Made Pt/SnO_2_ Nanoparticles. Sens. Actuators B Chem..

[93] Nasiri N., Bo R., Wang F., Fu L., Tricoli A. (2015). Ultraporous Electron-Depleted ZnO Nanoparticle Networks for Highly Sensitive Portable Visible-Blind UV Photodetectors. Adv. Mater..

[94] Chen H., Mulmudi H.K., Tricoli A. (2020). Flame Spray Pyrolysis for the One-Step Fabrication of Transition Metal Oxide Films: Recent Progress in Electrochemical and Photoelectrochemical Water Splitting. Chin. Chem. Lett..

[95] Mane R.S., Lokhande C.D. (2000). Chemical Deposition Method for Metal Chalcogenide Thin Films. Mater. Chem. Phys..

[96] Kavitha R., Meghani S., Jayaram V. (2007). Synthesis of Titania Films by Combustion Flame Spray Pyrolysis Technique and Its Characterization for Photocatalysis. Mater. Sci. Eng. B.

[97] Kavitha R., Hegde S.R., Jayaram V. (2003). Oxide Films by Combustion Pyrolysis of Solution Precursors. Mater. Sci. Eng. A.

[98] Tricoli A., Elmøe T.D. (2012). Flame Spray Pyrolysis Synthesis and Aerosol Deposition of Nanoparticle Films. AIChE J..

[99] Tricoli A., Graf M., Mayer F., Kuühne S., Hierlemann A., Pratsinis S.E. (2008). Micropatterning Layers by Flame Aerosol Deposition-Annealing. Adv. Mater..

[100] Salameh S., Gómez-Hernández J., Goulas A., Van Bui H., van Ommen J.R. (2017). Advances in Scalable Gas-Phase Manufacturing and Processing of Nanostructured Solids: A Review. Particuology.

[101] https://www.Cabotcorp.Com/Solutions#products-Plus.

[102] https://Corporate.Evonik.Com/En/Products-and-Solutions/Markets.

[103] Kelesidis G.A., Pratsinis S.E. (2021). A Perspective on Gas-Phase Synthesis of Nanomaterials: Process Design, Impact and Outlook. Chem. Eng. J..

[104] https://www.Hemotune.Ch/Technology.

[105] https://www.Turbobeads.Com.

[106] https://www.Heiq.Com/Products/.

[107] Sotiris E.P., Height M. (2008). Antimicrobial and Antifungal Powders Made by Flame Spray Pyrolysis. U.S. Patent.

[108] Wegner K., Schimmöller B., Thiebaut B., Fernandez C., Rao T.N. (2011). Pilot Plants for Industrial Nanoparticle Production by Flame Spray Pyrolysis. KONA.

[109] Gerken L.R.H., Neuer A.L., Gschwend P.M., Keevend K., Gogos A., Anthis A.H.C., Aengenheister L., Pratsinis S.E., Plasswilm L., Herrmann I.K. (2021). Scalable Synthesis of Ultrasmall Metal Oxide Radio-Enhancers Outperforming Gold. Chem. Mater..

[110] Mädler L., Stark W.J., Pratsinis S.E. (2002). Flame-Made Ceria Nanoparticles. J. Mater. Res..

[111] Wegner K., Pratsinis S.E. (2005). Gas-Phase Synthesis of Nanoparticles: Scale-up and Design of Flame Reactors. Powder Technol..

[112] Wegner K., Pratsinis S.E. (2003). Scale-up of Nanoparticle Synthesis in Diffusion Flame Reactors. Chem. Eng. Sci..

[113] Akurati K.K., Vital A., Dellemann J.-P., Michalow K., Graule T., Ferri D., Baiker A. (2008). Flame-Made WO_3_/TiO_2_ Nanoparticles: Relation between Surface Acidity, Structure and Photocatalytic Activity. Appl. Catal. B Environ..

[114] Heel A., Holtappels P., Hug P., Graule T. (2010). Flame Spray Synthesis of Nanoscale La_0.6_Sr_0.4_Co_0.2_Fe_0.8_O_3−δ_ and Ba_0.5_Sr_0.5_Co_0.8_Fe_0.2_O_3−δ_ as Cathode Materials for Intermediate Temperature Solid Oxide Fuel Cells. Fuel Cells.

[115] Mueller R., Mädler L., Pratsinis S.E. (2003). Nanoparticle Synthesis at High Production Rates by Flame Spray Pyrolysis. Chem. Eng. Sci..

[116] Mueller R., Jossen R., Pratsinis S.E., Watson M., Akhtar M.K. (2004). Zirconia Nanoparticles Made in Spray Flames at High Production Rates. J. Am. Ceram. Soc..

[117] Gröhn A.J., Pratsinis S.E., Sánchez-Ferrer A., Mezzenga R., Wegner K. (2014). Scale-up of Nanoparticle Synthesis by Flame Spray Pyrolysis: The High-Temperature Particle Residence Time. Ind. Eng. Chem. Res..

[118] Meierhofer F., Mädler L., Fritsching U. (2020). Nanoparticle Evolution in Flame Spray Pyrolysis—Process Design via Experimental and Computational Analysis. AIChE J..

[119] Jossen R., Mueller R., Pratsinis S.E., Watson M., Akhtar M.K. (2005). Morphology and Composition of Spray-Flame-Made Yttria-Stabilized Zirconia Nanoparticles. Nanotechnology.

[120] Hembram K., Sivaprakasam D., Rao T.N., Wegner K. (2013). Large-Scale Manufacture of ZnO Nanorods by Flame Spray Pyrolysis. J. Nanopart. Res..

[121] Betancur-Granados N., Pöllmann H., Restrepo-Baena O.J., Tobón J.I. (2023). Nanosized Belite Phases Obtained by Flame Spray Pyrolysis: Assessment of Process Conditions on the Mineralogy and Reactivity. Cem. Concr. Res..

[122] Kho Y.K., Teoh W.Y., Iwase A., Mädler L., Kudo A., Amal R. (2011). Flame Preparation of Visible-Light-Responsive BiVO_4_ Oxygen Evolution Photocatalysts with Subsequent Activation via Aqueous Route. ACS Appl. Mater. Interfaces.

[123] Stathi P., Solakidou M., Deligiannakis Y. (2021). Lattice Defects Engineering in W-, Zr-Doped BiVO_4_ by Flame Spray Pyrolysis: Enhancing Photocatalytic O_2_ Evolution. Nanomaterials.

[124] Psathas P., Georgiou Y., Moularas C., Armatas G.S., Deligiannakis Y. (2020). Controlled-Phase Synthesis of Bi_2_Fe_4_O_9_ & BiFeO_3_ by Flame Spray Pyrolysis and Their Evaluation as Non-Noble Metal Catalysts for Efficient Reduction of 4-Nitrophenol. Powder Technol..

[125] Psathas P., Solakidou M., Mantzanis A., Deligiannakis Y. (2021). Flame Spray Pyrolysis Engineering of Nanosized Mullite-Bi_2_Fe_4_O_9_ and Perovskite-BiFeO_3_ as Highly Efficient Photocatalysts for O_2_ Production from H_2_O Splitting. Energies.

[126] Punginsang M., Wisitsoraat A., Tuantranont A., Phanichphant S., Liewhiran C. (2019). Ultrafine Bi_2_WO_6_ Nanoparticles Prepared by Flame Spray Pyrolysis for Selective Acetone Gas-Sensing. Mater. Sci. Semicond. Process..

[127] Xiao B., Jiao A., Zhang Y., Yang L., Zhao X., Wu C., Guo D., Zhan R., Lin H. (2021). Mixed Potential Type Ammonia Sensor Using Fe-Substituted LaCoO_3_ Sensing Electrode Prepared by Flame Spray Pyrolysis. Sens. Actuators B Chem..

[128] Jiao A., Zhang Y., Yang L., Zhao X., Wu C., Chen T., Zhan R., Huang Z., Lin H. (2023). Enhanced CO_2_ Response of La_1−x_FeO_3−δ_ Perovskites with A-Site Deficiency Synthesized by Flame Spray Pyrolysis. Ceram. Int..

[129] Yuan X., Meng L., Zheng C., Zhao H. (2021). Deep Insight into the Mechanism of Catalytic Combustion of CO and CH_4_ over SrTi_1− *x*_B*_x_*O_3_ (B = Co, Fe, Mn, Ni, and Cu) Perovskite via Flame Spray Pyrolysis. ACS Appl. Mater. Interfaces.

[130] Yuan X., Meng L., Xu Z., Zheng C., Zhao H. (2021). CuO Quantum Dots Supported by SrTiO_3_ Perovskite Using the Flame Spray Pyrolysis Method: Enhanced Activity and Excellent Thermal Resistance for Catalytic Combustion of CO and CH_4_. Environ. Sci. Technol..

[131] Yuan X., Zheng C., Zhang T., Li L., Yang Q., Zhao H. (2023). Sodium Doped SrTi_1−x_B_x_O_3_ (B = Mn, Co) for Formaldehyde Catalytic Oxidation: Flame Spray Pyrolysis Fabrication and Reaction Mechanism Elaboration. Fuel Process. Technol..

[132] Moularas C., Psathas P., Deligiannakis Y. (2021). Electron Paramagnetic Resonance Study of Photo-Induced Hole/Electron Pairs in NaTaO_3_ Nanoparticles. Chem. Phys. Lett..

[133] Kennedy A.E., Meekins B.H. (2018). Combustion Synthesis and Photoelectrochemical Characterization of Gallium Zinc Oxynitrides. J. Mater. Res..

[134] Huo J., Hu Y., Jiang H., Hou X., Li C. (2014). Continuous Flame Synthesis of near Surface Nitrogen Doped TiO_2_ for Dye-Sensitized Solar Cells. Chem. Eng. J..

[135] Bi W., Hu Y., Jiang H., Yu H., Li W., Li C. (2019). In-Situ Synthesized Surface N-Doped Pt/TiO_2_ via Flame Spray Pyrolysis with Enhanced Thermal Stability for CO Catalytic Oxidation. Appl. Surf. Sci..

[136] Boningari T., Inturi S.N.R., Suidan M., Smirniotis P.G. (2018). Novel Continuous Single-Step Synthesis of Nitrogen-Modified TiO_2_ by Flame Spray Pyrolysis for Photocatalytic Degradation of Phenol in Visible Light. J. Mater. Sci. Technol..

[137] Boningari T., Inturi S.N.R., Suidan M., Smirniotis P.G. (2018). Novel One-Step Synthesis of Nitrogen-Doped TiO_2_ by Flame Aerosol Technique for Visible-Light Photocatalysis: Effect of Synthesis Parameters and Secondary Nitrogen (N) Source. Chem. Eng. J..

[138] Smirniotis P.G., Boningari T., Damma D., Inturi S.N.R. (2018). Single-Step Rapid Aerosol Synthesis of N-Doped TiO_2_ for Enhanced Visible Light Photocatalytic Activity. Catal. Commun..

[139] Smirniotis P.G., Boningari T., Inturi S.N.R. (2018). Single-Step Synthesis of N-Doped TiO_2_ by Flame Aerosol Method and the Effect of Synthesis Parameters. Aerosol Sci. Technol..

[140] Herrmann I.K., Grass R.N., Mazunin D., Stark W.J. (2009). Synthesis and Covalent Surface Functionalization of Nonoxidic Iron Core−Shell Nanomagnets. Chem. Mater..

[141] Balakrishnan A., Groeneveld J.D., Pokhrel S., Mädler L. (2021). Metal Sulfide Nanoparticles: Precursor Chemistry. Chem. Eur. J..

[142] Bubenhofer S.B., Schumacher C.M., Koehler F.M., Luechinger N.A., Grass R.N., Stark W.J. (2012). Large-Scale Synthesis of PbS–TiO_2_ Heterojunction Nanoparticles in a Single Step for Solar Cell Application. J. Phys. Chem. C.

[143] Pokhrel S., Stahl J., Groeneveld J.D., Schowalter M., Rosenauer A., Birkenstock J., Mädler L. (2023). Flame Aerosol Synthesis of Metal Sulfides at High Temperature in Oxygen-Lean Atmosphere. Adv. Mater..

[144] Grass R.N., Stark W.J. (2005). Flame Synthesis of Calcium-, Strontium-, Barium Fluoride Nanoparticles and Sodium Chloride. Chem. Commun..

[145] Stepuk A., Krämer K.W., Stark W.J. (2013). Flame Synthesis of Complex Fluoride-Based Nanoparticles as Upconversion Phosphors. KONA Powder Part. J..

[146] Jodhani G., Mikaeili F., Gouma P.I. (2019). Flame Spray Synthesis of VOPO_4_ Polymorphs. Front. Mater..

[147] Rohner F., Ernst F.O., Arnold M., Hilbe M., Biebinger R., Ehrensperger F., Pratsinis S.E., Langhans W., Hurrell R.F., Zimmermann M.B. (2007). Synthesis, Characterization, and Bioavailability in Rats of Ferric Phosphate Nanoparticles. J. Nutr..

[148] Rudin T., Pratsinis S.E. (2012). Homogeneous Iron Phosphate Nanoparticles by Combustion of Sprays. Ind. Eng. Chem. Res..

[149] Waser O., Büchel R., Hintennach A., Novák P., Pratsinis S.E. (2011). Continuous Flame Aerosol Synthesis of Carbon-Coated Nano-LiFePO_4_ for Li-Ion Batteries. J. Aerosol Sci..

[150] Badding M.E., Brown J.L., Fekety C.R., Song Z. (2014). Corning Inc, Flame Spray Pyrolysis Method for Forming Nanoscale Lithium Metal Phosphate Powders. U.S. Patent.

[151] Cho J.S., Ko Y.N., Koo H.Y., Kang Y.C. (2010). Synthesis of Nano-Sized Biphasic Calcium Phosphate Ceramics with Spherical Shape by Flame Spray Pyrolysis. J. Mater. Sci. Mater. Med..

[152] Ataol S., Tezcaner A., Duygulu O., Keskin D., Machin N.E. (2015). Synthesis and Characterization of Nanosized Calcium Phosphates by Flame Spray Pyrolysis, and Their Effect on Osteogenic Differentiation of Stem Cells. J. Nanopart. Res..

[153] Tsikourkitoudi V., Karlsson J., Merkl P., Loh E., Henriques-Normark B., Sotiriou G.A. (2020). Flame-Made Calcium Phosphate Nanoparticles with High Drug Loading for Delivery of Biologics. Molecules.

[154] Huber M., Stark W.J., Loher S., Maciejewski M., Krumeich F., Baiker A. (2005). Flame Synthesis of Calcium Carbonate Nanoparticles. Chem. Commun..

[155] Strobel R., Maciejewski M., Pratsinis S.E., Baiker A. (2006). Unprecedented Formation of Metastable Monoclinic BaCO_3_ Nanoparticles. Thermochim. Acta.

[156] Li Y., Hu Y., Huang G., Li C. (2013). Metallic Iron Nanoparticles: Flame Synthesis, Characterization and Magnetic Properties. Particuology.

[157] Kang Y.C., Sohn J.R., Yoon H.S., Jung K.Y., Park H.D. (2003). Improved Photoluminescence of Sr_5_(PO_4_)_3_Cl:Eu^2+^ Phosphor Particles Prepared by Flame Spray Pyrolysis. J. Electrochem. Soc..

[158] Hilty F.M., Teleki A., Krumeich F., Büchel R., Hurrell R.F., Pratsinis S.E., Zimmermann M.B. (2009). Development and Optimization of Iron- and Zinc-Containing Nanostructured Powders for Nutritional Applications. Nanotechnology.

[159] Allen L., de Benoist B., Dary O., Hurrell R. (2006). Guidelines on Food Fortification with Micronutrients.

[160] Yamada A., Chung S.C., Hinokuma K. (2001). Optimized LiFePO_4_ for Lithium Battery Cathodes. J. Electrochem. Soc..

[161] Stark W., Pratsinis S., Maciejewski M., Loher S., Baiker A. (2014). Flame Synthesis of Metal Salt Nanoparticles, in Particular Calcium and Phosphate Comprising Nanoparticles. U.S. Patent.

[162] Soares S., Sousa J., Pais A., Vitorino C. (2018). Nanomedicine: Principles, Properties, and Regulatory Issues. Front. Chem..

[163] Ekimov A., Onushchehko A. (1981). Quantum Size Effect in Three-Dimensional Microscopic Semiconductor Crystals. ZhETF Pis Ma Redaktsiiu.

[164] Rossetti R., Brus L. (1982). Electron-Hole Recombination Emission as a Probe of Surface Chemistry in Aqueous Cadmium Sulfide Colloids. J. Phys. Chem..

[165] Murray C.B., Norris D.J., Bawendi M.G. (1993). Synthesis and Characterization of Nearly Monodisperse CdE (E = Sulfur, Selenium, Tellurium) Semiconductor Nanocrystallites. J. Am. Chem. Soc..

[166] Patial S., Sonu, Sudhaik A., Chandel N., Ahamad T., Raizada P., Singh P., Chaukura N., Selvasembian R. (2022). A Review on Carbon Quantum Dots Modified G-C_3_N_4_-Based Photocatalysts and Potential Application in Wastewater Treatment. Appl. Sci..

[167] Jang E., Jang H. (2023). Review: Quantum Dot Light-Emitting Diodes. Chem. Rev..

[168] García de Arquer F.P., Talapin D.V., Klimov V.I., Arakawa Y., Bayer M., Sargent E.H. (2021). Semiconductor Quantum Dots: Technological Progress and Future Challenges. Science.

[169] Cotta M.A. (2020). Quantum Dots and Their Applications: What Lies Ahead?. ACS Appl. Nano Mater..

[170] Bera D., Qian L., Tseng T.-K., Holloway P.H. (2010). Quantum Dots and Their Multimodal Applications: A Review. Materials.

[171] Alivisatos A.P. (1996). Semiconductor Clusters, Nanocrystals, and Quantum Dots. Science.

[172] Edvinsson T. (2018). Optical Quantum Confinement and Photocatalytic Properties in Two-, One- and Zero-Dimensional Nanostructures. R. Soc. Open Sci..

[173] Bajorowicz B., Kobylański M.P., Gołąbiewska A., Nadolna J., Zaleska-Medynska A., Malankowska A. (2018). Quantum Dot-Decorated Semiconductor Micro- and Nanoparticles: A Review of Their Synthesis, Characterization and Application in Photocatalysis. Adv. Colloid Interface Sci..

[174] Kaxiras E., Joannopoulos J.D. (2019). Quantum Theory of Materials.

[175] Dieleman C.D., Ding W., Wu L., Thakur N., Bespalov I., Daiber B., Ekinci Y., Castellanos S., Ehrler B. (2020). Universal Direct Patterning of Colloidal Quantum Dots by (Extreme) Ultraviolet and Electron Beam Lithography. Nanoscale.

[176] Palankar R., Medvedev N., Rong A., Delcea M. (2013). Fabrication of Quantum Dot Microarrays Using Electron Beam Lithography for Applications in Analyte Sensing and Cellular Dynamics. ACS Nano.

[177] Li Q.-L., Shi L.-X., Du K., Qin Y., Qu S.-J., Xia D.-Q., Zhou Z., Huang Z.-G., Ding S.-N. (2020). Copper-Ion-Assisted Precipitation Etching Method for the Luminescent Enhanced Assembling of Sulfur Quantum Dots. ACS Omega.

[178] Tsutsui K., Hu E.L., Wilkinson C.D.W. (1993). Reactive Ion Etched II-VI Quantum Dots: Dependence of Etched Profile on Pattern Geometry. Jpn. J. Appl. Phys..

[179] Arachchige I.U., Brock S.L. (2007). Sol–Gel Methods for the Assembly of Metal Chalcogenide Quantum Dots. Acc. Chem. Res..

[180] Spanhel L., Anderson M.A. (1991). Semiconductor Clusters in the Sol-Gel Process: Quantized Aggregation, Gelation, and Crystal Growth in Concentrated Zinc Oxide Colloids. J. Am. Chem. Soc..

[181] Lin K.-F., Cheng H.-M., Hsu H.-C., Lin L.-J., Hsieh W.-F. (2005). Band Gap Variation of Size-Controlled ZnO Quantum Dots Synthesized by Sol–Gel Method. Chem. Phys. Lett..

[182] Darbandi M., Thomann R., Nann T. (2005). Single Quantum Dots in Silica Spheres by Microemulsion Synthesis. Chem. Mater..

[183] Koole R., van Schooneveld M.M., Hilhorst J., de Mello Donegá C., Hart D.C., van Blaaderen A., Vanmaekelbergh D., Meijerink A. (2008). On the Incorporation Mechanism of Hydrophobic Quantum Dots in Silica Spheres by a Reverse Microemulsion Method. Chem. Mater..

[184] Wenger W.N., Bates F.S., Aydil E.S. (2017). Functionalization of Cadmium Selenide Quantum Dots with Poly(Ethylene Glycol): Ligand Exchange, Surface Coverage, and Dispersion Stability. Langmuir.

[185] Xin S.H., Wang P.D., Yin A., Kim C., Dobrowolska M., Merz J.L., Furdyna J.K. (1996). Formation of Self-assembling CdSe Quantum Dots on ZnSe by Molecular Beam Epitaxy. Appl. Phys. Lett..

[186] Facsko S., Dekorsy T., Koerdt C., Trappe C., Kurz H., Vogt A., Hartnagel H.L. (1999). Formation of Ordered Nanoscale Semiconductor Dots by Ion Sputtering. Science.

[187] Oshinowo J., Nishioka M., Ishida S., Arakawa Y. (1994). Highly Uniform InGaAs/GaAs Quantum Dots (~15 nm) by Metalorganic Chemical Vapor Deposition. Appl. Phys. Lett..

[188] Mädler L., Stark W.J., Pratsinis S.E. (2002). Rapid Synthesis of Stable ZnO Quantum Dots. J. Appl. Phys..

[189] Riad K.B., Hoa S.V., Wood-Adams P.M. (2021). Metal Oxide Quantum Dots Embedded in Silica Matrices Made by Flame Spray Pyrolysis. ACS Omega.

[190] Bi W., Hu Y., Jiang H., Lei J., Wan X., Zhang L., Li C. (2021). Flame Process Constructing CQDs/TiO_2_-C Heterostructure with Novel Electron Transfer Channel between Internal and External Carbon Species. Combust. Flame.

[191] Teck M., Murshed M.M., Schowalter M., Lefeld N., Grossmann H.K., Grieb T., Hartmann T., Robben L., Rosenauer A., Mädler L. (2017). Structural and Spectroscopic Comparison between Polycrystalline, Nanocrystalline and Quantum Dot Visible Light Photo-Catalyst Bi_2_WO_6_. J. Solid State Chem..

[192] Maier S.A. (2007). Plasmonics: Fundamentals and Applications.

[193] Kooyman R.P.H., Corn R.M., Wark A., Lee H.J., Gedig E., Engbers G., Walstrom L., de Mol N.J., Hall D.R., Yager P. (2008). Handbook of Surface Plasmon Resonance.

[194] Kreibig U., Vollmer M. (1995). Optical Properties of Metal Clusters.

[195] Absorption and Scattering of Light by Small Particles|Wiley. https://www.wiley.com/en-us/Absorption+and+Scattering+of+Light+by+Small+Particles-p-9780471293408.

[196] Brongersma M.L., Halas N.J., Nordlander P. (2015). Plasmon-Induced Hot Carrier Science and Technology. Nat. Nanotechnol..

[197] Halas N.J., Lal S., Chang W.-S., Link S., Nordlander P. (2011). Plasmons in Strongly Coupled Metallic Nanostructures. Chem. Rev..

[198] Baffou G., Quidant R. (2013). Thermo-Plasmonics: Using Metallic Nanostructures as Nano-Sources of Heat. Laser Photonics Rev..

[199] Pines D., Bohm D. (1952). A Collective Description of Electron Interactions: II. Collective vs Individual Particle Aspects of the Interactions. Phys. Rev..

[200] Bohm D., Pines D. (1953). A Collective Description of Electron Interactions: III. Coulomb Interactions in a Degenerate Electron Gas. Phys. Rev..

[201] Jackson J.D. (1998). Classical Electrodynamics.

[202] Sotiriou G.A., Pratsinis S.E. (2010). Antibacterial Activity of Nanosilver Ions and Particles. Environ. Sci. Technol..

[203] Sotiriou G.A., Starsich F., Dasargyri A., Wurnig M.C., Krumeich F., Boss A., Leroux J.-C., Pratsinis S.E. (2014). Photothermal Killing of Cancer Cells by the Controlled Plasmonic Coupling of Silica-Coated Au/Fe_2_O_3_ Nanoaggregates. Adv. Funct. Mater..

[204] Sotiriou G.A., Hirt A.M., Lozach P.-Y., Teleki A., Krumeich F., Pratsinis S.E. (2011). Hybrid, Silica-Coated, Janus-Like Plasmonic-Magnetic Nanoparticles. Chem. Mater..

[205] Johannessen T., Jensen J.R., Mosleh M., Johansen J., Quaade U., Livbjerg H. (2004). Flame Synthesis of Nanoparticles: Applications in Catalysis and Product/Process Engineering. Chem. Eng. Res. Des..

[206] Hannemann S., Grunwaldt J.-D., Krumeich F., Kappen P., Baiker A. (2006). Electron Microscopy and EXAFS Studies on Oxide-Supported Gold–Silver Nanoparticles Prepared by Flame Spray Pyrolysis. Appl. Surf. Sci..

[207] Sotiriou G.A., Blattmann C.O., Pratsinis S.E. (2012). Composite Nanosilver Structures Suitable for Plasmonic Biosensors. MRS Online Proc. Libr..

[208] Sotiriou G.A., Teleki A., Camenzind A., Krumeich F., Meyer A., Panke S., Pratsinis S.E. (2011). Nanosilver on Nanostructured Silica: Antibacterial Activity and Ag Surface Area. Chem. Eng. J..

[209] Chiarello G.L., Selli E., Forni L. (2008). Photocatalytic Hydrogen Production over Flame Spray Pyrolysis-Synthesised TiO_2_ and Au/TiO_2_. Appl. Catal. B Environ..

[210] Sotiriou G.A., Etterlin G.D., Spyrogianni A., Krumeich F., Leroux J.-C., Pratsinis S.E. (2014). Plasmonic Biocompatible Silver–Gold Alloyed Nanoparticles. Chem. Commun..

[211] Hu Y., Shi Y., Jiang H., Huang G., Li C. (2013). Scalable Preparation of Ultrathin Silica-Coated Ag Nanoparticles for SERS Application. ACS Appl. Mater. Interfaces.

[212] Fujiwara K., Deligiannakis Y., Skoutelis C.G., Pratsinis S.E. (2014). Visible-Light Active Black TiO_2_-Ag/TiO_x_ Particles. Appl. Catal. B Environ..

[213] Loher S., Schneider O.D., Maienfisch T., Bokorny S., Stark W.J. (2008). Micro-Organism-Triggered Release of Silver Nanoparticles from Biodegradable Oxide Carriers Allows Preparation of Self-Sterilizing Polymer Surfaces. Small.

[214] Ru E.C.L., Etchegoin P.G. (2014). Principles of Surface-Enhanced Raman Spectroscopy: And Related Plasmonic Effects.

[215] Xu Y., Zhang Y., Li C., Ye Z., Bell S.E.J. (2023). SERS as a Probe of Surface Chemistry Enabled by Surface-Accessible Plasmonic Nanomaterials. Acc. Chem. Res..

[216] Mahan J.E. (2000). Physical Vapor Deposition of Thin Films.

[217] Shi Y., Hamsen C., Jia X., Kim K.K., Reina A., Hofmann M., Hsu A.L., Zhang K., Li H., Juang Z.-Y. (2010). Synthesis of Few-Layer Hexagonal Boron Nitride Thin Film by Chemical Vapor Deposition. Nano Lett..

[218] Znaidi L. (2010). Sol–Gel-Deposited ZnO Thin Films: A Review. Mater. Sci. Eng. B.

[219] Jang W.-S., Rawson I., Grunlan J.C. (2008). Layer-by-Layer Assembly of Thin Film Oxygen Barrier. Thin Solid Films.

[220] Sahu N., Parija B., Panigrahi S. (2009). Fundamental Understanding and Modeling of Spin Coating Process: A Review. Indian J. Phys..

[221] Petty M.C. (1996). Langmuir-Blodgett Films: An Introduction.

[222] Nair P.K., Nair M.T.S., García V.M., Arenas O.L., Peña A.C.Y., Ayala I.T., Gomezdaza O., Sánchez A., Campos J., Hu H. (1998). Semiconductor Thin Films by Chemical Bath Deposition for Solar Energy Related Applications. Sol. Energy Mater. Sol. Cells.

[223] Karageorgakis N.I., Heel A., Bieberle-Hütter A., Rupp J.L.M., Graule T., Gauckler L.J. (2010). Flame Spray Deposition of La_0.6_Sr_0.4_CoO_3−δ_ Thin Films: Microstructural Characterization, Electrochemical Performance and Degradation. J. Power Sources.

[224] Blattmann C.O., Sotiriou G.A., Pratsinis S.E. (2015). Rapid Synthesis of Flexible Conductive Polymer Nanocomposite Films. Nanotechnology.

[225] Tricoli A., Nasiri N., Chen H., Wallerand A.S., Righettoni M. (2016). Ultra-Rapid Synthesis of Highly Porous and Robust Hierarchical ZnO Films for Dye Sensitized Solar Cells. Sol. Energy.

[226] Chen H., Bo R., Tran-Phu T., Liu G., Tricoli A. (2018). One-Step Rapid and Scalable Flame Synthesis of Efficient WO_3_ Photoanodes for Water Splitting. ChemPlusChem.

[227] Tran-Phu T., Chen H., Bo R., Bernardo I.D., Fusco Z., Simonov A.N., Tricoli A. (2019). High-Temperature One-Step Synthesis of Efficient Nanostructured Bismuth Vanadate Photoanodes for Water Oxidation. Energy Technol..

[228] Chakraborty D., Bischoff H., Chorkendorff I., Johannessen T. (2005). Mixed Phase Pt-Ru Catalyst for Direct Methanol Fuel Cell Anode by Flame Aerosol Synthesis. J. Electrochem. Soc..

[229] Li H., Dumont E., Slipets R., Thersleff T., Boisen A., Sotiriou G.A. (2023). Democratizing Robust SERS Nano-Sensors for Food Safety Diagnostics. Chem. Eng. J..

[230] Bletsa E., Merkl P., Thersleff T., Normark S., Henriques-Normark B., Sotiriou G.A. (2023). Highly Durable Photocatalytic Titanium Suboxide–Polymer Nanocomposite Films with Visible Light-Triggered Antibiofilm Activity. Chem. Eng. J..

[231] Kemmler J.A., Pokhrel S., Mädler L., Weimar U., Barsan N. (2013). Flame Spray Pyrolysis for Sensing at the Nanoscale. Nanotechnology.

[232] Tricoli A., Righettoni M., Teleki A. (2010). Semiconductor Gas Sensors: Dry Synthesis and Application. Angew. Chem. Int. Ed..

[233] Righettoni M., Amann A., Pratsinis S.E. (2015). Breath Analysis by Nanostructured Metal Oxides as Chemo-Resistive Gas Sensors. Mater. Today.

[234] Güntner A.T., Abegg S., Königstein K., Gerber P.A., Schmidt-Trucksäss A., Pratsinis S.E. (2019). Breath Sensors for Health Monitoring. ACS Sens..

[235] Sheng Y., Kraft M., Xu R. (2018). Emerging Applications of Nanocatalysts Synthesized by Flame Aerosol Processes. Curr. Opin. Chem. Eng..

[236] Khamfoo K., Inyawilert K., Wisitsoraat A., Tuantranont A., Phanichphant S., Liewhiran C. (2020). Formaldehyde Sensor Based on FSP-Made AgO_x_-Doped SnO_2_ Nanoparticulate Sensing Films. Sens. Actuators B Chem..

[237] Kaewsiri D., Inyawilert K., Wisitsoraat A., Tuantranont A., Phanichphant S., Liewhiran C. (2020). Flame-Spray-Made PtO_x_-Functionalized Zn_2_SnO_4_ Spinel Nanostructures for Conductometric H2 Detection. Sens. Actuators B Chem..

[238] Khamfoo K., Wisitsoraat A., Punginsang M., Tuantranont A., Liewhiran C. (2021). Selectivity towards Acetylene Gas of Flame-Spray-Made Nb-Substituted SnO_2_ Particulate Thick Films. Sens. Actuators B Chem..

[239] Inyawilert K., Sukee A., Siriwalai M., Wisitsoraat A., Sukunta J., Tuantranont A., Phanichphant S., Liewhiran C. (2021). Effect of Er Doping on Flame-Made SnO_2_ Nanoparticles to Ethylene Oxide Sensing. Sens. Actuators B Chem..

[240] Inyawilert K., Punginsang M., Wisitsoraat A., Tuantranont A., Liewhiran C. (2022). Graphene/Rh-Doped SnO_2_ Nanocomposites Synthesized by Electrochemical Exfoliation and Flame Spray Pyrolysis for H2S Sensing. J. Alloys Compd..

[241] Andreev M., Topchiy M., Asachenko A., Beltiukov A., Amelichev V., Sagitova A., Maksimov S., Smirnov A., Rumyantseva M., Krivetskiy V. (2022). Electrical and Gas Sensor Properties of Nb(V) Doped Nanocrystalline β-Ga_2_O_3_. Materials.

[242] Siriwalai M., Punginsang M., Inyawilert K., Wisitsoraat A., Tuantranont A., Liewhiran C. (2023). Flame-Spray-Synthesized La_2_O_3_-Loaded WO_3_ Nanoparticle Films for NO_2_ Sensing. ACS Appl. Nano Mater..

[243] Punginsang M., Inyawilert K., Siriwalai M., Wisitsoraat A., Tuantranont A., Liewhiran C. (2023). Comparative Study on Formic Acid Sensing Properties of Flame-Made Zn_2_SnO_4_ Nanoparticles and Its Parent Metal Oxides. Phys. Chem. Chem. Phys..

[244] Debecker D.P., Bras S.L., Boissière C., Chaumonnot A., Sanchez C. (2018). Aerosol Processing: A Wind of Innovation in the Field of Advanced Heterogeneous Catalysts. Chem. Soc. Rev..

[245] Belles L., Moularas C., Smykała S., Deligiannakis Y. (2021). Flame Spray Pyrolysis Co_3_O_4_/CoO as Highly-Efficient Nanocatalyst for Oxygen Reduction Reaction. Nanomaterials.

[246] Pozio A., Bozza F., Lisi N., Chierchia R., Migliorini F., Dondè R., De Iuliis S. (2022). Cobalt Oxide Synthesis via Flame Spray Pyrolysis as Anode Electrocatalyst for Alkaline Membrane Water Electrolyzer. Materials.

[247] Tran-Phu T., Daiyan R., Leverett J., Fusco Z., Tadich A., Bernardo I.D., Kiy A., Truong T.N., Zhang Q., Chen H. (2022). Understanding the Activity and Stability of Flame-Made Co_3_O_4_ Spinels: A Route towards the Scalable Production of Highly Performing OER Electrocatalysts. Chem. Eng. J..

[248] Liu G., Karuturi S.K., Simonov A.N., Fekete M., Chen H., Nasiri N., Le N.H., Reddy Narangari P., Lysevych M., Gengenbach T.R. (2016). Robust Sub-Monolayers of Co_3_O_4_ Nano-Islands: A Highly Transparent Morphology for Efficient Water Oxidation Catalysis. Adv. Energy Mater..

[249] Daiyan R., Tran-Phu T., Kumar P., Iputera K., Tong Z., Leverett J., Khan M.H.A., Esmailpour A.A., Jalili A., Lim M. (2021). Nitrate Reduction to Ammonium: From CuO Defect Engineering to Waste NO_x_-to-NH_3_ Economic Feasibility. Energy Environ. Sci..

[250] Tran-Phu T., Daiyan R., Fusco Z., Ma Z., Rahim L.R.A., Kiy A., Kluth P., Guo X., Zhu Y., Chen H. (2020). Multifunctional Nanostructures of Au–Bi_2_O_3_ Fractals for CO_2_ Reduction and Optical Sensing. J. Mater. Chem. A.

[251] Tran-Phu T., Daiyan R., Fusco Z., Ma Z., Amal R., Tricoli A. (2020). Nanostructured β-Bi_2_O_3_ Fractals on Carbon Fibers for Highly Selective CO_2_ Electroreduction to Formate. Adv. Funct. Mater..

[252] Sharaf O.Z., Orhan M.F. (2014). An Overview of Fuel Cell Technology: Fundamentals and Applications. Renew. Sustain. Energy Rev..

[253] Seo D.J., Ryu K.O., Park S.B., Kim K.Y., Song R.-H. (2006). Synthesis and Properties of Ce_1−x_Gd_x_O_2−x/2_ Solid Solution Prepared by Flame Spray Pyrolysis. Mater. Res. Bull..

[254] Lee H., Kim T.J., Li C., Choi I.D., Kim Y.T., Coker Z., Choi T.-Y., Lee D. (2014). Flame Aerosol Synthesis of Carbon-Supported Pt–Ru Catalysts for a Fuel Cell Electrode. Int. J. Hydrogen Energy.

[255] Tawonezvi T., Nomnqa M., Petrik L., Bladergroen B.J. (2023). Recovery and Recycling of Valuable Metals from Spent Lithium-Ion Batteries: A Comprehensive Review and Analysis. Energies.

[256] Gockeln M., Glenneberg J., Busse M., Pokhrel S., Mädler L., Kun R. (2018). Flame Aerosol Deposited Li_4_Ti_5_O_12_ Layers for Flexible, Thin Film All-Solid-State Li-Ion Batteries. Nano Energy.

[257] Nitta N., Wu F., Lee J.T., Yushin G. (2015). Li-Ion Battery Materials: Present and Future. Mater. Today.

[258] Jung D.S., Park S.B., Kang Y.C. (2010). Design of Particles by Spray Pyrolysis and Recent Progress in Its Application. Korean J. Chem. Eng..

[259] Kammler H.K., Mädler L., Pratsinis S.E. (2001). Flame Synthesis of Nanoparticles. Chem. Eng. Technol..

[260] Pan J., Libera J.A., Paulson N.H., Stan M. (2021). Flame Stability Analysis of Flame Spray Pyrolysis by Artificial Intelligence. Int. J. Adv. Manuf. Technol..

